# Population genetic analysis of *Plasmodium knowlesi* reveals differential selection and exchange events between Borneo and Peninsular sub-populations

**DOI:** 10.1038/s41598-023-29368-4

**Published:** 2023-02-07

**Authors:** Anna Turkiewicz, Emilia Manko, Damiola R. Oresegun, Debbie Nolder, Anton Spadar, Colin J. Sutherland, Janet Cox-Singh, Robert W. Moon, Yee-Ling Lau, Susana Campino, Taane G. Clark

**Affiliations:** 1 Faculty of Infectious and Tropical Diseases, London School of Hygiene and Tropical Medicine, London, WC1E 7HT UK; 2 Division of Infection, School of Medicine, University of St Andrews, St Andrews, UK; 3 UK Health Security Agency Malaria Reference Laboratory, London School of Hygiene and Tropical Medicine, London, WC1E 7HT UK; 4 Universiti Malaya, Kuala Lumpur, Malaysia; 5 Faculty of Epidemiology and Population Health, London School of Hygiene and Tropical Medicine, London, WC1E 7HT UK

**Keywords:** Population genetics, Genetic variation

## Abstract

The zoonotic *Plasmodium knowlesi* parasite is a growing public health concern in Southeast Asia, especially in Malaysia, where elimination of *P. falciparum* and *P. vivax* malaria has been the focus of control efforts. Understanding of the genetic diversity of *P. knowlesi* parasites can provide insights into its evolution, population structure, diagnostics, transmission dynamics, and the emergence of drug resistance. Previous work has revealed that *P. knowlesi* fall into three main sub-populations distinguished by a combination of geographical location and macaque host (*Macaca fascicularis* and *M. nemestrina*). It has been shown that Malaysian Borneo groups display profound heterogeneity with long regions of high or low divergence resulting in mosaic patterns between sub-populations, with some evidence of chromosomal-segment exchanges. However, the genetic structure of non-Borneo sub-populations is less clear. By gathering one of the largest collections of *P. knowlesi* whole-genome sequencing data, we studied structural genomic changes across sub-populations, with the analysis revealing differences in Borneo clusters linked to mosquito-related stages of the parasite cycle, in contrast to differences in host-related stages for the Peninsular group. Our work identifies new genetic exchange events, including introgressions between Malaysian Peninsular and *M. nemestrina*-associated clusters on various chromosomes, including in parasite invasion genes (DBP$\beta$, NBPX$\alpha$ and NBPX$\beta$), and important proteins expressed in the vertebrate parasite stages. Recombination events appear to have occurred between the Peninsular and *M. fascicularis*-associated groups, including in the DBP$\beta$ and DBP$\gamma$ invasion associated genes. Overall, our work finds that genetic exchange events have occurred among the recognised contemporary groups of *P. knowlesi* parasites during their evolutionary history, leading to apparent mosaicism between these sub-populations. These findings generate new hypotheses relevant to parasite evolutionary biology and *P. knowlesi* epidemiology, which can inform malaria control approaches to containing the impact of zoonotic malaria on human communities.

## Introduction

*Plasmodium knowlesi* is a zoonotic malaria parasite commonly residing in long-tailed (*Macaca fascicularis*) and pig-tailed (*M. nemestrina*) macaques and monkeys (*Presbytis melalophos*). The parasite is also recognized as a significant cause of human malaria, with cases described across most countries in the Southeast Asia and Western Pacific regions^1^. There is especially high burden in Malaysia, where *P. knowlesi* is the remaining malaria species with increasing numbers of cases reported^2,3^. Severe disease develops in 19% of clinical presentations^4^, while fatalities have been observed in 0.3–1.8% of reported cases in Malaysia^2^. Serological assessments of exposure to *P. knowlesi* in endemic Borneo (Kudat) found evidence of previous infection in 7% of the population^5^.

The vectors accountable for *P. knowlesi* transmission in Malaysia are members of the *Anopheles Leucosphyrus* group, wherein *An. latens* and *An. balbacensis* are primarily reported in Borneo Island, although recent studies have also confirmed the presence of *An. dondali*^6^. Evidence also suggests that *An. collessi* and *An. roperi*, members of the *Anopheles Umbrosus* group (endemic to Southeast Asia and India), infected with *P. knowlesi* are circulating in previously under-studied areas of Sarawak^7^. In Peninsular Malaysia, *An. hackeri* and *An. cracens* are known vectors^8^.

Deforestation and environmental changes associated with the rapid human population growth and development are thought to underlie *P. knowlesi* becoming the predominant cause of human malaria in Malaysia^9^. Malaria elimination strategies across the Southeast Asia region have focused primarily on *P. falciparum* and *P. vivax*, but as shown in South America^10^, neglected *P. vivax* persists long beyond *P. falciparum* decline and elimination. Reducing *P. knowlesi* malaria cases in human communities is hindered by the challenges to conventional control methods, including increased contact between people, mosquito vectors and *P. knowlesi* main primate reservoirs, influencing population dynamics and behaviour of both hosts and vectors^11^.

The adaptation of *P. knowlesi* to environmental changes, which in turn affect abundance of both insect and primate hosts, may be observed through analysis of genetic diversity and population structure. Understanding the genomic diversity of *P. knowlesi* using whole-genome sequencing (WGS) data can help reveal transmission patterns and inform disease control. It is now possible to sequence *P. knowlesi* DNA sourced from low parasitaemia infections using a parasite selective whole genome amplification approach^12^. Analysis of WGS data has identified three main clusters determined by location as being either Peninsular Malaysia (Pen-Pk) or Borneo, where in the latter, two groups may be distinguished by association with the respective host *Macaca* species (Mf-Pk - *M. fascicularis*; Mn-Pk - *M. nemestrina*)^13,14^. Genomic dimorphism among the groups have been revealed^15^. Moreover, previous analysis has provided evidence of genetic links between the Borneo populations, and identified chromosomal-segment exchanges spread throughout one of the chromosomes^14^. Utilising the largest collection of *P. knowlesi* WGS data enriched with laboratory samples cultured in *M. fascicularis* blood, we performed a series of population genetic analyses to identify recent evolutionary events and expand the current knowledge of parasite population structure. Evidence of new genetic exchange events between Peninsular (Pen-Pk) and Mn-associated (Mn-Pk) clusters were identified, and novel changes among Borneo groups have been revealed. These discoveries can contribute to an improved understanding of the mosaic genomic architecture observed across all three sub-populations.

## Results

### High levels of genomic variation and three sub-populations

**Figure 1:**
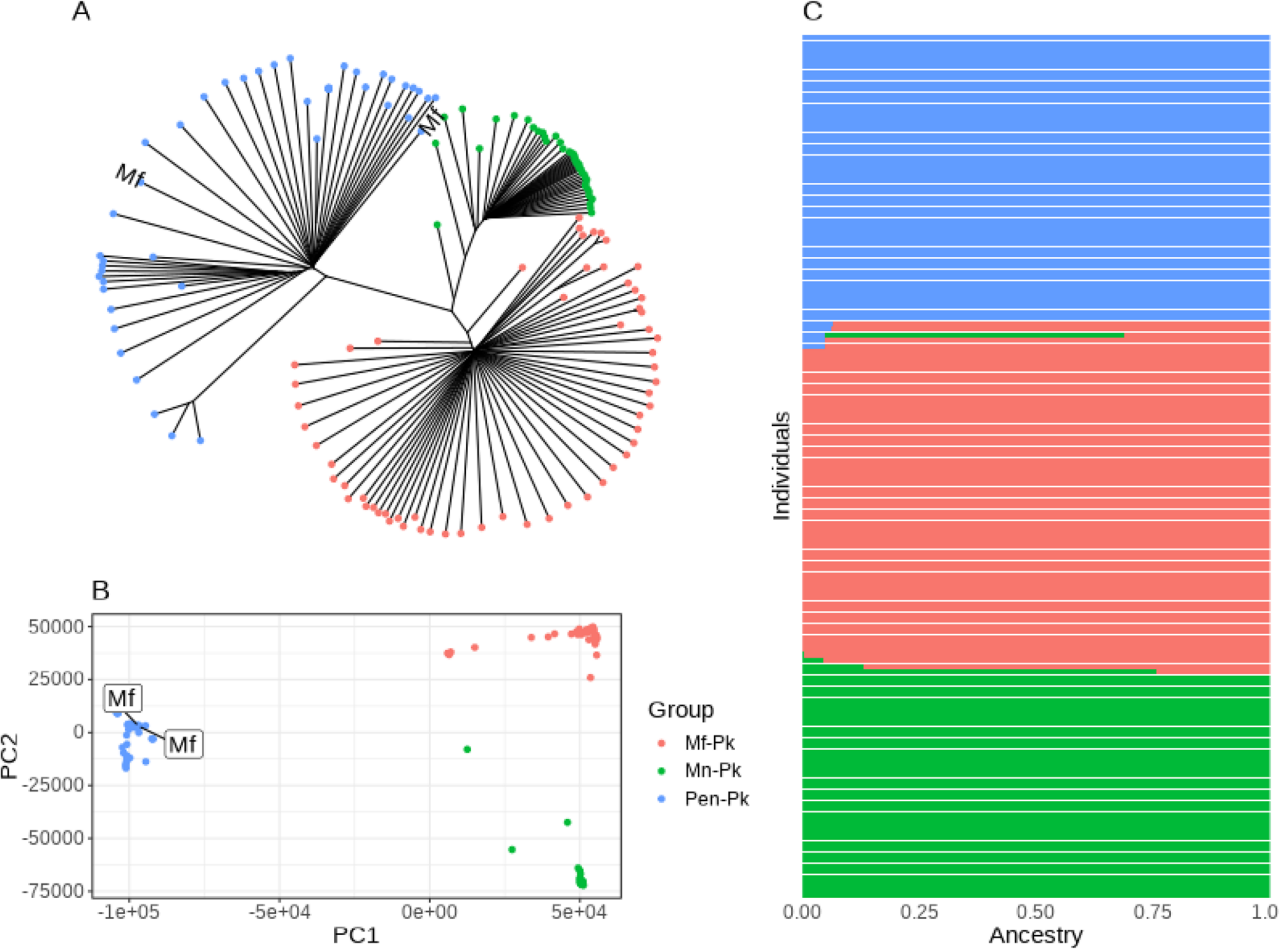
**P**opulation structure analysis using the whole set of 151 *P. knowlesi* isolates. (**A**) Neighbour-joining tree, with leaves coloured based on grouping; (**B**) Principal Component Analysis (PCA) plot, and samples collected from *M. fascicularis* have been marked; (**C**) ADMIXTURE barplot with predictions for each isolate reflecting the relative abundance of three ancestral sub-populations (K=3), with the color scheme the same as (**A**).

The analysed data consists of 151 isolates, primarily derived from human infections (n = 149) including field and laboratory samples, but also 2 isolates collected from cultured *M. fascicularis*. Across the dataset, there were 1,883,700 high quality genome-wide SNPs ($\approx$1 every 13 bp) identified, including 95 SNPs on the mitochondrial and 640 SNPs on the apicoplast organelle genomes. Within-infection genomic diversity revealed 124 (82.1%) isolates with mono-infections (Mf-Pk, n = 48; Mn-Pk, n = 31; Pen-Pk, n = 45) (S1 Figure), especially prevalent amongst human infections and, as expected, in laboratory strains. A co-infection with *P. vivax* was identified. The principal component analysis (PCA) and neighbour-joining tree reinforced the presence of three clusters, and all newly sequenced isolates (n = 25) fell within one of these groups (Fig. 1A, B). In particular, the macaque and human laboratory blood cultured strains segregated within the Peninsular cluster (Pen-Pk, n = 13). Whereas remaining newly sequenced isolates were identified as belonging to one of the two Borneo groups (Mf-Pk, n = 7; Mn-Pk, n = 5), including the single isolate from Indonesia (PK3), which was linked to the Mf-Pk cluster.

Estimation of individual ancestry using ADMIXTURE software revealed the existence of three ancestral backgrounds (Fig. 1C), consistent with the three PCA-based clusters (Mf-Pk, Mn-Pk, Pen-Pk). Most isolates could be assigned to a single ancestral group, except for three samples isolated from Sarikei, which displayed ancestry from both Borneo clusters. Most isolates were genetically distinct from one another, except for the newly derived human blood-cultured isolates, which were closely related, together with three publicly available clinical isolates (SRR3135172, SRR2222335, SRR2225467). Single representatives from the highly similar groups and all remaining isolates provided a total of 104 genomes available for subsequent population genetics analysis (Mf-Pk, n = 43; Mn-Pk, n = 30; Pen-Pk, n = 31). The overall nucleotide diversity for the Peninsular cluster ($\pi = 6.60 \times 10^{-3}$) and Mf-associated group ($\pi = 5.95 \times 10^{-3}$) were similar, while the Mn-associated samples had relatively lower genetic diversity ($\pi = 3.48 \times 10^{-3}$).

### Identity by descent analysis reveals differences between Borneo and Peninsular Malaysia

**Figure 2:**
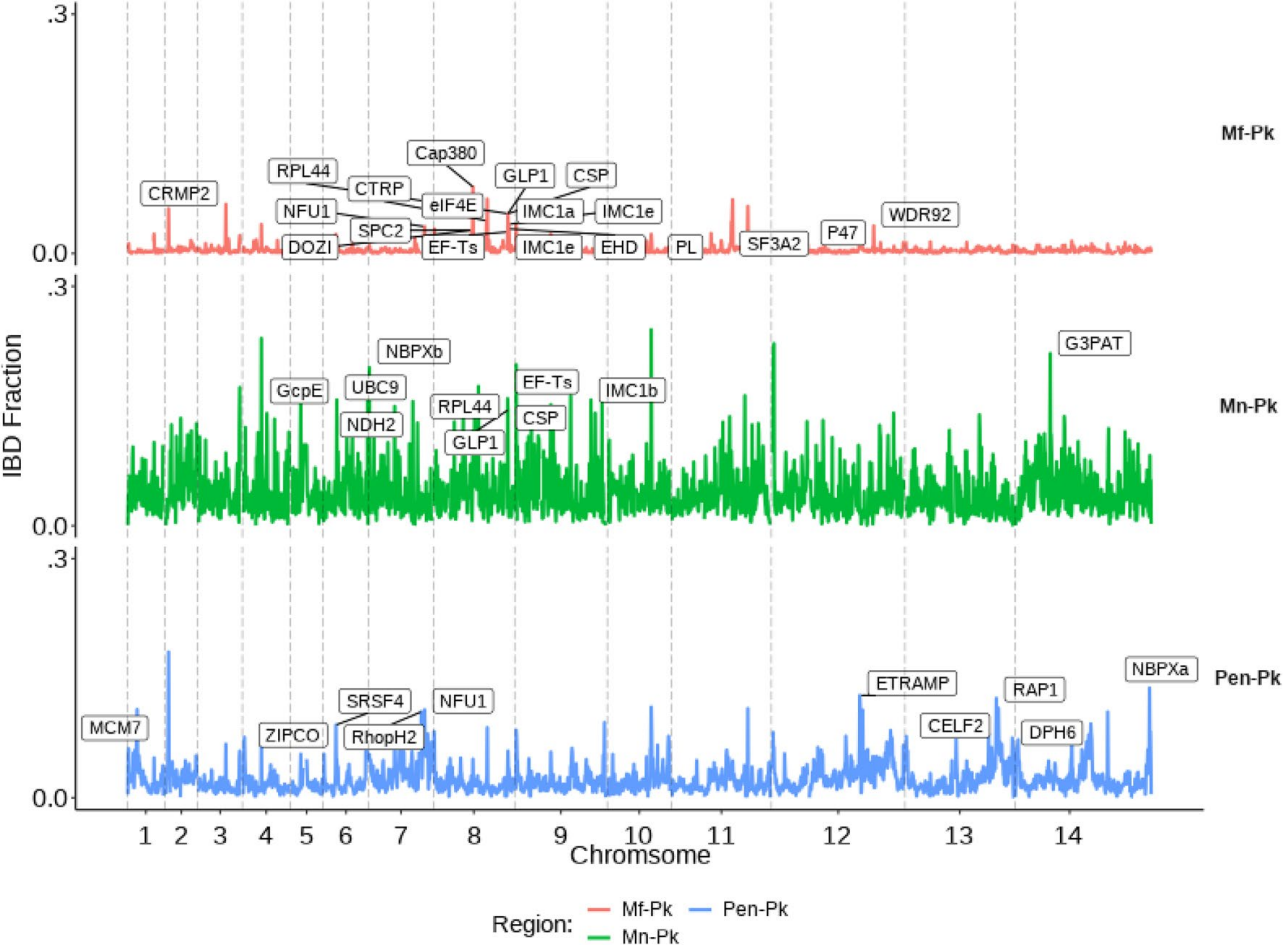
Genome-wide distribution of identity by descent (IBD) fractions across studied populations of *P. knowlesi* isolates, summarised in sliding windows of size 10kbp. Vertical dashed lines represent chromosome boundaries. Genes located in fragments with 1$\%$ of highest IBD fraction values have been marked.

Identity by descent (IBD) analysis was performed for each of the studied groups (Mf-Pk, Mn-Pk, Pen-Pk) to understand the chromosome-level structure and shared ancestry of *P. knowlesi* sub-populations. High IBD values indicate sites of common descent within a group. The fractions of pairwise IBD in the Mn-associated cluster were found to be the greatest (median: 0.087), suggestive of high levels of relatedness among isolates (S2 Figure). In contrast, the Mf-Pk group presented significantly lower pairwise fractions (median: 0.007), while values for Pen-Pk isolates were in-between (median: 0.026) (S2 Figure, S2 Table). Genome-wide IBD fractions, summarised within 10kbp sliding windows, confirmed the differences between Macaque-associated clusters, with the Mn-Pk group being more strongly related compared to the widely divergent Mf-Pk samples (Fig. 2, S2 Table). In Mf-associated groups, several fragments revealed high IBD fraction values, including a region on chromosome 8 (940–950 kbp) containing the Cap380 putative protein (PKNH_0820800), which is essential for oocyst capsule formation in *P. berghei*^16^. Another region with high IBD was in chromosome 11 (1420–1460 kbp), and contained the ATP synthase-associated protein, PIMMS43, acyl-CoA synthetase and F-box (PKNH_1130100 to PKNH_1131100). In *P. berghei*, the ATP synthase-associated (PKNH_1130200) protein is essential for parasite transformation from ookinete to further mosquito stages^17^, while PIMMS43 proteins (PKNH_1130300 and PKNH_1130900) are required for sporogonic development in the oocyst and production of sporozoites able to infect^18^. The conservation of vector-related genes like PIMMS43 has been recently observed in a rodent malaria parasite^19^. Furthermore, in the cluster, we identified other putative proteins that play an essential role in mosquito-related stages of the parasite cycle including CRMP2, CTRP, IMC1a, and IMC1e^20^. The Mn-associated cluster presents a highly similar structure across the genome, and multiple peaks associated with high IBD values were found, including a region on chromosome 7 (610–620 kbp; PKNH_0713000 to PKNH_0713300) containing putative proteins NDH2 and UBC9. The knock-out of NDH2 in *P. berghei* leads to the inability to develop into mature oocysts in the mosquito midgut^21^. While the UBC9 locus is involved in the regulation of the erythrocyte developmental cycle^22^, with its high expression in this stage confirmed in single cell data analysed^23^. A high peak was observed on chromosome 9 (1810–1820 kbp), including putative IMC1b (PKNH_0939700) found to be an ortholog of a *P. berghei* protein responsible for the mechanical strength and motility of parasite ookinetes^24^. Interestingly, four regions appeared as important in both Borneo clusters (S2 Table). One of the fragments on chromosome 8 (1770–1780 kbp) encompassing five genes (PKNH_0838400 to PKNH_0838800), included the circumsporozoite (CSP) protein (PKNH_0838500), which codes the surface antigen of sporozoites and is expressed in high levels in sporozoites that reached the mosquito salivary gland^25^. The putative 60S ribosomal protein L44 (RPL44 - PKNH_0838700) was visibly expressed in human blood stages in the analysis of single cell data conducted in *P. knowlesi*^23^. In addition, one of the conserved fragments included the NBPXb gene, which is known to be required for the invasion of host erythrocytes^26^ and genetically similar across clusters^12^. Overall, analysis of gene expression patterns for the Borneo group revealed that some of the loci identified in the IBD analysis (69/99) were linked to the blood stages of the *P. knowlesi* life cycle (rings, trophozoite and schizont) (S2 Table). Interestingly, the IBD analysis for the Peninsular cluster (Pen-Pk) demonstrated mostly high peaks containing genes previously linked to vertebrate parasite stages in Plasmodium species, including putative ZIPKO, RhopH2, ETRAMP, RAP1 and NBPXa proteins (PKNH_0606600, PKNH_0727900, PKNH_1246400, PKNH_1347900 and PKNH_1472300). In *P. berghei*, the ZIPKO protein is crucial for parasite development inside hepatocytes^27^. Furthermore, in *P. falciparum* RAP1 is required for erythrocyte invasion^28^, while RhopH2 plays a role in the formation of new permeability pathways in infected erythrocytes^29^. The ETRAMP family in *P. falciparum* has been linked to parasite development from ring to trophozoite stages, however expression patterns were not confirmed in the single cell data. Moreover, the NBPXa in *P. knowlesi* is known to be an essential mediator in human erythrocyte invasion^26^ and its strong genetic divergence has been reported^12^. In addition, some reported genes displayed signs of high expression in single cell analysis, with exceptionally significant expression found in PKNH_0623000, PKNH_0946200 and PKNH_126300 (elongation factor 1-gamma)^23^. Overall, in general, the high IBD fragments in the Peninsular group contained genes linked with the host-related stages of the parasite cycle.

### Genetic regions underlying high divergence between Peninsular and Borneo clusters

**Figure 3:**
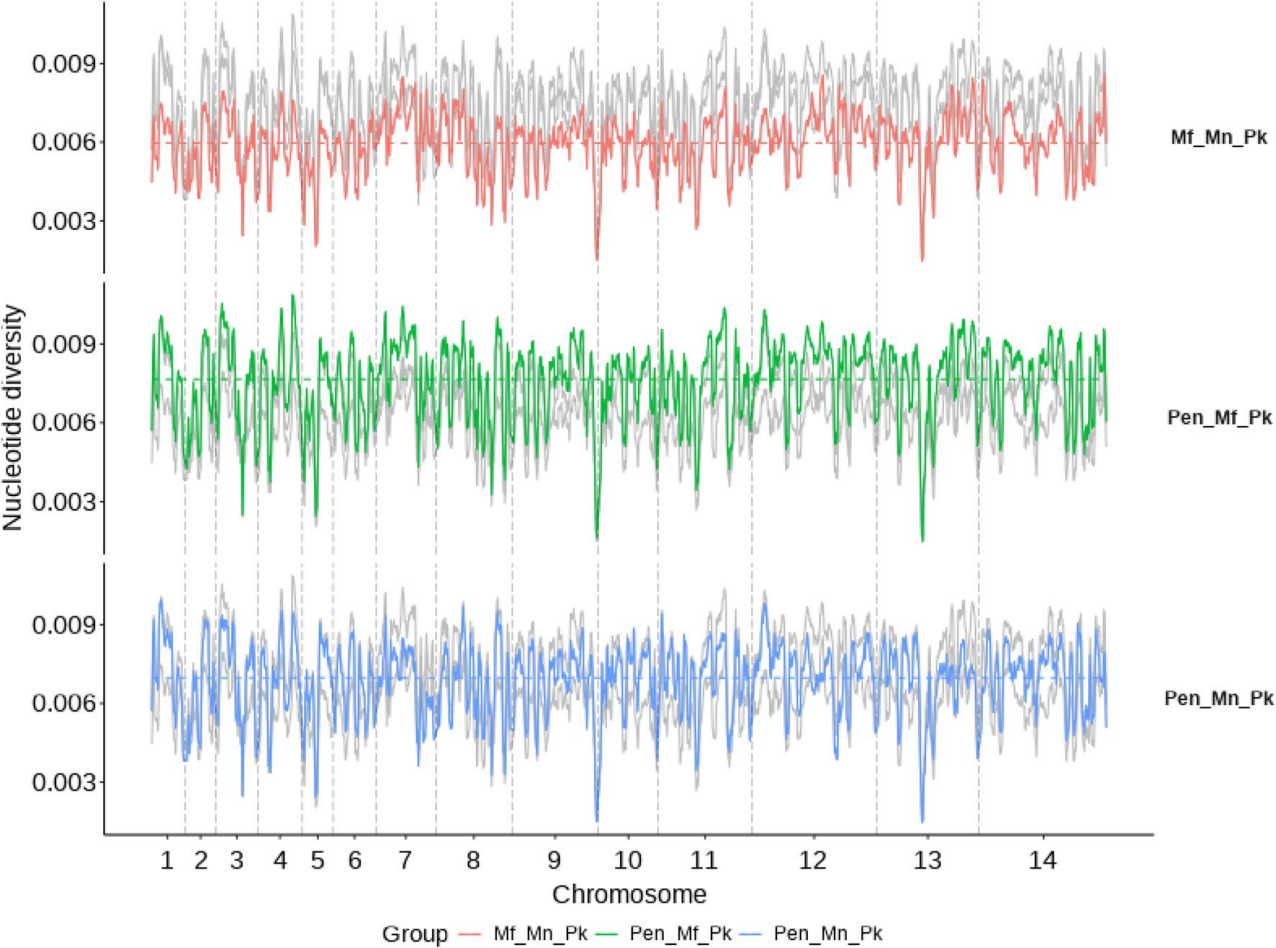
Genome-wide scan of nucleotide diversity, summarised in sliding windows of size 100 kbp, with a step size of 10 kbp. Horizontal dashed lines are the mean pairwise nucleotide diversity values for each sub-population, while vertical dashed lines represent chromosome boundaries.

A genome-wide scan of pairwise nucleotide diversity ($\pi$) was conducted among groups (Fig. 3). Joint nucleotide diversity for the Borneo isolates was the lowest (mean $\pi$: 0.0058). The Pen-Pk and Mn-Pk clusters (mean $\pi$: 0.0067) had lower values in comparison to the joint nucleotide diversity of Pen-Pk and Mf-Pk (mean: 0.0074). The lowest nucleotide diversity occurred genome-wide in the Borneo clusters (Mf-Pk and Mn-Pk), with identical fragments found on chromosomes 5, 8, 10, 11 and 13 (Fig. 3). Chromosome 8 has been linked with chromosomal-segment exchange events between Borneo populations^14^. The joint nucleotide diversity for the Peninsular and Mn-associated clusters decreased slightly in regions on chromosomes 7, 12 and 13, potentially contributing to the mosaic pattern. Joint nucleotide diversity was greatest when the Mf-Pk and Pen-Pk groups were compared, with no regions displaying significant similarities. Genes presenting low nucleotide diversity across pairwise comparisons and at the same time divergence to the remaining sub-population were found (Table 1). Unsurprisingly, most of these genes (n = 50, 63.3%) were associated with Borneo clusters and were spread along almost all chromosomes (Table 1). In contrast, the Peninsular and Mn-associated sub-populations revealed a significant number of highly similar genes (n = 27), especially on chromosome 7, which carries more than half of the loci identified. The third comparison (Pen-Pk vs. Mf-Pk) indicated less divergence between these two groups, as only 2 genes displayed significant similarity (Table 1).

**Figure 4:**
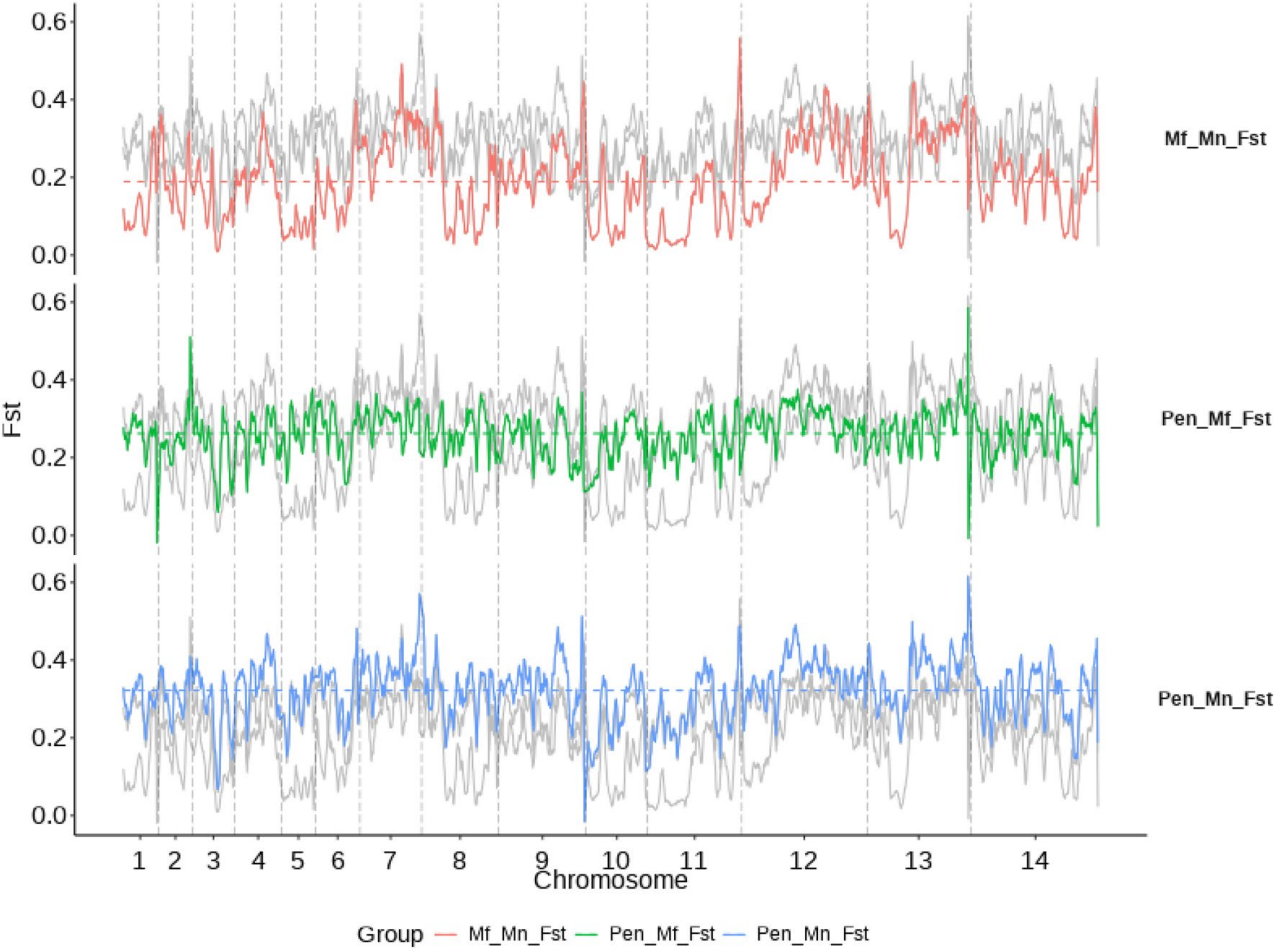
Pairwise fixation index (Fst) between sub-populations, summarised in sliding windows of size 100 kbp, with a step size of 10 kbp. Horizontal dashed lines are the mean pairwise Fst values for each sub-population, while vertical dashed lines represent chromosome boundaries.

To further investigate the genes involved in the high genetic divergence between *P. knowlesi* sub-populations, differences in allele frequencies were assessed using the fixation index (Fst). Mean genome-wide Fst values were greatest between Pen-Pk and Mn-associated clusters (mean Fst = 0.322), followed by Pen-Pk versus Mf-Pk (mean Fst = 0.266) and between Borneo groups (Mn-Pk vs. Mf-Pk; mean Fst = 0.170). The results were consistent with the nucleotide diversity analysis, where regions of low nucleotide diversity between Pen-Pk and Mn-Pk cluster on fragments of chromosome 7, 12 and 13 were highly divergent among the Borneo populations. There are clear mosaic patterns of allele frequency differences, especially visible between the Borneo populations (Fig. 4), and we focus on genes with high numbers of fixed SNPs (Fst = 1) among the comparisons (S3 Table). The high density of point mutations in *P. knowlesi* led to more than half of known genes (n = 2199) with reported fixed SNPs. For the Pen-Pk and Mn-Pk comparison, the highest number of genes (n = 1,898) with fixed SNPs were found, including ApiAP2 (PKNH_1232600) (23 SNPs), SETvs (21), CAF1 (21), RRP6 (21), RhopH2 (19), EG5 (18), SEC7 (17), RON2 (16), AP2NAG (16), MRP2 (16), RAP1 (14), SR140 (14), and SLARP (13). These loci are involved across different parasite life cycle stages. The orthologous PfSETvs is involved in controlling expression levels of *var* genes in *P. falciparum*, while RRP6 has a role in the regulation of dynamic chromatin structure^30^. By comparing the Peninsular and Mf-associated groups, 1289 genes were revealed, including EG5 (16 fixed SNPs), two putative AP2 domain transcription factors (PKNH_1016500 (13); PKNH_1232600 (14)) known to regulate parasite life cycle transition^31^, and CRMP4 (13) linked to sporozoite egress. More than 80% of genes with a fixed SNP overlapped with the previous comparison (Pen-Pk vs. Mn-Pk), confirming strong differences between Peninsular and Borneo isolates, especially in EG5, ApiAP2 (PKNH_1016500, PKNH_1232600), SETvs, and RRP6, with only one fixed SNP between Borneo groups in these loci. The lowest number of genes with fixed SNPs (686) was between the two Borneo clusters, with more than half (53.6%) found in regions on chromosomes 7, 12 and 13 (Fig. 4, S3 Table), including GDV1, LRR8, RON2, IMC1f, CRMP4, and AP2NAG2 genes. Other genes presenting notable quantities of fixed SNPs between Borneo clusters, included NBPXa (57), ApiAP2 (PKNH_1417900) (48), and the MRP2 protein (47) linked with drug resistance in other Plasmodium species. To further examine the broad function of genes with fixed SNPs, an analysis of Gene Ontology (GO) enrichment (molecular function, biological processes, cellular component) was performed (S4 Table). This analysis revealed the highest number of associated GO sub-groups (62) was linked to the Pen-Pk and Mn-Pk comparison, with the broader molecular function class being the most common (34/62). Similar results were found for Pen-Pk and Mf-Pk, but the Borneo sup-populations differ predominantly with many fixed SNPS in genes linked to broader biological processes (S4 Table).

**Table 1:** Genes presenting low nucleotide diversity ($\pi$) between two *P. knowlesi* sub-populations with significant divergence to the third cluster categorised by expression level reported in human-related stages of *P. knowlesi* using single cell experiment data^23^.

Comparison	High expression - ring [PKNH_]	High expression - Schizont [PKNH_]	High expression - Trophozoite [PKNH_]	Low expression in host-related stages [PKNH_]
Mn-Pk versus Mf-Pk	0604600, 0609000, 0612800, **0734800**, **0836600**, 0900700, 0934600 (SRP72), 1264600, 1464500	0101800, 0111000 (DegP), 0515200, **0819900 (H2A.Z)**, 1347900 (RAP1), 1464500	0515200, 0604600, 0609000, **0819900 (H2A.Z)**, 0900700, 0926200, 1264600, 1464500	0103400, 0111600, 0404800, 0415700, 0421500, 0501000, **0513200 (RRF2)**, 0713800 (GLTP), **0815200**, **0820100 (SPC2)**, **0821300**, **0824300 (EG5)**, **0829100**, 0904500, 0911900, 0918600 (HSBP), 1026200, 1118800, 1130900, 1132000, 1135000, 1136000, **1144800**, 1212200 (IF1), 1217900, 1253500, 1263400, 1303000, 1307200, 1314600, 1337700, 1350600, 1444200 (SF3B6), 1446400
Pen-Pk versus Mf-Pk	0100700	0100700	0100700	0602300
Pen-Pk versus Mn-Pk	**0734500**	**0734600**, 0812000, 1402300 (GAP)	0714500 (UFD1), **0734500**, **0734600**, 1234000	0100500, 0215700, 0713100 (UBC9), 0713500, 0722800, 0727700, 0727800, 0728400, 0728700 (LRR8), 0731600, **0733600**, **0733900 (VPS33)**, **0734200**, 0811800, 1136300, 1234900, 1240100, **1249600**, 1354000, 1434300

Bolded genes have been observed in exchange events.

### Characterisation of genetic exchange events among Borneo isolates

Using an integrated approach involving Uniform Manifold Approximation and Projection (UMAP) clustering, neighbour-joining tree and nucleotide diversity estimation methods (see Methods), strong signals of introgression (genetic exchange events) were identified among Borneo isolates in chromosomes 5, 8, and 11 (S3 Figure). For the chromosome 5 (600–700 kbp) region, some Mf-associated isolates were found within the Mn-Pk group (S3 Figure  A). The related chromosome 5 genes included putative RIO2 (PKNH_0512800), ACP2 (PKNH_0513000), ApiAP2 (PKNH_0513100) and RRF2 (PKNH_0513200). Analysis conducted on multiple loci throughout chromosome 8 (600–800 kbp, 850–1400 kbp, 1500–1700 kbp) confirmed the clustering of some Mf-Pk isolates within the Mn-associated group (S3 Figure  B, C, D). The matching sequence segments were observed in subgroups of Betong and Sarikei Mf-Pk isolates, consistent with previously observed introgression events of large chromosomal regions between Mf-Pk and Mn-Pk^14^. The chromosome 8 regions included genes linked with sexual development (e.g. HAP2 - PKNH_0814100, DOZI - PKNH_0820000) and parasite development in mosquitoes (e.g. oocyst Cap380 - PKNH_0820800, CTRP - PKNH_0826900). Host associated genes with evidence of essential asexual development in *P. falciparum*, included HSP60 (PKNH_0815700), GSK3 (PKNH_0829800), H2A.Z (PKNH_0819900), ABCI3 (PKNH_0821500) and ARK2 (PKNH_0833500). Numerous genetic exchange events were identified throughout chromosome 11 (100–300 kbp, 500–700 kbp, 1800–1900 kbp, 2000–2200 kbp) (S3 Figure  E, F, G, H), mostly involving separation of various Betong and Sarikei Mf-Pk isolates. One of the introgression events (500–700 kbp) contained Mf-Pk samples isolated from Kapit. In addition, singular Mn-Pk isolates were detected among the Mf-Pk sub-population, confirming that genetic exchange events occur in the opposite direction, but with lower frequency and on a smaller scale. Most chromosomal events involved genes linked to vector related parasite stages, including DHHC2 (PKNH_1140400) and PSOP2 (PKNH_1103400) proteins, essential for ookinete morphogenesis, as well as SAS6 (PKNH_1142700) found to be a key protein in male gametogenesis in *P. berghei*. Moreover, close genetic similarity and a lack of separation between Mn-Pk and Mf-Pk clusters demonstrated in those loci accounts for the mosaic structure of the sub-populations. All the selected loci had clear re-branching of the Mn-Pk cluster from the more diverged Mf-associated group.

**Figure 5:**
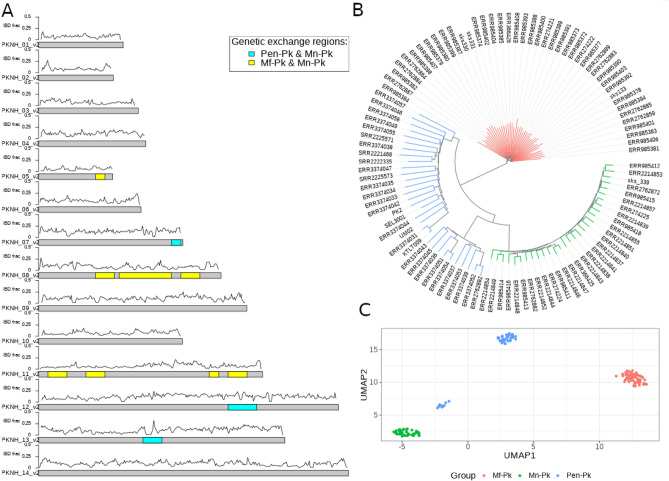
**L**oci and evidence of exchange events between Mn-Pk and Pen-Pk clusters. (**A**) IBD fraction for the Mn-Pk and Pen-Pk isolates, regions with evidence of exchanges are coloured; (**B**) Neighbour-joining tree for chromosome 7, 1400–1500 kbp; (**C**) UMAP plot for chromosome 7, 1400–1500 kbp.

Using the same analytical approach as above, three regions were identified as highly similar between Pen-Pk and Mn-Pk groups, with evidence of genetic exchange events occurring on chromosome 7 (1400–1500 kbp), 12 (2000–2300 kbp) and 13 (1100–1300 kbp) (Fig. 5, S4 Figure). These regions were identified in the IBD analysis for combined Pen-Pk and Mn-Pk sub-populations, as well as in the nucleotide diversity analysis (Table  1). Almost all isolates involved in exchanges were sourced from the Peninsular region of Kuala Lipis and Sungai Siput, with Taiping and Johor locations contributing to singular events. The fragment on chromosome 7 contains multiple genes, including putative PKAc (PKNH_0733500), GDV1 (PKNH_0734100) and ETRAMP (PKNH_0734700), and are predominantly linked to the host related stages of the Plasmodium cycle. The *P. falciparum* PKAc gene is linked to asexual stage growth, the gametocyte development protein (PfGDV1) is critical for the early sexual differentiation, and ETRAMP gene is fundamental for transformation from rings to trophozoites. In addition, the single cell expression data confirmed high expression of the GDV1 and ETRAMP genes, as well as some of the neighbouring genes of unknown function (PKNH_0734500 and PKNH_0734600)^23^. The largest fragment involving genetic exchange was on chromosome 12 (length 300kbp), and contains more than 50 genes, including the CRMP4 (PKNH_1245700), ETRAMP (PKNH_1246400), UTP15 (PKNH_1248800), ABCB6 (PKNH_1248900), IMC1f (PKNH_1249300) and multiple PHIST proteins (PKNH_1247500, PKNH_1247600 and PKNH_1247700). The identified region on chromosome 13 was mostly characterised with Plasmodium exported proteins of unknown function. However, two PHIST genes (PKNH_1324600, PKNH_1326000) and the inner membrane complex suture component (ISC3 - PKNH_1326900) were also identified.

### Genetic diversity in invasion genes

The first evidence that the *P. knowlesi* parasite comprises distinct sub-populations was revealed in the analysis of erythrocyte invasion genes^32^, and later confirmed and extended with the knowledge of inter-group exchange events^12^. We used the neighbour-joining tree and nucleotide diversity approach to characterise the structure of the two reticulocyte binding like (RBL) and three Duffy binding protein like (DBP) genes associated with erythrocyte invasion (S5 Figure). The DBP$\alpha$ gene was the only locus with clear partitioning into three sub-populations coinciding with a whole genome classification (S5 Figure  A). For both DBP$\beta$ and DBP$\gamma$ (S5 Figure  B, C), the clustering pattern matches the overall genetic structure, but some of the Pen-Pk isolates show evidence of genetic exchange with Mf-Pk and Mn-Pk clusters. A Pen-Pk isolate from Taiping (ERR3374057) aligned more closely with the Mf-associated cluster using the DBP$\beta$ locus. Moreover, in the same locus, a subset of Pen-Pk samples from Singai Siput and Kuala Lipis present with exchange events linking them to the Mn-associated group. These exchanges show that recombination events of the Peninsular group can be linked to both Borneo clusters in the same loci, potentially contributing to the high diversity of the Pen-Pk isolates. The evidence of exchange events among subsets of Pen-Pk isolates with the Mf-associated cluster were also observed in the DBP$\gamma$ locus. This exchange contains four samples, including a historical laboratory strain (SRR2225573, The Philippines), a newly derived clone from *M. fascicularis* blood culture (UM02), and 2 field isolates (ERR3374057, Taiping; ERR3374049, Kuala Lipis), with one linked to the Mf-Pk cluster involving the DBP$\beta$ region. Existence of the recombination events within laboratory maintained isolates, and their significant branch length on the tree, confirmed that such events are non-recent. For the NBPX$\beta$ locus (S5 Figure  D, E), subdivision of the Mf-Pk group was observed. However, Mn- and Pen-associated sub-populations were indistinguishable, with a subset of seven Pen-Pk isolates collected from various locations (Kuala Lipis, Taiping and Sungai Siput) being affiliated to the Mn-associated cluster. A similar genetic exchange event was confirmed within the NBPX$\alpha$ locus (S5 Figure  D), where a subgroup of ten Pen-Pk isolates (mostly from Kuala Lipis and Sungai Siput) aligned more closely with the Mn-associated sub-population. Once again, the significant length of most of the divergent branches is indicative of the genetic exchange event being non-recent.

### Regions under selection in *P. knowlesi* sub-populations

A genome-wide scan for genes under recent positive selection was performed for each sub-population using the integrated haplotype score (iHS) approach. Results for the Mf-associated group revealed numerous fragments spreading across all chromosomes (S6 Figure  A). Moreover, fragments on chromosome 8 (0.6–0.66 Mbp, 0.9–0.95 Mbp, 1.02–1.05 Mbp and 1.68–1.71 Mbp) and 11 (2.07–2.1 Mbp) were found to be overlapping with the previously shown differentiation of the Betong and Sarikei sub-population^14^. In contrast, the Mn-Pk group shows little evidence of extended haplotypes affirming its low genetic diversity (S6 Figure  B). Selective sweeps in the Peninsular cluster were identified on chromosomes 5 and 6 (S6 Figure  C), which include inner membrane complex (IMC1m, PKNH_0613600) and merozoite TRAP-like (MTRAP, PKNH_0613400) proteins.

Genome-wide scans for positive selection that compared between sub-populations revealed little evidence of selective sweeps for Pen-Pk and Mn-Pk clusters (S6 Figure  D, E, F). However, multiple fragments were identified between the Peninsular and Mf- associated groups, including regions on chromosomes 4 (0.82–0.85 Mbp; e.g., LRR5 gene, PKNH_0419000), 7 (0.58–0.61 Mbp), 11 (2.17–2.20 Mbp; e.g., SIP2 gene, PKNH_1146400), 12 (2.45–2.48 Mbp; e.g., VPS13 (PKNH_1264700)), and 13 (0.85–0.88 Mbp; e.g., IF3a (PKNH_1319200)). Almost all of the fragments enclose putative PIR or SICAvar genes. A comparison of the Borneo associated clusters revealed fragments with selection signals on chromosomes 2 (0.37–0.40 Mbp), 5 (0.30–0.33 Mbp), 7 (1.08–1.11 Mbp) and 14 (1.18–1.21 Mbp). The NPT1 protein (PKNH_0208700) was identified and is known to be crucial for early stage of sexual development in *P. berghei*.

## Discussion

Despite the widespread occurrence of *P. knowlesi* parasites in Southeast Asia, there are many gaps in the understanding of its evolutionary dynamics, geographical distribution and transmission. Whilst the genetic adaptation of the parasite to the macaque hosts on Borneo Island and geographical differences in comparison to Peninsular Malaysia have been described^12^, the mosaic genome-wide structure occurring between those sub-populations is not well understood. Our study performed genome-wide sequence analyses of the largest collection of *P. knowlesi* isolates yet assembled, and revealed evidence of genetic exchange events between highly divergent Peninsular Malaysia (Pen-Pk) and one of the Borneo groups associated with *M. nemestrina* (Mn-Pk), as well as between Borneo sub-populations. Although, our work extended important whole-genome characterisation of understudied regions of Malaysia and beyond, wider sampling and larger studies are required. Further, inclusion of laboratory isolates cultured in *M. fascicularis* blood provided evidence of non-recent genetic exchange in erythrocyte invasion related genes, whose functional effects can be investigated using *in vitro* models^26^. Nonetheless, inclusion of more isolates derived from wild macaques would be necessary for a more in depth understanding of evolutionary changes of the *Plasmodium* species.

Exploration of *P. knowlesi* population structure across Malaysia revealed three predominant clusters, consistent with previous findings^13,14^. All newly sequenced isolates aligned within those sub-populations, and the majority had mono infections and single source population ancestry. This observation would have been expected for the laboratory strains, which have undergone *in vitro* culture. However, three samples isolated from Sarikei had inferred mixed ancestry from both Borneo clusters, consistent with multiple genotypes of *P. knowlesi* in human infections and the circulation of underlying sub-populations in that geographical region^15^. A *P. knowlesi* and *P. vivax* co-infection was found, and infections with more than one *Plasmodium* species have been previously observed^33^.

Among all three *P. knowlesi* sub-populations, the Mn-associated group is known to be most genetically conserved, especially in highly divergent regions shown in other sub-populations^34^. It has been suggested that this observation is a result of the Mn-Pk group being an initial bottleneck in the formation of sub-populations^34^. Whereas, the *M. fascicularis* specific (Mf-Pk) and Pen-Pk groups present as genetically divergent throughout the genome^35^. IBD analysis supported these insights, and revealed regions of common ancestry within and between Borneo populations, appearing to be linked with some known vector-related genes like Cap380 and CSP. Multiple regions were found to display high similarity within both Borneo-associated sub-populations, with various fragments on chromosome 8 previously described to have genetic exchange events between the Mf- and Mn-associated genotypes^14^. The identified vector-related genes among the Borneo groups may be linked with the specific ecology of members of the Leucosphyrus Complex *Anopheles* (*An. latens* and *An. balabacensis*) spread across Malaysia, Indonesia, Singapore, Brunei and part of The Philippines^36^. Nonetheless, differences in the habitat zone of *M. fascicularis*, being a canopy roosting macaque while *M. nemestrina* is mainly terrestrial, may expose the animals to distinct sub-populations within the Leucosphyrus Group that are closely related but distinct from mosquitoes in other zones. In contrast, peninsular Malaysia and mainland Southeast Asian countries are associated with *Anopheles* species of the Dirus Complex (e.g. *An. cracens*) as the primary vectors of *P. knowlesi*^36^. Our observed levels of IBD within the Peninsular cluster were found to be remarkably high in loci predominantly related to host-parasite stages. Dissimilarities among sites of common descent between sub-populations could be caused by the geographical separation of the Borneo Island from Peninsular Malaysia.

The mosaic pattern and population differentiation across the Borneo-associated nuclear genome have been highlighted in previous population-based studies, leading to the detection of chromosomal-segment exchange events throughout chromosome 8^14^. Our analysis scanned genome-wide for introgression events across all three sub-populations, extending our knowledge of new exchange events between the Borneo sub-populations located on chromosomes 5 and 11. We identified new regions with evidence of genetic exchanges between previously understudied Mn-Pk and Pen-Pk associated clusters. Introgression events occur on chromosomes 7, 12 and 13, with identified loci possessing patterns of high divergence between the Borneo-associated genotypes, potentially explaining some of the factors driving mosaicism among the sub-populations. These results are consistent with a previous candidate gene analysis^12^ and microsatellite analyses that identified traces of Borneo-associated clusters in genomic regions of Peninsular Malaysia^37^. The differences in branch length within and between groups in the neighbour-joining tree analysis suggest that the newly detected exchange events among Mn-Pk and Pen-Pk isolates are potentially caused by historic macaque crossings. Given the likely dates of these past events, the introgression is unlikely to have been facilitated by human host transitions, although further analyses are necessary to properly answer this question. The Pen-Pk genomic regions with exchange events were enriched with host-related genes, whereas Borneo-associated exchange events were primarily connected to mosquito-related stages of the parasite cycle. A potential alternative explanation for the extended regions of clusterisation outside of groups could be incomplete lineage sorting, where such changes have been observed with recent divergence among *P. vivax* species^38^. An analysis of invasion linked loci found that RBL/DBP (DBP $\alpha$, $\beta$ and $\gamma$, NBP X$\alpha$ and X$\beta$) genes are highly divergent and mostly coinciding with a whole genome classification. Between Pen-Pk and Mn-Pk associated sub-populations, exchange events were found in all studied invasion genes except DBP$\alpha$. For the DBP$\beta$ and DBP$\gamma$ genes, individual Pen-Pk isolates were strongly differentiated from their original cluster. Additionally, the DBP$\beta$ locus has singular samples presenting strong affiliation to the Mf-associated cluster, highlighting that multiple introgression events can be found at the same loci. Exchange events revealed on the DBP$\gamma$ gene include a laboratory cultured Philippine strain and a new isolate collected from *M. fascicularis* red blood cell culture. This observation suggests that the genetic exchanges are not recent, especially because of the significant length of the neighbour-joining tree branches and isolation of the laboratory sample in year 1960. The NBPX$\alpha$ and NBPX$\beta$ genes present introgression events where some Pen-Pk isolates cluster with the Mn-Pk associated sub-population. Almost all of the invasion genes show genetic exchange events between Pen-Pk and Mn-Pk associated clusters, highlighting the potential ancestral events between those clusters related to the adaptation of the parasite to different simian hosts. Exchange events in those genes can strongly impact on invasion mechanisms, which can be explored using experimental models^26^.

Our work has provided new insights into *P. knowlesi* evolution, highlighting the genetic exchanges as well as regions of identity among known sub-populations. The *P. knowlesi* data demonstrate significant divergence caused by the geographical separation in the Peninsular cluster and non-human host specification in Borneo groups, whilst, still displaying the ability to recombine when in contact^11^. The fragments of recombination can have an important impact on patterns of diversity among sub-populations and create the observed mosaicism. Our analyses contribute to the investigation of the genetic structure of the parasite by providing evidence of new introgression events. Genetic exchange events found between Pen-Pk and Mn-Pk associated clusters almost entirely overlay with long regions of high divergence shown in Borneo sub-populations, and are non-recent, suggesting a long-term process contributing to the population structure and mosaic patterns of *P. knowlesi* we observe today. In lieu of larger and geographically diverse studies within the wider region, our work has generated new hypotheses as to the historical shape of population movement for both parasite and host, which can be tested in geographically broader sample sets as they become available.

## Methods

### Isolates and sequence data

A total of 151 *P. knowlesi* isolates with WGS were analysed, including: (i) publicly available data (n = 126)^12,13,15,34,35^; (ii) isolates sourced from Malaysia (n = 15; Peninsular 4, Borneo 11), provided by the University Malaya Medical Centre (spanning July 2008 to December 2014); (iii) isolates from returning travellers to the UK from Malaysia (n = 1) and Indonesia (n = 1); (iv) laboratory strains (n = 8), including two samples isolated from *in vitro* blood cultures of *M. fascicularis* (ERR9751937 and ERR9751954) and six samples from *in vitro* human red blood cell cultures (PkA1-H.1). DNA was extracted from clinical samples using the DNeasy Blood & Tissue Qiagen kit, and underwent parasite selective whole genome amplification, as described previously^12^. Sequencing of the DNA from the newly generated isolates (n = 25) was performed on an Illumina HiSeq 4000 platform by The Applied Genome Centre, LSHTM. All WGS data was screened using Centrifuge software to ensure a significant abundance of reads derived from *P. knowlesi* (abundance higher than 0.35), and to confirm host species (*H. sapiens*, *M. fascicularis* or *M. nemestrina*). Ethical approval in written and verbal form was provided by the University of Malaya Medical Centre Medical Ethics Committee (Ref. No: 817.18). Informed consent was obtained for study participation in both Sabah and Kuala Lumpur sites. The UK National Research Ethics Service (Ref: 18/LO/0738) and LSHTM Research Ethics Committee (Ref: 14710) provided approval for the project “Drug susceptibility and genetic diversity of imported malaria parasites from UK travellers”. New data provided in the study can be found on ENA (PRJEB52783). Details of the WGS data, including ENA accession numbers, are provided ( S1 Table).

### Bioinformatic analysis

All raw sequencing data was filtered using trimmomatic software (v0.39; parameters: LEADING:3 TRAILING:3 SLIDINGWINDOW:4:20 MINLEN:36)^39^. Filtered reads were then mapped to the *P. knowlesi* A1-H.1 reference genome^40^ using BWA-MEM alignment (v0.7.17) software^41^. SNPs and insertions/deletions (indels) were found using GATK’s Base Quality Score Recalibration and HaplotypeCaller (v4.1.4.1), and validation was performed using the ValidateVariants function with default settings^42^. Variants with a Variant Quality Score (VQSLOD) in excess of zero and low levels of missing genotypes (<20%) were retained. Variants occurring in subtelomeric regions or SICAvar genes were excluded. The summary statistics for all analysed samples are provided (S1 Table). The final dataset consists of 151 isolates representing 3 clusters (Cluster 1 - Mf-Pk, n = 60; Cluster 2 - Mn-Pk, n = 41; Cluster 3 - Pen-Pk, n = 50), with 1,883,700 high quality SNPs. Variants were annotated using snpEff (v4.1) software^43^. The multiplicity of infection (MOI) was estimated from biallelic variants using estMOI software^44^ and the Fws score, calculated using the mixmoi package (github.com/bahlolab/moimix) for each of the known clusters (Mn-Pk, Mf-Pk and Pen-Pk). Isolates with Fws values $\ge$ 0.95 were taken as infections with a predominant singular genotype, whereas samples below the threshold were assumed to be mixed infections as previously shown^10^.

### Population genetic analysis

Population structure was explored by performing a PCA and constructing a neighbour-joining tree using the R ape package. PCA was performed on the 151 available isolates using pairwise Manhattan distances based on biallelic SNPs. The ADMIXTURE (v1.3.0) software was applied to estimate individual ancestry^45^, where the number of sub-populations was determined by cross-validation error. The ADMIXTURE analysis was filtered for linkage disequilibrium (LD), where SNPs were identified for pruning using Plink software (with settings: – indep-pairwise 50 10 0.1). Samples with evidence of the multiplicity of infection (Fws < 0.95), low coverage (<five-fold) or identified as highly similar (< 50,000 sites difference) were excluded from any further analyses, to ensure a more robust population genomics analysis. The resulting dataset of 104 high-quality isolates (Mf-Pk, n = 43; Mn-Pk, n = 30; Pen-Pk, n = 31) was used for population genetic analysis. The IBD analysis was performed on biallelic SNPs with a minor allele frequency (MAF) of >5%. This analysis was applied pairwise between each group using the package hmmIBD^46^, and involved sliding windows of size 10kbp. The proportion of pairwise comparisons for isolates presenting evidence of IBD, corrected for fragment length, was plotted by genome location. The top 1% of hits were considered significant.

To measure the amount of polymorphism within each cluster, the average pairwise nucleotide diversity ($\pi$) was estimated genome-wide using the R Pegas package with sliding windows (100 kbp window size, 10 kbp step size). Pairwise nucleotide diversity between groups was calculated for each gene, and the results used to report loci presenting low nucleotide diversity between two clusters, but highly separated from the remaining one. The fixation index (Fst) metric was calculated using VCFtools software on SNPs with a MAF > 5%. A genome-wide scan of the Fst results was performed using sliding windows (100 kbp window size, 10 kbp step size). Genes containing fixed SNPs (Fst = 1) in the pairwise sub-population comparisons were identified, and a gene ontology (GO) term analysis was performed using previously described methods^15^. Gene function across Plasmodium species was established using the PlasmoDB database (plasmodb.org).

All three groups (Mf-Pk, Mn-Pk, Pen-Pk) were screened for recent positive selection using the R rehh package^47^, applied to SNPs with MAF > 5%. Both the within-population integrated haplotype score (iHS) and between-population Rsb score for identification of selection^48^ were calculated. Critical regions were identified using 100 kbp sliding windows, which included at least 3 SNPs with a *p*-value $< 1 \times 10^{-4}$ for iHS and *p*-value $< 1 \times 10^{-5}$ for Rsb^10^. The identification of potential introgression regions was based initially on genome-wide pairwise nucleotide diversity ($\pi$), calculated within sliding windows (window size 100 kbp, step size 50kbp). Regions presenting a low level of diversity among pairwise sub-population comparisons ($\pi < 0.005$) were further investigated using the Fst metric applied within the sliding windows to confirm divergence from the remaining sub-population. To further support these exchange events, the population structure was assessed using the UMAP algorithm applied to 100 kbp windows encompassing the putative region. Fragments displaying differences from whole genome-based clustering, supported by changes in nucleotide diversity and Fst divergence analysis, were further confirmed using a neighbour-joining tree, constructed using the R ape package. The usage of the UMAP algorithm has previously separated Plasmodium species^49^. Single-cell expression data was used to link *P. knowlesi* gene findings to parasite life cycle stages^23^. The mean expression levels were calculated for each gene by stage, and loci revealed in our analysis were checked against the list of the top 20% of (highly) expressed genes^23^.

## Supplementary Information

Supplementary Information.

## Data Availability

Previously published WGS data can be found on the European Nucleotide Archive (ENA) using the Run accession codes in S1 Table. The newly generated data can be found in the ENA study accession number PRJEB52783.
