# Supplementary material for: Population genetic analysis of Plasmodium knowlesi reveals differential selection and exchange events between Borneo and Peninsular sub-populations
Source: Sci Rep. 2023 Feb 7;13:2142. doi: 10.1038/s41598-023-29368-4 (PMC9905552; doi:10.1038/s41598-023-29368-4)
Supplement: Supplementary file 1 — Supplementary Information. [file 41598_2023_29368_MOESM1_ESM.pdf]

Supplementary Materials: Population genetic analysis of *Plasmodium knowlesi* reveals differential selection and exchange events between Borneo and Peninsular sub-populations

Supplementary tables

S1 Table. Metadata for the 151 *P. knowlesi* isolates

Sample	Location	*P knowlesi* abundance	Coverage 5X	Average 5X	Reads aligned to *P knowlesi* reference (%)	Fws	Cluster	Population genetics included
ERR9751930	Lab	0.998	0.888	14.3	75	1	Pen-Pk	-
ERR9751931	Lab	1	0.911	13.7	97.28	1	Pen-Pk	-
ERR2214837 [1]	Kapit	0.993	0.933	45.1	97.65	0.998	Mn-Pk	YES
ERR2214838 [1]	Kapit	0.988	0.913	31.9	89.86	0.996	Mn-Pk	YES
ERR2214839 [1]	Kapit	0.975	0.876	20.9	73.87	0.999	Mn-Pk	YES
ERR2214840 [1]	Kapit	0.981	0.933	46	74.22	0.999	Mn-Pk	YES
ERR2214841 [1]	Kapit	0.964	0.923	36.7	54.6	0.997	Mn-Pk	YES
ERR2214842 [1]	Kapit	0.991	0.939	45.3	94.5	0.738	Mn-Pk	-
ERR2214843 [1]	Kapit	0.957	0.905	27.3	54.66	0.999	Mn-Pk	YES
ERR2214844 [1]	Kapit	0.994	0.92	34.8	98.16	0.999	Mn-Pk	YES
ERR2214845 [1]	Kapit	0.997	0.94	52.4	94.69	0.935	Mn-Pk	-
ERR2214846 [1]	Kapit	0.999	0.934	38.7	98.53	0.999	Mn-Pk	YES
ERR2214847 [1]	Kapit	0.986	0.931	43.4	80.74	0.999	Mn-Pk	YES
ERR2214848 [1]	Kapit	0.986	0.921	35.1	97.74	0.998	Mn-Pk	YES
ERR2214849 [1]	Kapit	0.993	0.929	42.9	96.96	0.999	Mn-Pk	YES
ERR2214850 [1]	Kapit	0.989	0.951	56	87.5	0.664	Mn-Pk	-
ERR2214851 [1]	Kapit	0.993	0.932	36.1	93.37	0.997	Mn-Pk	YES
ERR2214852 [1]	Kapit	0.993	0.882	20.6	97.95	0.998	Mn-Pk	YES
ERR2214853 [1]	Kapit	0.992	0.914	30.4	97.36	0.998	Mn-Pk	YES
ERR2214854 [1]	Kapit	0.999	0.94	49.7	98.53	0.998	Mn-Pk	YES
ERR2214855 [1]	Kapit	0.993	0.891	23.7	95.82	0.997	Mn-Pk	YES
ERR2214856 [1]	Kapit	0.997	0.942	35.8	98.48	0.612	Mn-Pk	-
ERR2214857 [1]	Kapit	0.994	0.881	21.3	95.85	0.975	Mn-Pk	YES
ERR274221 [2]	Sarikei	0.996	0.966	156.9	97.64	0.977	Mf-Pk	YES
ERR274222 [2]	Sarikei	0.996	0.965	202.1	98.42	0.999	Mf-Pk	YES
ERR274224 [2]	Sarikei	0.997	0.959	146.3	97.65	0.997	Mn-Pk	YES
ERR274225 [2]	Sarikei	0.997	0.962	181	97.67	0.998	Mn-Pk	YES
ERR3374031 [3]	Gua Musang	0.999	0.941	41.9	98.72	0.999	Pen-Pk	YES
ERR3374032 [3]	Gua Musang	0.997	0.923	36.6	86.07	0.999	Pen-Pk	-
ERR3374033 [3]	Gua Musang	0.997	0.952	65.9	98.46	0.999	Pen-Pk	YES
ERR3374034 [3]	Gua Musang	0.999	0.951	59	97.38	0.998	Pen-Pk	YES
ERR3374035 [3]	Kuala Lipis	0.997	0.937	36.7	96.62	0.999	Pen-Pk	YES
ERR3374036 [3]	Kuala Lipis	0.998	0.927	33.2	98.35	0.999	Pen-Pk	YES
ERR3374037 [3]	Kuala Lipis	0.998	0.932	36	97.77	0.999	Pen-Pk	YES
ERR3374038 [3]	Kuala Lipis	0.999	0.942	42.5	98.6	0.998	Pen-Pk	YES
ERR3374039 [3]	Kuala Lipis	0.998	0.951	81.2	98.69	0.998	Pen-Pk	YES

ERR3374040 [3]	Kuala Lipis	0.995	0.906	28.4	95.93	0.999	Pen-Pk	-
ERR3374041 [3]	Kuala Lipis	0.998	0.954	40.9	97.74	0.618	Pen-Pk	-
ERR3374042 [3]	Kuala Lipis	0.994	0.934	37	93.99	0.999	Pen-Pk	YES
ERR3374043 [3]	Kuala Lipis	0.986	0.931	33	81.21	0.998	Pen-Pk	YES
ERR3374044 [3]	Kuala Lipis	0.993	0.943	38.7	94.26	0.999	Pen-Pk	YES
ERR3374045 [3]	Kuala Lipis	0.994	0.928	33.8	93.32	0.999	Pen-Pk	YES
ERR3374046 [3]	Kuala Lipis	0.999	0.939	35.5	98.34	0.997	Pen-Pk	YES
ERR3374047 [3]	Kuala Lipis	0.998	0.954	65.2	98.79	0.999	Pen-Pk	YES
ERR3374048 [3]	Kuala Lipis	0.999	0.952	43.4	98.53	0.846	Pen-Pk	-
ERR3374049 [3]	Kuala Lipis	0.999	0.93	29.9	98.06	0.999	Pen-Pk	YES
ERR3374050 [3]	Kuala Lipis	0.997	0.954	57.8	97.19	0.855	Pen-Pk	-
ERR3374051 [3]	Sungai Siput	0.994	0.936	40.5	57.4	0.998	Pen-Pk	YES
ERR3374052 [3]	Sungai Siput	0.991	0.947	56.6	93.31	0.999	Pen-Pk	YES
ERR3374053 [3]	Sungai Siput	0.99	0.907	23.4	80.42	0.994	Pen-Pk	YES
ERR3374054 [3]	Sungai Siput	0.999	0.92	29.7	98.22	0.999	Pen-Pk	YES
ERR3374055 [3]	Temerloh	0.995	0.899	21.6	61.12	0.998	Pen-Pk	YES
ERR3374056 [3]	Temerloh	0.998	0.948	44.6	95.11	0.886	Pen-Pk	-
ERR3374057 [3]	Taiping	0.982	0.928	49	61.94	0.998	Pen-Pk	YES
ERR3374058 [3]	Taiping	0.998	0.949	65.5	95.67	0.998	Pen-Pk	YES
ERR366425 [2]	Sarikei	0.998	0.929	31.2	97.46	0.98	Mn-Pk	YES
ERR366426 [2]	Sarikei	0.997	0.93	28.5	95.31	0.999	Mf-Pk	YES
ERR985372 [4]	Betong	0.973	0.952	65.5	64.55	0.999	Mf-Pk	YES
ERR985373 [4]	Betong	0.993	0.95	85.3	88.23	0.999	Mf-Pk	YES
ERR985374 [4]	Betong	0.994	0.963	132.5	96.49	0.999	Mf-Pk	YES
ERR985375 [4]	Betong	0.923	0.935	17.3	21.85	0.876	Mf-Pk	-
ERR985376 [4]	Betong	0.982	0.964	118.6	78.24	0.929	Mf-Pk	-
ERR985377 [4]	Betong	0.994	0.963	138.9	95.69	0.999	Mf-Pk	YES
ERR985378 [4]	Betong	0.993	0.961	122.9	95.57	0.999	Mf-Pk	YES
ERR985379 [4]	Betong	0.995	0.959	102	95.64	0.999	Mf-Pk	YES
ERR985380 [4]	Betong	0.996	0.967	167.3	97.35	0.963	Mf-Pk	YES
ERR985381 [4]	Betong	0.996	0.961	104.5	95.4	0.999	Mf-Pk	YES
ERR985382 [4]	Betong	0.993	0.951	43.6	49.33	0.994	Mf-Pk	YES
ERR985383 [4]	Betong	0.992	0.955	59.9	86.07	0.979	Mf-Pk	YES
ERR985384 [4]	Betong	0.996	0.955	42.4	89.06	0.977	Mf-Pk	YES
ERR985385 [4]	Kapit	0.966	0.962	115.7	61.23	0.999	Mf-Pk	YES
ERR985386 [4]	Kapit	0.858	0.945	23.5	20.82	0.617	Mf-Pk	-
ERR985387 [4]	Kapit	0.99	0.961	136	91.71	0.979	Mf-Pk	YES
ERR985388 [4]	Kapit	0.992	0.963	200.3	92.19	0.998	Mf-Pk	YES
ERR985389 [4]	Kapit	0.998	0.951	89.5	97.58	0.954	Mf-Pk	YES
ERR985390 [4]	Kapit	0.998	0.951	82.8	97.46	0.996	Mf-Pk	YES
ERR985391 [4]	Kapit	0.996	0.952	106.7	93.66	0.999	Mf-Pk	YES
ERR985392 [4]	Kapit	0.998	0.951	94.2	96.21	0.999	Mf-Pk	YES
ERR985393 [4]	Kapit	0.986	0.945	61.9	77.45	0.976	Mf-Pk	YES
ERR985394 [4]	Kapit	0.991	0.953	80	95.42	0.999	Mf-Pk	YES
ERR985395 [4]	Kapit	0.989	0.971	101.7	85.6	0.4	Mf-Pk	-
ERR985396 [4]	Kapit	0.97	0.956	64.2	59.26	0.767	Mf-Pk	-
ERR985397 [4]	Kapit	0.994	0.965	95.1	91.96	0.781	Mf-Pk	-
ERR985398 [4]	Kapit	0.968	0.95	45.3	59.68	0.999	Mf-Pk	YES
ERR985399 [4]	Kapit	0.994	0.958	105.9	97.35	0.999	Mf-Pk	YES
ERR985400 [4]	Kapit	0.986	0.954	59.5	84.14	0.997	Mf-Pk	YES
ERR985401 [4]	Kapit	0.994	0.954	67.4	95.63	0.999	Mf-Pk	YES
ERR985402 [4]	Kapit	0.988	0.958	148.8	92.36	0.999	Mf-Pk	YES
ERR985403 [4]	Kapit	0.992	0.961	149.9	95.37	0.999	Mf-Pk	YES
ERR985404 [4]	Kapit	0.993	0.964	152.7	94.49	0.964	Mf-Pk	YES
ERR985405 [4]	Kapit	0.997	0.961	93	94.81	0.569	Mf-Pk	-
ERR985406 [4]	Kapit	0.996	0.951	99.9	94.51	0.999	Mf-Pk	YES
ERR985407 [4]	Kapit	0.996	0.951	96.8	96.05	0.999	Mf-Pk	YES
ERR985408 [4]	Kapit	0.998	0.953	108.2	97.26	0.999	Mf-Pk	YES
ERR985409 [4]	Kapit	0.998	0.954	95.5	96.72	0.93	Mf-Pk	-
ERR985410 [4]	Betong	0.996	0.963	93.4	97.13	0.525	Mn-Pk	-
ERR985411 [4]	Betong	0.992	0.951	55.8	80.53	0.998	Mn-Pk	YES
ERR985412 [4]	Kapit	0.955	0.949	39.7	45.43	0.997	Mn-Pk	YES
ERR985413 [4]	Kapit	0.994	0.944	81.8	87.92	0.999	Mn-Pk	YES
ERR985414 [4]	Kapit	0.996	0.956	193	93.88	0.998	Mn-Pk	YES
ERR985415 [4]	Kapit	0.996	0.957	177	92.48	0.998	Mn-Pk	YES
ERR985416 [4]	Kapit	0.993	0.959	167.6	92.71	0.999	Mn-Pk	YES
ERR985417 [4]	Kapit	0.992	0.962	99.6	95.94	0.59	Mn-Pk	-
ERR985418 [4]	Kapit	0.994	0.954	147.4	89.52	0.999	Mn-Pk	YES

ERR985419 [4]	Kapit	0.988	0.956	142.2	80.41	0.903	Mn-Pk	-
ERR9751935	Pahang	0.79	0.056	14.6	13.51	1	Pen-Pk	-
ERR9751936	Johor	0.926	0.262	10.5	32.68	0.932	Pen-Pk	-
ERR9751937	Lab*	0.974	0.348	17.3	98.59	0.999	Pen-Pk	YES
ERR9751938	Lab	0.997	0.869	11.8	61.7	1	Pen-Pk	-
ERR9751932	Lab	0.999	0.907	15.4	80.36	1	Pen-Pk	-
ERR9751933	Lab	0.999	0.776	10.5	83.21	1	Pen-Pk	-
ERR9751934	Lab	1	0.845	11.7	99.62	1	Pen-Pk	-
ERR9751939	Malaysia	0.781	0.494	10.7	13.73	0.992	Pen-Pk	YES
ERR9751940	Indonesia	0.366	0.054	8.4	7.91	0.999	Mf-Pk	-
ERR2762859 [5]	Sabah	0.776	0.881	11.7	14.49	1	Mf-Pk	YES
ERR2762860 [5]	Sabah	0.71	0.774	9	11.13	0.726	Mf-Pk	-
ERR2762864 [5]	Sabah	0.99	0.956	68.7	85.02	0.961	Mf-Pk	YES
ERR2762867 [5]	Sabah	0.71	0.34	7.2	11.8	1	Mf-Pk	-
ERR2762870 [5]	Sabah	0.897	0.956	48.5	29.15	0.792	Mf-Pk	-
ERR2762872 [5]	Sabah	0.967	0.949	56.5	53.56	0.999	Mn-Pk	YES
ERR2762882 [5]	Sabah	0.676	0.682	36	20.67	0.996	Mn-Pk	YES
ERR2762883 [5]	Sabah	0.627	0.734	41.4	22.16	0.999	Mf-Pk	YES
ERR2762884 [5]	Sabah	0.532	0.631	29.8	17.26	0.999	Mf-Pk	YES
ERR2762885 [5]	Sabah	0.517	0.289	27.5	12.46	0.998	Mf-Pk	YES
ERR2762886 [5]	Sabah	0.722	0.876	86.1	93.86	0.818	Mf-Pk	-
ERR2762887 [5]	Sabah	0.655	0.57	31	38.29	0.998	Mf-Pk	YES
ERR2762888 [5]	Sabah	0.703	0.459	27.5	60.51	0.804	Mf-Pk	-
ERR2762889 [5]	Sabah	0.788	0.6	32.7	45.17	0.998	Mf-Pk	YES
ERR9751941	Perak	0.738	0.054	9.4	10.1	0.999	Pen-Pk	-
ERR9751942	Sabah	0.526	0.054	12.5	10.13	1	Mf-Pk	-
ERR9751943	Sabah	0.496	0.045	12.9	11.35	0.999	Mf-Pk	-
ERR9751944	Sabah	0.546	0.042	15.1	13.1	1	Mf-Pk	-
ERR9751945	Selangor	0.938	0.294	10.5	33.82	0.954	Pen-Pk	YES
SRR2221468 [4]	Lab	0.998	0.957	73.5	96.08	0.999	Pen-Pk	YES
SRR2222335 [4]	Lab	1	0.972	79	98.61	1	Pen-Pk	YES
SRR2225467 [4]	Lab	0.999	0.991	52	86.41	1	Pen-Pk	-
SRR2225571 [4]	Lab	0.997	0.954	56.9	74.49	0.999	Pen-Pk	YES
SRR2225573 [4]	Lab	0.998	0.959	83.7	97.06	0.999	Pen-Pk	YES
SRR3135172 [4]	Lab	0.999	0.989	64.8	93.01	1	Pen-Pk	-
ERR9751954	Lab*	0.987	0.618	14.8	96.63	0.999	Pen-Pk	YES
ERR9751946	Sarikei	0.948	0.912	21.3	50.6	0.647	Mn-Pk	-
ERR9751947	Sarikei	0.721	0.625	8.9	15.95	0.999	Mf-Pk	YES
ERR9751948	Sarikei	0.565	0.135	7.2	9.02	0.999	Mn-Pk	-
ERR9751949	Sarikei	0.859	0.798	12.6	28.61	0.999	Mf-Pk	YES
ERR9751950	Sarikei	0.932	0.893	18.1	48.86	0.997	Mf-Pk	YES
ERR9751951	Sarikei	0.808	0.683	9.4	20.81	0.705	Mn-Pk	-
ERR9751953	Sarikei	0.475	0.068	7.3	7.98	0.945	Mn-Pk	-
ERR9751952	Sarikei	0.971	0.857	14.3	71.25	0.999	Mn-Pk	YES
ERR2762890 [5]	Peninsula	0.925	0.252	60	99.9	1	Pen-Pk	-
ERR2762891 [5]	Peninsula	0.98	0.28	42.4	99.99	0.992	Pen-Pk	-
ERR2762892 [5]	Peninsula	0.993	0.527	39.4	99.98	0.998	Pen-Pk	YES

Coverage 5X is the proportion of the genome that is covered at least 5-fold; Population genetics included represents 104 samples used for the population genetics analysis; Lab* - samples derived from the *Macaca fascicularis* blood.

S2 Table. List of genome fragments representing the 1% of highest identity by descent (IBD) fraction values for each of the clusters

Chr	Start	End	Cluster	IBD fraction	Genes
**2**	**90001**	**100000**	Mf-Pk	0.0562	PKNH_0201600
3	660001	670000	Mf-Pk	0.0617	PKNH_0313600 (CRMP2)
4	450001	460000	Mf-Pk	0.0364	PKNH_0410200
**7**	**1310001**	**1320000**	Mf-Pk	0.0268	-
**7**	**1320001**	**1330000**	Mf-Pk	0.0337	PKNH_0729600 (NFU1), PKNH_0729700
8	30001	40000	Mf-Pk	0.0307	PKNH_0800600
8	910001	920000	Mf-Pk	0.0292	PKNH_0820000 (DOZI), PKNH_0820100 (SPC2), PKNH_0820200
8	920001	930000	Mf-Pk	0.0246	PKNH_0820300, PKNH_0820500
8	940001	950000	Mf-Pk	0.0835	PKNH_0820800 (Cap380)
8	1260001	1270000	Mf-Pk	0.0411	PKNH_0826900 (CTRP), PKNH_0827000 (eIF4E)
**8**	**1280001**	**1290000**	Mf-Pk	0.0682	-
**8**	**1760001**	**1770000**	Mf-Pk	0.027	PKNH_0838100 (EF-Ts), PKNH_0838200, PKNH_0838400
**8**	**1770001**	**1780000**	Mf-Pk	0.0499	PKNH_0838400, PKNH_0838500 (CSP), PKNH_0838600 (GLP1), PKNH_0838700 (RPL44), PKNH_0838800
8	1780001	1790000	Mf-Pk	0.0308	PKNH_0838800, PKNH_0838900 (EHD), PKNH_0839000 (IMC1e)
8	1790001	1800000	Mf-Pk	0.0366	PKNH_0839000 (IMC1e), PKNH_0839100 (IMC1a), PKNH_0839200, PKNH_0839300
**9**	**40001**	**50000**	Mf-Pk	0.0366	PKNH_0900200, PKNH_0900300
**9**	**860001**	**870000**	Mf-Pk	0.0247	PKNH_0919500
11	940001	950000	Mf-Pk	0.0252	PKNH_1120100, PKNH_1120200 (PL)
11	1420001	1430000	Mf-Pk	0.0398	PKNH_1130100, PKNH_1130200, PKNH_1130300
11	1430001	1440000	Mf-Pk	0.0249	PKNH_1130400 (SF3A2), PKNH_1130500
11	1440001	1450000	Mf-Pk	0.0522	PKNH_1130600, PKNH_1130700, PKNH_1130800
11	1450001	1460000	Mf-Pk	0.0676	PKNH_1130800, PKNH_1130900, PKNH_1131000, PKNH_1131100
**11**	**1810001**	**1820000**	Mf-Pk	0.0591	PKNH_1138900
12	2420001	2430000	Mf-Pk	0.0347	PKNH_1253900 (WDR92), PKNH_1254000, PKNH_1254100 (P47)
3	990001	1000000	Mn-Pk	0.1731	PKNH_0321600
4	450001	460000	Mn-Pk	0.2347	PKNH_0410200
5	250001	260000	Mn-Pk	0.1674	PKNH_0505800, PKNH_0505900
6	320001	330000	Mn-Pk	0.1577	PKNH_0606900, PKNH_0607000, PKNH_0607100 (GcpE), PKNH_0607200
6	1050001	1060000	Mn-Pk	0.1644	PKNH_0623600, PKNH_0623700, PKNH_0623800
7	10001	20000	Mn-Pk	0.1998	PKNH_0700200 (NBPXb)
7	610001	620000	Mn-Pk	0.1495	PKNH_0713000 (NDH2), PKNH_0713100 (UBC9), PKNH_0713200, PKNH_0713300
7	1040001	1050000	Mn-Pk	0.1557	-
8	980001	990000	Mn-Pk	0.1478	PKNH_0821700, PKNH_0821800, PKNH_0821900
8	1070001	1080000	Mn-Pk	0.1745	PKNH_0823100, PKNH_0823200
**8**	**1760001**	**1770000**	Mn-Pk	0.1592	PKNH_0838100 (EF-Ts), PKNH_0838200, PKNH_0838400
**8**	**1770001**	**1780000**	Mn-Pk	0.1449	PKNH_0838400, PKNH_0838500 (CSP), PKNH_0838600 (GLP1), PKNH_0838700 (RPL44), PKNH_0838800
**9**	**40001**	**50000**	Mn-Pk	0.2016	PKNH_0900200, PKNH_0900300
**9**	**860001**	**870000**	Mn-Pk	0.1517	PKNH_0919500
9	1330001	1340000	Mn-Pk	0.1628	PKNH_0930000, PKNH_0930100
9	1810001	1820000	Mn-Pk	0.1575	PKNH_0939600, PKNH_0939700 (IMC1b), PKNH_0939800
9	2070001	2080000	Mn-Pk	0.1539	PKNH_0945200, PKNH_0945400
**10**	**1040001**	**1050000**	Mn-Pk	0.2457	PKNH_1022900
11	1740001	1750000	Mn-Pk	0.1629	PKNH_1138300
12	40001	50000	Mn-Pk	0.1832	-
12	50001	60000	Mn-Pk	0.225	-
12	60001	70000	Mn-Pk	0.2281	-
12	2890001	2900000	Mn-Pk	0.1411	PKNH_1264700, PKNH_1264800, PKNH_1265000, PKNH_1265100, PKNH_1265200
14	840001	850000	Mn-Pk	0.2157	PKNH_1418800, PKNH_1418900 (G3PAT), PKNH_1419000
1	240001	250000	Pen-Pk	0.1108	PKNH_0103900 (MCM7), PKNH_0104000
**2**	**90001**	**100000**	Pen-Pk	0.1828	PKNH_0201600
6	300001	310000	Pen-Pk	0.0913	PKNH_0606600 (ZIPCO), PKNH_0606700 (SRSF4)
6	1010001	1020000	Pen-Pk	0.084	PKNH_0622900, PKNH_0623000
7	1240001	1250000	Pen-Pk	0.1078	PKNH_0727800, PKNH_0727900 (RhopH2), PKNH_0728000
**7**	**1310001**	**1320000**	Pen-Pk	0.1056	-
**7**	**1320001**	**1330000**	Pen-Pk	0.1104	PKNH_0729600 (NFU1), PKNH_0729700
**8**	**1280001**	**1290000**	Pen-Pk	0.088	-

**9**	**40001**	**50000**	Pen-Pk	0.0849	PKNH_0900200, PKNH_0900300
9	2130001	2140000	Pen-Pk	0.0944	PKNH_0946200
**10**	**1040001**	**1050000**	Pen-Pk	0.1137	PKNH_1022900
10	1050001	1060000	Pen-Pk	0.0834	PKNH_1023100
11	1810001	1820000	Pen-Pk	0.1119	PKNH_1138900
12	2090001	2100000	Pen-Pk	0.1284	PKNH_1246400 (ETRAMP), PKNH_1246500, PKNH_1246600
12	2100001	2110000	Pen-Pk	0.1151	PKNH_1246800
12	2160001	2170000	Pen-Pk	0.1101	PKNH_1247800, PKNH_1247900
12	2820001	2830000	Pen-Pk	0.0845	PKNH_1262900, PKNH_1263000, PKNH_1263100
13	2160001	2170000	Pen-Pk	0.1248	PKNH_1347800, PKNH_1347900 (RAP1)
13	2180001	2190000	Pen-Pk	0.0932	PKNH_1348000, PKNH_1348100, PKNH_1348200
13	2200001	2210000	Pen-Pk	0.103	PKNH_1348500 (CELF2), PKNH_1348600
13	2210001	2220000	Pen-Pk	0.1125	PKNH_1348600, PKNH_1348700, PKNH_1348800
14	1800001	1810000	Pen-Pk	0.0928	PKNH_1441600 (DPH6)
14	2200001	2210000	Pen-Pk	0.1077	PKNH_1450600
14	3190001	3200000	Pen-Pk	0.1377	PKNH_1472200, PKNH_1472300 (NBPXa)

Bold fragments appeared in multiple clusters.

S3 Table. Genes with fixed SNPS (Fst=1) between sub-populations

Gene [PKNH_]	Name	Chr	Start	End	Length [bp]	Mf-Pk vs Mn-Pk No. fixed SNPs [No. non-syn]	Pen-Pk vs Mf-Pk No. fixed SNPs [No. non-syn]	Pen-Pk vs Mn-Pk No. fixed SNPs [No. non-syn]
0101300		1	63995	64531	536	-	-	1
0101400		1	65573	69472	3899	-	-	1 [1]
0101700		1	79664	86719	7055	-	1	1
0101800		1	88814	90973	2159	-	2 [1]	2 [1]
0102100		1	99863	111634	11771	-	1	1
0102200		1	113100	121946	8846	-	2 [1]	5 [4]
0102300		1	125067	135359	10292	-	4 [2]	4 [2]
0102600		1	150569	152986	2417	-	-	1
0102700		1	154818	156504	1686	-	2 [1]	2 [1]
0103100		1	191598	204382	12784	-	1	1
0104000		1	243142	251284	8142	-	1	3 [1]
0104100		1	254213	257434	3221	-	1	1
0104200		1	258769	259572	803	-	1	2
0104300		1	260877	262795	1918	-	2	5
0104400		1	263793	265031	1238	-	2	2
0104600		1	270859	276533	5674	-	3 [1]	3 [1]
0105000		1	283906	289215	5309	-	15 [4]	16 [4]
0105200		1	299231	301798	2567	-	1 [1]	1 [1]
0105700		1	306268	311910	5642	-	1 [1]	1 [1]
0105800	RAMA	1	315258	318252	2994	-	6 [4]	8 [6]
0105900		1	320280	322295	2015	-	3 [1]	4 [1]
0106000		1	324428	328540	4112	-	-	1 [1]
0106200		1	334654	337638	2984	-	5 [1]	5 [1]
0106500		1	345784	348515	2731	-	2 [1]	1
0107200	IMC1d	1	368061	368993	932	-	1	2
0107400	HSP110c	1	374489	377167	2678	-	1 [1]	1 [1]
0107600	CRT	1	381348	384824	3476	-	4	8
0107700		1	387249	390413	3164	-	2 [1]	3 [2]
0107900		1	392508	399983	7475	-	3	4 [1]
0108300		1	412774	414315	1541	-	-	2 [1]
0108400		1	415931	417184	1253	-	-	2 [1]
0108600		1	420076	427302	7226	-	2 [1]	1 [1]
0108800		1	441319	442836	1517	-	4 [3]	6 [3]
0109100	RAD14	1	453385	454656	1271	-	1	1
0109500		1	462536	463642	1106	-	-	1 [1]
0109700		1	466054	469452	3398	-	3 [1]	5 [3]
0109900		1	475045	477648	2603	-	-	2
0110100	SAP18	1	479882	482128	2246	-	-	1 [1]
0110200		1	485535	487601	2066	-	4 [3]	5 [3]
0110300	SEC28	1	490021	491049	1028	-	1	2
0110400		1	492161	493146	985	-	-	2 [1]
0110600		1	498960	503491	4531	-	7 [5]	7 [6]
0110800	TyrRS	1	508886	510506	1620	-	1	1
0110900	RPN10	1	511925	513731	1806	-	-	1
0111000	DegP	1	514272	515501	1229	-	1	2
0111500	RAB18	1	529714	530910	1196	-	1	1
0111600		1	533482	534879	1397	-	3 [1]	6 [1]
0112000		1	546375	549496	3121	-	-	1
0112100	PTP2	1	550141	551547	1406	-	1	-
0112300		1	560095	563321	3226	-	4 [2]	3 [2]
0112400		1	565203	567542	2339	-	1	1
0112600		1	570099	575455	5356	-	7 [2]	5 [1]
0112700		1	575517	579893	4376	-	3 [2]	3 [2]
0112800		1	580861	582438	1577	-	2 [2]	2 [2]
0113000		1	587206	588802	1596	-	1	1
0114800	METAP1c	1	695298	697319	2021	-	1 [1]	1 [1]
0114900		1	698276	700243	1967	-	1	1
0115200		1	709117	710475	1358	1	1	-
0115300		1	712292	715193	2901	-	6 [2]	9 [4]
0115500	RAD54	1	723002	726160	3158	-	1 [1]	1 [1]
0115600		1	727456	729391	1935	-	-	1 [1]
0115700		1	731756	733654	1898	-	2	1
0116000		1	740668	741264	596	-	2 [1]	-

0116100	CNA	1	741813	744277	2464	-	1	1
0116200		1	746154	747675	1521	1	-	-
0116600	PWP2	1	765066	768215	3149	-	5 [2]	-
0116900	GDH3	1	781877	785479	3602	-	2	-
0117200	SENP2	1	809152	814338	5186	-	2 [1]	1 [1]
0117400	NOL10	1	818601	820343	1742	-	1	-
0117500		1	822843	825882	3039	-	1 [1]	-
0117600	WARP	1	827233	828117	884	-	-	1 [1]
0117900		1	842628	845732	3104	-	4 [1]	-
0118200	REX3	1	855842	856728	886	-	1 [1]	-
0200400		2	38672	41062	2390	1 [1]	-	-
0200500		2	44808	45962	1154	4 [1]	1 [1]	-
0200600		2	50114	51616	1502	7 [4]	7 [4]	-
0200800		2	59014	60094	1080	8 [4]	2 [1]	1
0200900		2	64876	66926	2050	3 [2]	-	1 [1]
0201100		2	71998	73130	1132	3 [1]	-	-
0201200		2	77527	78590	1063	9 [7]	2 [2]	-
0201300		2	84774	87586	2812	3 [1]	2 [1]	3
0202100		2	112378	114260	1882	-	1 [1]	2 [1]
0202500		2	125988	130737	4749	-	1	-
0203300	PIP5K	2	168701	174095	5394	-	1 [1]	3
0203400		2	176404	181508	5104	9 [4]	2 [1]	5 [3]
0203500	RPB9	2	182533	183416	883	-	-	1
0203950		2	189996	193575	3579	-	-	6
0204200		2	198176	199126	950	-	-	1
0204300		2	199966	200626	660	-	1 [1]	1 [1]
0204500		2	203872	204987	1115	-	1 [1]	1 [1]
0204700	CCp5	2	206770	210102	3332	-	2 [1]	6 [4]
0204900		2	214738	217428	2690	-	2	2
0205200		2	225501	226160	659	1	-	-
0205300		2	228602	231297	2695	-	1	2
0205800	MRE11	2	249118	252405	3287	-	1	1
0206100		2	272534	277251	4717	6 [4]	2 [2]	2 [1]
0206500	CEN1	2	284338	285748	1410	-	-	2
0206600	IspD	2	286622	288535	1913	-	-	1 [1]
0206800		2	293178	296786	3608	1	1	-
0207200	ATP6	2	308447	312237	3790	-	2	2
0208100		2	381789	385960	4171	-	-	1 [1]
0208900		2	403793	407452	3659	-	-	3 [2]
0209000		2	407978	408847	869	-	2	-
0209100	LytB	2	409390	410796	1406	-	-	1 [1]
0209200	UBP1	2	411614	422485	10871	7 [3]	10 [4]	2
0209300		2	425737	427167	1430	5 [4]	1 [1]	1 [1]
0209400		2	428512	431051	2539	4 [2]	4 [3]	3 [2]
0209600		2	435514	437004	1490	1	-	-
0209900		2	445213	449758	4545	-	3 [1]	1
0210000		2	450596	456664	6068	-	4 [3]	7 [5]
0210100		2	458624	463144	4520	-	2 [1]	1 [1]
0210700		2	484687	486573	1886	-	1	-
0211000		2	494939	508693	13754	-	1 [1]	2 [1]
0211600	VPS53	2	533652	536201	2549	-	-	1 [1]
0211800	NFS	2	541953	543503	1550	-	1	-
0214800		2	680605	695784	15179	-	1 [1]	1 [1]
0215100		2	700924	704312	3388	-	-	1
0215200	AP2-L	2	712473	715649	3176	-	3 [2]	7 [4]
0215300		2	718708	719584	876	-	1	1
0215400		2	720939	730217	9278	-	4 [2]	17 [11]
0215700		2	736955	737539	584	-	-	1 [1]
0300900		3	48954	50717	1763	-	-	2
0301200		3	56536	68064	11528	-	1	1 [1]
0301500		3	73651	76299	2648	-	-	2
0301600		3	78120	80848	2728	-	6	6
0301700	CLF1	3	88317	91223	2906	-	3 [1]	1
0301900		3	102672	106974	4302	3	4 [3]	7 [4]
0302100	ApiAP2	3	109279	119928	10649	-	4 [2]	10 [7]
0302300		3	125044	128458	3414	-	1	2
0302400	P36	3	130534	131595	1061	-	-	2
0302500	P52	3	133324	134787	1463	-	1 [1]	2 [1]

0302700		3	145301	157522	12221	-	11 [3]	31 [13]
0302800	DPAP3	3	157898	160344	2446	-	-	3 [1]
0302900		3	161342	163479	2137	-	-	3 [2]
0303800		3	209804	216463	6659	-	5 [2]	14 [5]
0304000	RON1	3	221540	224225	2685	1	-	4 [1]
0304100		3	227295	228767	1472	1	-	1
0304300		3	235157	235579	422	-	-	1
0304900		3	254725	263202	8477	-	2	3
0305100		3	266174	267314	1140	-	1 [1]	1 [1]
0305600		3	279101	282538	3437	-	2 [2]	4 [3]
0305900		3	295954	298209	2255	-	1	1
0306200		3	306874	308580	1706	-	1	-
0306300		3	309079	310895	1816	-	2	2
0306600	SIAP1	3	321088	324084	2996	5 [2]	-	-
0306700	PLP1	3	325085	328942	3857	-	-	1 [1]
0307000		3	337169	343492	6323	-	-	1 [1]
0307700	RPA1	3	372151	375726	3575	-	1 [1]	1
0308100	EVP1	3	386308	388084	1776	-	3 [3]	2 [2]
0308900		3	407817	411713	3896	-	5 [4]	7 [5]
0309000		3	412266	414599	2333	-	1	1
0309300		3	422035	424938	2903	-	1 [1]	1 [1]
0309900		3	435037	439707	4670	-	2 [1]	2 [1]
0310200		3	463395	465365	1970	-	1	-
0310300		3	466747	467706	959	-	2	1
0310400		3	468302	469381	1079	-	1	1
0310600		3	473003	476047	3044	1	-	1
0310700	SDA1	3	476939	479818	2879	1	2 [2]	3 [2]
0310900		3	484530	486272	1742	-	1 [1]	-
0311000		3	487378	488479	1101	6	1	2
0311100		3	488713	491411	2698	1	-	-
0311200	SufS	3	491915	493592	1677	1 [1]	-	1 [1]
0311300		3	494190	497072	2882	1	-	-
0312100		3	550364	552870	2506	3	-	-
0312500	CDPK4	3	563801	565538	1737	-	-	1
0312800		3	575414	579448	4034	-	1 [1]	1 [1]
0313000		3	584544	601977	17433	-	1	1
0313100	EST	3	603369	611582	8213	-	1 [1]	1 [1]
0314800		3	741467	745294	3827	-	1 [1]	1 [1]
0314900		3	746992	748574	1582	-	1	1
0315200		3	754731	756206	1475	-	1	1
0315400	CSL4	3	765505	766354	849	-	2 [1]	2 [1]
0315500		3	766490	767461	971	-	-	1 [1]
0315800		3	771301	773280	1979	-	4 [2]	2 [2]
0316100		3	779170	785749	6579	-	10 [3]	10 [4]
0316600		3	802942	805632	2690	-	7 [5]	7 [5]
0316700	DBP7	3	806554	808893	2339	-	4 [1]	3 [1]
0317000		3	814757	815492	735	-	1	1
0317100		3	818974	820392	1418	-	2 [1]	2 [1]
0317700		3	831665	834544	2879	-	2	2 [1]
0318200		3	844101	845927	1826	-	2 [1]	2 [1]
0318700		3	857946	859283	1337	1 [1]	-	2 [1]
0318800		3	859716	864059	4343	-	1	1
0318900		3	865347	866042	695	-	-	1
0319200		3	872378	878471	6093	-	2 [1]	1 [1]
0319300		3	878936	883609	4673	-	3	3
0319400		3	885944	890866	4922	-	6 [4]	10 [7]
0319500		3	892388	895510	3122	-	3 [3]	4 [4]
0319700	COQ3	3	901597	902885	1288	-	2 [1]	2 [1]
0319900		3	905504	906871	1367	-	1	-
0320000		3	907274	913490	6216	-	2 [2]	3 [2]
0320300		3	932757	939386	6629	-	1 [1]	2 [2]
0320400		3	940480	944097	3617	-	-	1 [1]
0320600		3	950677	951696	1019	-	-	2
0321400	ETRAMP	3	982930	983607	677	3 [1]	-	-
0400300		4	29636	31419	1783	1 [1]	1 [1]	-
0400500		4	42215	43190	975	-	1 [1]	-
0400700		4	49884	51871	1987	7 [5]	2 [2]	1 [1]
0400800		4	54677	55937	1260	6 [2]	1 [1]	-

0400900		4	58006	64156	6150	26 [16]	7 [6]	7 [3]
0401300	RFC1	4	78560	81343	2783	-	5 [4]	6 [3]
0402300		4	98594	104104	5510	-	3 [2]	2 [1]
0402600		4	110136	111726	1590	-	1	2
0402900	RFC2	4	120099	121091	992	-	1	1
0403000		4	123156	124745	1589	-	-	3 [2]
0403200		4	127966	129180	1214	-	1	2 [1]
0403700		4	145551	149573	4022	-	2	3
0403800		4	149827	151485	1658	-	1	1
0403900		4	153447	156810	3363	-	-	2 [1]
0404000		4	157083	158582	1499	-	-	2 [2]
0404200	ATG11	4	163344	168699	5355	-	1	2 [1]
0404300		4	170504	172357	1853	-	1 [1]	1 [1]
0404800		4	189298	192603	3305	-	7 [5]	9 [7]
0404900		4	192902	197638	4736	-	5 [4]	6 [5]
0405000	DHHC11	4	199199	201486	2287	-	-	1
0405100	ORC5	4	203040	205538	2498	1	2	-
0405200	RPB2	4	205963	210144	4181	-	4	6
0405400		4	213767	215821	2054	-	1 [1]	2 [1]
0405600		4	221485	222186	701	1	1	3
0405700		4	224478	226417	1939	1	2	8
0405800		4	228688	230889	2201	11 [6]	4 [4]	1 [1]
0405900	RON6	4	232104	237073	4969	23 [4]	1 [1]	6 [2]
0406000		4	238925	243039	4114	3 [3]	2	5 [1]
0406100		4	246024	248166	2142	-	-	1
0406200		4	249423	254498	5075	-	3 [2]	3 [2]
0406900		4	279124	282048	2924	-	-	1 [1]
0408700		4	378318	389573	11255	-	9 [6]	7 [5]
0408800		4	392035	402532	10497	-	3 [1]	6 [2]
0409000		4	407549	408546	997	-	2 [2]	3 [3]
0409100		4	409369	414012	4643	-	8 [6]	9 [6]
0409400		4	421214	422974	1760	1	3	-
0409500	TKL1	4	424782	427892	3110	-	3 [2]	3 [2]
0409700		4	430202	432443	2241	-	-	4
0409900		4	439325	442185	2860	-	2 [1]	6 [1]
0410400		4	466106	467320	1214	-	-	3
0410500		4	468398	469345	947	-	1	2
0410600		4	471712	473822	2110	-	2 [1]	3 [1]
0410700		4	474356	478057	3701	-	1	5 [2]
0410900		4	480655	482189	1534	-	3	3
0411300	UAP56	4	498476	500192	1716	-	4 [2]	5 [2]
0411500		4	504892	509047	4155	3 [2]	6 [3]	5 [1]
0411900		4	517174	519117	1943	-	-	2
0412000		4	520930	523532	2602	-	2 [1]	8 [5]
0412100	P230	4	524538	532889	8351	-	3 [1]	6 [3]
0412200	P230p	4	534495	541265	6770	-	3 [2]	4 [2]
0412600		4	549225	550068	843	1 [1]	1 [1]	-
0412700		4	553740	559835	6095	3 [2]	2 [2]	5 [5]
0412800		4	560423	564757	4334	-	-	1 [1]
0413000		4	567018	573912	6894	-	3	5 [1]
0413100		4	575774	579361	3587	-	1	2 [1]
0413500		4	595724	597895	2171	-	-	1
0413600		4	599262	602910	3648	2	3 [1]	3 [1]
0413800		4	609853	612753	2900	-	3	13 [1]
0414000		4	615591	620960	5369	-	-	4 [3]
0414100		4	624499	625288	789	-	-	2 [2]
0414200		4	626516	627888	1372	-	-	2 [2]
0414300	ASL	4	628569	629963	1394	-	-	1
0414400		4	630842	631328	486	-	-	3 [1]
0415000	RPN1	4	659371	662801	3430	-	-	2
0415200		4	665738	667251	1513	-	1	4
0415300		4	668628	670640	2012	1	1	-
0415500		4	673699	675069	1370	2	2	1
0415600		4	676142	678779	2637	-	3	3 [2]
0415700		4	679656	680794	1138	-	-	3 [1]
0415800		4	681415	683535	2120	5 [3]	-	4 [2]
0415900		4	684089	685987	1898	10 [3]	-	-
0416100		4	688351	689505	1154	1	-	2

0416300		4	695845	698577	2732	-	2 [1]	-
0416500		4	702134	702989	855	2	-	3
0417100		4	743842	746325	2483	-	-	1
0417300		4	750383	754104	3721	1	-	-
0417500		4	757987	759600	1613	3 [1]	1 [1]	2
0417900		4	766115	767464	1349	-	-	1 [1]
0418100	ROPE	4	778295	783859	5564	3 [3]	-	11 [8]
0418200	DHHC12	4	784195	786309	2114	-	1	3
0418300		4	786849	787903	1054	-	-	2
0418400	OPP	4	788544	790112	1568	-	1 [1]	3 [2]
0418500		4	791134	794901	3767	2 [2]	1 [1]	5 [3]
0418700		4	802945	804147	1202	1 [1]	1	2 [1]
0418800		4	806947	812082	5135	1	1	-
0419000	LRR5	4	834835	841642	6807	2 [1]	-	2
0419500		4	855568	856489	921	-	-	1
0419800		4	862942	866292	3350	-	-	1
0420100	TOP2	4	892480	897651	5171	1	7 [1]	10 [3]
0420300		4	902745	908493	5748	-	-	3 [3]
0420400		4	909659	916507	6848	2	10 [3]	10 [6]
0420500		4	919615	925362	5747	-	-	5 [2]
0420600	CAF16	4	925717	927342	1625	-	-	2
0421000		4	937055	938242	1187	-	-	1
0421100		4	939944	961738	21794	-	5 [3]	8 [5]
0421200		4	963365	964996	1631	-	-	1
0421500		4	971056	972703	1647	-	-	3 [1]
0421900		4	981567	990626	9059	-	1 [1]	9 [2]
0422500		4	1015236	1016168	932	-	1	2
0422600		4	1018579	1020357	1778	-	1	11 [4]
0422700		4	1022201	1027227	5026	-	3 [1]	6 [2]
0422800		4	1027233	1035065	7832	-	-	5 [2]
0423000		4	1042366	1045362	2996	-	2 [1]	4 [2]
0424200		4	1098930	1099727	797	-	-	1 [1]
0501000		5	58981	60375	1394	-	4 [2]	5 [3]
0501100		5	63169	64401	1232	-	3 [2]	2 [1]
0501200		5	67260	71448	4188	-	2	2
0501300	PBLP	5	72938	75888	2950	-	7	7
0501400		5	78706	79562	856	-	3	5
0502300	RON5	5	115925	123893	7968	-	-	1 [1]
0503100		5	163695	165746	2051	-	1 [1]	1 [1]
0503800	ClpB1	5	182462	185614	3152	-	2	3
0504200	VPS2	5	197060	197707	647	-	1 [1]	1 [1]
0504300		5	197901	198774	873	-	1	1
0504600	VPS9	5	205832	209809	3977	-	5 [3]	2 [2]
0504700		5	211280	212579	1299	-	1 [1]	1 [1]
0504800	EIF3G	5	214003	216294	2291	-	1	1
0504900		5	216407	218571	2164	-	2	2
0505100	PGM2	5	221714	222610	896	-	1 [1]	1 [1]
0505200	RPT3	5	223100	224436	1336	-	-	1
0505400		5	229220	230074	854	-	1	1
0505600	SMC3	5	234416	241465	7049	-	-	1
0505700		5	242514	247428	4914	-	2 [2]	3 [1]
0505800		5	255397	256351	954	-	2 [1]	3 [1]
0506000		5	260708	262567	1859	-	1 [1]	2 [2]
0506100		5	263038	265389	2351	-	1	1
0506200		5	266664	267579	915	-	1	1
0506800		5	281433	286778	5345	-	1 [1]	1 [1]
0506900		5	289867	294115	4248	-	4 [3]	5 [3]
0507400		5	302396	304564	2168	-	2 [1]	2 [1]
0508100		5	391560	392761	1201	1	3 [2]	5 [2]
0508200		5	394353	395256	903	1 [1]	1 [1]	-
0508300		5	398561	399537	976	-	1 [1]	1 [1]
0508500	GATA	5	404906	406861	1955	-	1 [1]	2 [1]
0508800	HAT1	5	415337	419408	4071	-	-	1 [1]
0508900		5	419945	420730	785	-	1	1
0509500	PUF2	5	440077	442018	1941	-	3 [2]	5 [2]
0509600	DHFR-TS	5	445338	447218	1880	-	1	1
0509900		5	470374	472760	2386	-	1	2
0510100		5	478038	480287	2249	-	4 [3]	4 [3]

0510300		5	484905	487070	2165	-	1 [1]	1 [1]
0510400		5	487802	502437	14635	-	3 [2]	2 [2]
0510500		5	503358	507029	3671	-	-	1 [1]
0512700		5	595259	605335	10076	-	8 [5]	10 [8]
0512800	RIO2	5	609357	611273	1916	-	-	1 [1]
0513000	ACPS	5	618282	620168	1886	-	2	3 [1]
0513100	ApiAP2	5	627202	636774	9572	-	3 [3]	6 [5]
0513200	RRF2	5	638689	639537	848	-	1	-
0513300		5	639772	641160	1388	-	3 [1]	5 [1]
0513500		5	643577	650420	6843	-	3 [2]	3 [2]
0514400	BRR2	5	698801	706618	7817	-	11 [4]	8 [3]
0514600	EIF4A3	5	709007	710179	1172	-	2 [2]	2 [2]
0514700	SR12	5	711207	713622	2415	-	2 [1]	3 [1]
0514800		5	714092	715244	1152	-	1	2
0515200		5	720968	722542	1574	-	4 [2]	5 [3]
0515300	ARP	5	725234	725953	719	-	2 [2]	1 [1]
0515600		5	737467	738616	1149	-	-	1
0515900		5	753909	755197	1288	-	2 [1]	2 [1]
0516000	TryThrA	5	756886	758586	1700	-	2 [2]	3 [3]
0516100		5	762901	764162	1261	-	2 [1]	2 [2]
0516200		5	766855	768022	1167	-	1 [1]	1 [1]
0600300		6	21252	23570	2318	2 [1]	-	-
0600400		6	25089	26285	1196	3 [3]	-	-
0600600		6	33828	38051	4223	17 [9]	5 [2]	1 [1]
0600700		6	40055	41318	1263	6 [3]	1 [1]	-
0600800		6	43775	44858	1083	6 [5]	3 [3]	1 [1]
0600900		6	47680	48880	1200	5 [2]	-	1 [1]
0601000		6	51505	52595	1090	5 [2]	-	1
0601200		6	59566	62856	3290	3 [1]	1	4
0601400		6	66173	67992	1819	-	-	6 [1]
0602100	RPN5	6	88402	89948	1546	-	2 [2]	2 [2]
0602200		6	90835	92271	1436	-	1	1
0602400	PPP8	6	94120	99918	5798	-	8 [4]	8 [3]
0602500		6	101265	103697	2432	-	-	1
0602700		6	105704	106869	1165	-	2 [1]	2 [1]
0603200		6	122508	124703	2195	-	2 [2]	2 [1]
0603300		6	127959	134769	6810	-	9 [3]	8 [2]
0603400		6	135494	139822	4328	-	-	3 [3]
0603500		6	142322	147000	4678	-	4 [1]	8 [4]
0603700		6	156002	163433	7431	-	3 [1]	6 [1]
0603800		6	164869	167835	2966	-	8 [5]	9 [6]
0604000		6	171666	174386	2720	-	5 [2]	5 [3]
0604400		6	183865	186918	3053	-	5 [2]	6 [3]
0604500		6	189067	191136	2069	-	-	1
0604600		6	191392	193046	1654	-	2	4 [1]
0604700		6	193525	194910	1385	-	1	-
0604800		6	195362	200384	5022	-	1	1
0604900		6	201525	205763	4238	-	1	1
0605400		6	221018	225658	4640	-	6 [3]	6 [3]
0605500		6	227760	229553	1793	-	2 [1]	2 [1]
0605700		6	234218	236976	2758	-	1	1
0606100	SEA1	6	263461	269991	6530	-	2	3 [1]
0606300		6	285066	289612	4546	-	-	1 [1]
0606600	ZIPCO	6	298337	300010	1673	-	-	1
0606700	SRSF4	6	307984	310614	2630	5 [3]	-	8 [3]
0607100	GcpE	6	325644	328085	2441	-	-	1
0607500		6	355820	358339	2519	5 [4]	-	4 [2]
0607600		6	359227	374640	15413	-	1	3 [1]
0608100		6	381890	384827	2937	-	4	3
0608200		6	385919	388006	2087	1	3 [1]	2 [1]
0608600		6	413154	417215	4061	-	2 [1]	2 [1]
0608800		6	419666	420097	431	-	1	1
0608900		6	420687	422193	1506	-	1	3 [1]
0609000		6	423089	423811	722	-	5 [2]	5 [2]
0609100		6	423895	426145	2250	-	1	4 [1]
0609300		6	431320	435015	3695	1	2	3
0609400		6	436203	439268	3065	-	2 [1]	3 [1]
0609600		6	449119	451362	2243	-	-	1

0609700		6	451617	452645	1028	-	1 [1]	1 [1]
0609800		6	453465	456212	2747	-	4 [2]	5 [3]
0610500		6	492734	494243	1509	-	-	1
0610700		6	497599	499167	1568	-	2	3
0611100		6	506532	511706	5174	-	1	-
0611400		6	520530	525392	4862	-	9 [6]	11 [8]
0611500	MPP10	6	527608	529851	2243	-	2 [1]	-
0611600		6	531211	532884	1673	-	1	1
0612100	CEN3	6	543942	545343	1401	-	1	1
0612800		6	566171	567181	1010	-	2 [1]	2 [1]
0612900		6	567524	570547	3023	-	-	1
0613900		6	634402	636018	1616	-	2	-
0614600		6	658613	662896	4283	-	1	1
0614800	PRP22	6	665520	669963	4443	-	2	4
0615000		6	673967	677269	3302	-	1 [1]	1 [1]
0615400		6	690299	691093	794	-	1	-
0615600	P28	6	693212	693892	680	-	1 [1]	-
0616200		6	713375	717082	3707	-	5 [4]	5 [4]
0617200	PIGA	6	765879	768246	2367	-	1	1
0617700		6	785958	790418	4460	-	1	-
0617900		6	793831	798012	4181	10 [7]	-	2 [2]
0619100		6	867168	868385	1217	1	-	2
0620900		6	927209	929752	2543	2 [2]	-	-
0621000		6	931674	933002	1328	-	-	1 [1]
0621300		6	939437	943104	3667	-	1 [1]	-
0621400		6	944320	950253	5933	1 [1]	1	2 [2]
0621500		6	955186	961499	6313	1	-	3
0621600		6	962039	964189	2150	2 [1]	2 [1]	-
0621800		6	971051	971956	905	2	-	-
0621900		6	974728	977823	3095	-	-	1 [1]
0622000	DYN2	6	980569	982806	2237	-	1 [1]	2 [1]
0622100		6	984329	987634	3305	1	1	1
0622200		6	989446	990296	850	1 [1]	1 [1]	-
0622400		6	997409	999079	1670	6	-	1
0622500		6	1000371	1001648	1277	4	-	2
0622600		6	1002357	1005254	2897	11 [5]	-	2 [1]
0622700		6	1005931	1006313	382	3	-	-
0622800		6	1006601	1009060	2459	11 [2]	-	-
0622900		6	1011695	1012702	1007	5 [1]	-	-
0623000		6	1016552	1018888	2336	11 [7]	4 [2]	1 [1]
0623100		6	1022581	1023653	1072	7 [3]	-	1 [1]
0623200		6	1028878	1034433	5555	50 [34]	2 [2]	3 [2]
0623400		6	1038242	1039384	1142	5 [2]	-	1 [1]
0623500		6	1043496	1047311	3815	10 [5]	15 [11]	15 [12]
0623600		6	1049503	1050698	1195	13 [6]	-	-
0700400		7	24759	25775	1016	2 [1]	-	1 [1]
0700500		7	29304	30337	1033	6 [4]	1 [1]	6 [4]
0700700		7	37476	37985	509	1 [1]	-	-
0700800	RER1	7	38801	39580	779	2 [1]	-	-
0700900	RAB7	7	42155	44435	2280	3	-	1
0701100		7	61309	68789	7480	-	4 [2]	4 [2]
0701200		7	73029	76931	3902	-	-	1 [1]
0701500	CCp4	7	89268	93703	4435	-	1	-
0701600	RPL32	7	95751	96663	912	-	-	1
0701700		7	97755	98999	1244	-	-	1 [1]
0701800		7	100744	105833	5089	4	1	4 [2]
0701900		7	108235	109311	1076	-	-	1 [1]
0702000		7	109800	118511	8711	10 [4]	-	8 [3]
0702100	SPC3	7	119696	120253	557	-	-	1
0702300		7	125737	130561	4824	11 [7]	2 [1]	4 [3]
0702400		7	131554	132411	857	1	-	1
0702500		7	135685	137805	2120	-	2 [2]	6 [2]
0702600	CuTP	7	138497	144984	6487	15 [12]	-	2 [2]
0702700		7	146805	147629	824	-	1	1
0702800	NUP100	7	149034	155652	6618	4	3	9 [4]
0702900		7	158538	161885	3347	-	-	1 [1]
0703000		7	164877	182210	17333	8 [3]	3 [1]	16 [5]
0703100	RhopH3	7	183969	188105	4136	10 [2]	5 [3]	10 [2]

0703200		7	191612	192325	713	-	1 [1]	-
0703300	WDR66	7	194133	197642	3509	4 [1]	1 [1]	1 [1]
0703400	ATG3	7	197782	200378	2596	-	-	2
0703500		7	203910	208224	4314	-	-	2 [2]
0703600	SEC27	7	209296	212322	3026	-	-	2
0703700		7	216305	223525	7220	3 [3]	1 [1]	6 [5]
0703800	RNaseII	7	224871	230072	5201	-	-	1 [1]
0703900	VPS46	7	231239	232945	1706	1 [1]	-	-
0704100		7	237410	238876	1466	-	1 [1]	1 [1]
0704200		7	239515	241908	2393	-	5 [2]	7 [3]
0704400		7	248171	250372	2201	-	8 [4]	12 [5]
0704500		7	258699	259922	1223	-	1	1
0704800		7	263509	263867	358	-	-	1
0704900		7	264111	267935	3824	-	1 [1]	3 [1]
0705200	ClpY	7	278348	280858	2510	1	1	-
0705300		7	283361	284702	1341	-	-	1
0705400		7	287593	290256	2663	-	1 [1]	-
0706500		7	349163	357151	7988	-	6 [4]	1 [1]
0707000		7	369366	372335	2969	-	1	-
0707400		7	382837	386811	3974	1	5 [2]	3 [1]
0707600		7	391401	393353	1952	-	3 [1]	8 [1]
0707700		7	398687	401773	3086	1	3 [2]	4 [1]
0707900		7	405279	409415	4136	-	1 [1]	-
0708000		7	409792	413016	3224	-	2 [2]	6 [6]
0708100		7	414321	418741	4420	4	3 [1]	5 [1]
0708200		7	422976	431819	8843	4 [4]	8 [5]	18 [11]
0708300		7	434074	435408	1334	-	-	1
0708400		7	436075	439524	3449	-	-	2 [1]
0708500		7	440567	445069	4502	2 [1]	3 [1]	5 [1]
0708800	NBP35	7	452217	453545	1328	-	1 [1]	1 [1]
0708900		7	453909	455739	1830	-	3 [1]	3 [1]
0709000		7	457856	460753	2897	-	1	8
0709100		7	463932	467262	3330	-	2 [1]	3 [2]
0709200		7	469381	470058	677	-	-	2
0709300	CRMP1	7	471305	481192	9887	8 [7]	4 [4]	10 [7]
0709400		7	481787	485627	3840	-	9 [6]	14 [6]
0709900	ICP	7	497793	499537	1744	-	-	1
0710000		7	501285	505697	4412	-	-	2
0710500		7	520793	524671	3878	-	-	1
0711000		7	532569	534734	2165	-	-	1 [1]
0711100		7	536811	537656	845	-	-	1
0711200		7	538522	540510	1988	-	2	-
0711500		7	546342	549365	3023	-	-	1 [1]
0711600		7	550107	552020	1913	1 [1]	2 [1]	1
0711700		7	554387	554952	565	2 [2]	1 [1]	1 [1]
0711800		7	555818	558743	2925	1	1	1 [1]
0711900		7	559524	561911	2387	-	1 [1]	3 [2]
0712000		7	562485	565610	3125	-	1 [1]	4 [1]
0712100		7	568367	570050	1683	-	1	1 [1]
0712200		7	572927	575224	2297	-	-	1
0712300		7	577998	580042	2044	-	-	1
0713000	NDH2	7	608532	610133	1601	-	2	-
0713300		7	616610	618838	2228	3	-	-
0713400	PFK9	7	620165	624418	4253	-	1	1
0713500		7	627328	629397	2069	-	-	1
0713600		7	630071	630850	779	-	1 [1]	1 [1]
0713700		7	631288	632065	777	-	-	2 [1]
0713800	GLTP	7	633888	634541	653	-	2 [2]	5 [3]
0714000		7	637356	641536	4180	-	1	10 [2]
0714100	TRX3	7	645110	646437	1327	-	1	2
0714200		7	647491	650989	3498	-	3 [2]	2 [1]
0714300		7	651564	653460	1896	-	-	1
0714400		7	655556	661210	5654	-	-	5 [3]
0714500	UFD1	7	662073	664254	2181	2	1	1 [1]
0714600		7	665610	666323	713	-	1	-
0714900		7	676439	677185	746	-	-	1 [1]
0715000		7	677644	683118	5474	-	-	4 [2]
0715400		7	691990	696183	4193	-	-	1

0715600	PRP43	7	699900	702473	2573	-	4 [4]	4 [4]
0715700		7	704304	705365	1061	-	1	1
0716100		7	719709	721726	2017	-	-	2
0716200		7	721799	723873	2074	-	2	7
0716300	EIF3F	7	725121	726252	1131	-	-	2
0716400		7	728262	733856	5594	-	1	4
0716600	RECQ1	7	739685	741652	1967	-	2 [1]	3 [1]
0716800		7	746406	747932	1526	-	-	1
0716900		7	751959	754955	2996	-	1	2
0717200		7	764842	765876	1034	-	4	4
0717400		7	771568	773528	1960	-	1	-
0717600		7	776879	778378	1499	-	1 [1]	2 [1]
0717700		7	779077	779805	728	-	-	2 [1]
0718200		7	802073	804886	2813	-	1	4
0718300		7	806380	807283	903	-	1	3 [2]
0718400		7	808475	813400	4925	-	1 [1]	-
0718500		7	814860	816185	1325	-	-	1
0718800		7	824991	826523	1532	-	2 [1]	1 [1]
0718900	PRP24	7	828282	829815	1533	-	-	2 [2]
0719000		7	830117	831412	1295	-	-	1
0719200		7	835025	842541	7516	-	2 [1]	3 [2]
0719300		7	843927	845684	1757	-	6 [5]	7 [5]
0719400	NifU	7	845908	846956	1048	-	-	1
0719900		7	861489	862262	773	-	-	1
0720000		7	863243	866798	3555	-	1 [1]	2 [1]
0720100		7	868641	874070	5429	-	1	2
0720300		7	879966	882758	2792	-	-	4
0720400		7	883121	886462	3341	-	1	4 [1]
0720500	PGK	7	887714	888964	1250	-	1 [1]	2 [1]
0720700	PRP18	7	893355	895968	2613	-	-	4
0720800		7	897100	909136	12036	-	-	5 [3]
0720900	FabG	7	910715	912144	1429	-	-	1
0721200	CPR	7	917352	919559	2207	-	-	1
0721300	PLP3	7	920448	922802	2354	-	-	4 [1]
0721400		7	923978	927451	3473	-	3 [3]	8 [7]
0721700		7	933789	935142	1353	-	1	2
0721900		7	940707	942236	1529	-	1	2
0722100		7	951609	953402	1793	-	-	1 [1]
0722300	TPK	7	957939	959105	1166	-	-	5 [3]
0722400		7	962765	966117	3352	-	-	2 [1]
0722500		7	966666	970866	4200	-	6 [2]	1
0722600		7	972569	978857	6288	-	-	3 [1]
0722700	SF3A3	7	980191	982047	1856	-	-	4 [3]
0722900		7	985747	987565	1818	-	-	1
0723100	RRP8	7	992491	994087	1596	-	-	1
0723200	aPRS	7	994352	996010	1658	-	1	5 [2]
0723600	HDAC1	7	1005249	1006595	1346	-	-	1
0723700		7	1009482	1013504	4022	-	1	3
0723900		7	1019048	1023224	4176	-	1	9 [3]
0724000		7	1023782	1033348	9566	-	2 [1]	17 [10]
0724700		7	1096572	1100422	3850	-	-	6 [4]
0724800	NADSYN	7	1100899	1103499	2600	-	-	3 [1]
0724900		7	1103563	1107018	3455	-	-	1 [1]
0725100		7	1129649	1130521	872	-	-	3 [1]
0725400		7	1135435	1136016	581	-	-	3 [1]
0725500		7	1142162	1146162	4000	1	-	1
0726600		7	1178549	1180685	2136	-	-	3 [1]
0726700		7	1182175	1183781	1606	-	-	1
0727000		7	1187243	1192537	5294	-	-	3 [1]
0727700		7	1235848	1237730	1882	-	-	2
0727800		7	1239044	1240918	1874	-	-	2 [2]
0727900	RhopH2	7	1242436	1247902	5466	-	3 [1]	19 [3]
0728000		7	1248153	1249577	1424	5 [3]	-	3 [3]
0728100		7	1250641	1251590	949	2 [1]	-	-
0728200		7	1253540	1254751	1211	14 [7]	-	-
0728300		7	1255222	1255518	296	1	-	-
0728400		7	1256722	1257933	1211	17 [1]	1	1
0728600		7	1260894	1264307	3413	7 [3]	2	-

0728700	LRR8	7	1264993	1267530	2537	28 [5]	2	-
0728800	MSP1P	7	1269175	1274799	5624	6 [4]	3 [2]	-
0728900	MSP1	7	1277457	1282922	5465	5 [2]	1 [1]	3 [1]
0729000		7	1283873	1287935	4062	18 [10]	-	4 [2]
0729200	CYP72	7	1294660	1297041	2381	4 [1]	-	-
0729300		7	1298038	1299568	1530	9 [4]	-	1
0729600	NFU1	7	1325810	1326564	754	-	-	1
0729700		7	1327732	1330281	2549	6 [4]	-	2 [1]
0730400		7	1350787	1353278	2491	4	-	5 [1]
0730600	AKLP2	7	1355918	1357423	1505	3	-	1
0730700		7	1359166	1360434	1268	4 [1]	-	2 [1]
0730800		7	1360889	1365076	4187	-	11 [5]	17 [6]
0731000	M18AAP	7	1369431	1371035	1604	1	-	3
0731300	RPS6	7	1377023	1377909	886	-	1	1
0731400		7	1378194	1381262	3068	-	6 [4]	8 [5]
0731500		7	1381735	1385954	4219	1	-	-
0731600		7	1387775	1388293	518	-	1 [1]	-
0731700		7	1389793	1392255	2462	2 [2]	13 [9]	14 [10]
0732000		7	1398967	1401474	2507	2	-	14 [6]
0732200		7	1402855	1406685	3830	-	-	2 [1]
0732300	MAS1	7	1408220	1409623	1403	7	1	-
0732400		7	1411380	1412095	715	3 [1]	2	1 [1]
0732500		7	1412875	1414857	1982	2	1	4 [1]
0732700		7	1417343	1419184	1841	1	-	-
0732800	XPD	7	1420277	1423330	3053	7 [2]	5	2 [1]
0732900		7	1423610	1424047	437	1	-	-
0733100	ApiAP2	7	1426799	1427458	659	2	-	2
0733200		7	1427963	1428888	925	1	-	1
0733300		7	1431109	1432138	1029	1	-	1
0733400		7	1434083	1435711	1628	7 [4]	1 [1]	-
0733500	PKAc	7	1438313	1440058	1745	10	6 [1]	4 [1]
0733600		7	1440851	1442446	1595	11 [4]	3 [1]	-
0733700		7	1443271	1443849	578	2	1	-
0733900	VPS33	7	1445098	1448169	3071	21 [10]	11 [4]	1
0734000		7	1448963	1449791	828	5	-	-
0734100	GDV1	7	1453866	1455914	2048	12 [5]	-	3 [1]
0734200		7	1457274	1458741	1467	23 [14]	2 [1]	3 [2]
0734300		7	1460238	1463096	2858	17 [8]	9 [5]	4 [2]
0734500		7	1472799	1474634	1835	30 [15]	23 [11]	2 [1]
0734600		7	1477509	1478380	871	7 [4]	6 [3]	-
0734800		7	1484769	1485905	1136	6 [3]	3 [2]	3 [2]
0735000		7	1493687	1494967	1280	4 [4]	-	2 [2]
0800400		8	17186	21713	4527	-	-	4 [3]
0800700		8	66778	67873	1095	-	-	3
0800800		8	71092	72993	1901	-	-	1
0801100		8	91027	92340	1313	-	-	2 [1]
0801200		8	95840	98927	3087	-	1	3
0801600		8	109256	113732	4476	1 [1]	1	-
0801700		8	115856	117123	1267	-	2 [1]	-
0801800		8	119160	122618	3458	8 [2]	2 [1]	4 [2]
0801900		8	124340	125326	986	8	-	2
0802000		8	127071	127925	854	5 [3]	-	-
0802400	IMC1c	8	142808	143668	860	2 [1]	2 [1]	1
0802500		8	144942	148619	3677	7 [3]	3 [1]	2
0802600		8	152001	155738	3737	8 [1]	2 [1]	6 [2]
0802800		8	159461	160657	1196	-	1	1
0802900		8	163971	172401	8430	8 [7]	-	3
0803000		8	175319	178570	3251	-	2 [2]	3 [2]
0803100		8	181778	184291	2513	-	4 [3]	4 [3]
0803400		8	196850	198119	1269	-	-	1
0803800	UTP25	8	205385	208615	3230	-	1 [1]	2 [2]
0805600		8	266701	268231	1530	-	2	2
0805700		8	271501	273550	2049	-	1 [1]	1
0805800		8	275059	276926	1867	-	-	2
0806100		8	285604	287597	1993	-	1	1
0806200		8	288755	289399	644	-	-	1 [1]
0806500	ApiAP2	8	293397	297512	4115	-	-	2 [1]
0807000	HDA2	8	320843	327262	6419	-	1 [1]	1 [1]

0807100		8	331332	341729	10397	-	4	11 [5]
0807300		8	345120	346551	1431	-	-	1
0807400		8	347234	348876	1642	-	-	1
0808700		8	414910	415722	812	-	1	1
0809500		8	437528	441313	3785	-	-	3 [1]
0810900		8	490909	493557	2648	-	2 [1]	3 [2]
0811000		8	495135	495950	815	-	1	1
0811100	ARV1	8	496954	498699	1745	-	2 [2]	-
0811400		8	503866	505497	1631	-	1	1
0811600	PREBP	8	513625	516606	2981	-	1	2
0811800		8	521904	525697	3793	-	-	1 [1]
0812000		8	530017	531245	1228	-	-	2 [1]
0812200	HGPRT	8	535571	536738	1167	-	-	1
0812300		8	538845	540626	1781	-	-	1
0812600		8	553559	555721	2162	-	1 [1]	1
0812800		8	560787	562770	1983	-	1 [1]	3 [3]
0812900	UTP13	8	563547	567226	3679	-	-	1
0813000		8	571067	573715	2648	-	1 [1]	1 [1]
0813100		8	574964	576832	1868	-	1	-
0814700	ADA2	8	665731	673353	7622	-	1 [1]	1 [1]
0815300	CysRS	8	699867	702326	2459	-	-	1
0815800		8	714397	716539	2142	-	1 [1]	1 [1]
0817800		8	792950	796807	3857	-	2 [1]	2 [1]
0819000		8	860684	868402	7718	-	1 [1]	1 [1]
0819200		8	878480	880896	2416	-	-	1
0819300		8	881943	884603	2660	-	-	1 [1]
0819400		8	884929	886326	1397	-	-	1 [1]
0819500		8	888542	890866	2324	-	3 [1]	3 [1]
0819700		8	896429	901234	4805	-	2	2
0820500		8	926317	929517	3200	3	-	2 [1]
0822000		8	990492	993035	2543	-	4 [3]	11 [6]
0822500		8	1032425	1037689	5264	-	5 [1]	6 [1]
0822900		8	1047915	1061387	13472	-	2 [2]	3 [3]
0823100		8	1069530	1072799	3269	-	2 [1]	3 [1]
0823400		8	1084300	1086723	2423	-	1 [1]	1 [1]
0823900		8	1107225	1109262	2037	-	-	1
0824100		8	1111027	1119726	8699	-	8 [5]	8 [5]
0824300	EG5	8	1126642	1131027	4385	-	16 [11]	18 [12]
0824500		8	1138363	1147188	8825	-	5 [3]	6 [3]
0824600		8	1151264	1155364	4100	-	2	2
0824700		8	1156116	1158785	2669	-	11 [5]	8 [5]
0824800		8	1160942	1161824	882	-	1	-
0824900		8	1163706	1165369	1663	-	2	2
0825900		8	1220730	1226807	6077	-	1 [1]	5 [3]
0826300		8	1234012	1234983	971	-	3 [2]	3 [2]
0826400		8	1235025	1238327	3302	-	1	1
0826500		8	1239953	1243921	3968	-	1	1
0826900	CTRP	8	1260025	1265580	5555	-	2 [1]	4 [1]
0827400		8	1291546	1295151	3605	-	6 [4]	11 [6]
0828200		8	1316254	1318144	1890	-	-	1 [1]
0828300		8	1320210	1323026	2816	-	7 [4]	7 [4]
0828500		8	1327036	1331078	4042	-	1 [1]	1 [1]
0828700		8	1334635	1337007	2372	-	1	2 [1]
0829100		8	1342768	1345529	2761	-	7 [3]	7 [3]
0829800	GSK3	8	1358586	1360867	2281	-	1	2
0831000	PKRP	8	1418422	1427022	8600	-	1	1
0832200		8	1485691	1491870	6179	-	6 [3]	7 [4]
0832300		8	1492496	1495648	3152	-	3 [2]	4 [3]
0832400		8	1496903	1500942	4039	-	8 [4]	8 [4]
0832600		8	1517736	1520171	2435	-	-	1
0832800		8	1523968	1528419	4451	-	3 [2]	3 [2]
0833200		8	1536196	1538677	2481	-	-	1
0833400		8	1540408	1542789	2381	-	7 [3]	7 [3]
0833500	ARK2	8	1545539	1550399	4860	-	5 [1]	7 [2]
0833600		8	1551048	1552175	1127	-	1	-
0833700	YVH1	8	1552572	1555309	2737	-	1	4
0833800	SF3B1	8	1556262	1560284	4022	-	10 [3]	12 [5]
0834000		8	1562536	1564676	2140	-	3	4 [1]

0834100	PRPF19	8	1565844	1567340	1496	-	1	1
0834200		8	1568760	1569579	819	-	-	2
0834500	CCT7	8	1575515	1577281	1766	-	3 [1]	4 [1]
0834600		8	1577825	1582807	4982	-	3 [2]	4 [2]
0834700		8	1583889	1585322	1433	-	2 [1]	2 [1]
0834800		8	1587097	1598531	11434	-	3	5 [1]
0835000		8	1603370	1609251	5881	-	3 [2]	5 [3]
0835100		8	1609735	1612153	2418	-	1 [1]	1 [1]
0835300	ClpP	8	1615413	1616531	1118	-	1	2 [1]
0835400		8	1617137	1618651	1514	-	2	3
0835700		8	1626480	1627791	1311	-	1	2
0835900	CCT2	8	1630475	1632073	1598	-	1	1
0836300		8	1637363	1639282	1919	-	-	1
0836500	AHA1	8	1642586	1644446	1860	-	1	2
0836600		8	1647309	1649639	2330	-	-	5 [2]
0836700		8	1651156	1651569	413	-	-	1
0836900		8	1653318	1656104	2786	-	-	1
0837000		8	1658522	1659424	902	-	2	1
0837200		8	1664219	1674682	10463	-	13 [3]	13 [5]
0837600		8	1688274	1698799	10525	-	-	2 [2]
0838800		8	1779061	1782735	3674	-	-	2 [2]
0839100	IMC1a	8	1793924	1797031	3107	-	1	5
0839300		8	1799844	1801766	1922	-	1 [1]	1 [1]
0839400	BCKDH-E2	8	1803153	1804505	1352	-	-	1
0839700	DHHC1	8	1813643	1816473	2830	-	1 [1]	2 [1]
0840200		8	1839648	1843701	4053	-	1	3
0840500	ABCB4	8	1852548	1856618	4070	-	1	4 [1]
0840600	SRPK1	8	1858971	1863053	4082	-	1	2
0840700	PRP46	8	1867966	1869964	1998	-	3	3
0841000		8	1879435	1880388	953	-	-	4 [2]
0900200		9	41054	42245	1191	4 [3]	-	-
0900300		9	49868	50830	962	3 [3]	-	-
0900400		9	57907	58971	1064	1 [1]	-	-
0900500		9	64976	67309	2333	1 [1]	-	-
0900600		9	71144	72622	1478	6 [3]	4 [3]	2 [2]
0901100		9	86721	90718	3997	-	2 [1]	1 [1]
0901200	ALP2a	9	92865	94340	1475	1	1	-
0901300		9	96927	97661	734	-	1	-
0901400	NOT1	9	101100	111101	10001	-	-	3 [1]
0901600		9	112524	114816	2292	-	1 [1]	-
0901800	SNF2L	9	120442	125252	4810	-	6 [3]	6 [2]
0902200		9	139726	141615	1889	-	-	1
0902900		9	171730	173960	2230	-	6 [4]	8 [4]
0903200	PTEX88	9	180897	183146	2249	-	8 [5]	1
0903600	RUVB2	9	196657	198084	1427	-	1	1
0903700		9	199486	200879	1393	-	-	1
0903800		9	201681	203945	2264	-	1	-
0903900		9	204099	207207	3108	1	1	-
0904100		9	208862	214585	5723	-	2	2
0904300		9	219608	222718	3110	-	1	1
0904500		9	229629	230224	595	-	1 [1]	1 [1]
0904600	LSM4	9	232026	232871	845	-	-	1
0904800		9	240818	244092	3274	-	-	3
0904900	PAIP1	9	245047	256431	11384	5 [2]	2 [1]	8 [5]
0905000	RAD51	9	261965	263017	1052	-	1 [1]	1 [1]
0905300	PES	9	271974	273770	1796	-	1 [1]	1 [1]
0905400	ApiAP2	9	275541	281449	5908	9 [3]	4 [2]	3 [1]
0905500		9	291748	296619	4871	4 [1]	-	5 [1]
0905600		9	300527	302305	1778	-	3	7 [3]
0905700		9	304623	307372	2749	6 [5]	4 [3]	3 [2]
0906000	CK2alpha	9	311539	312546	1007	1	1	-
0906100		9	314185	315519	1334	-	-	2
0906200	ERC	9	316547	317581	1034	-	-	1
0907200		9	343379	344062	683	-	1	1
0907300		9	344862	345490	628	4	-	2
0907400		9	346841	348973	2132	3	-	4
0908100		9	403366	408064	4698	-	2 [1]	4 [1]
0908200	VPS35	9	410655	414005	3350	-	2	1

0908500		9	418836	420239	1403	-	1 [1]	1 [1]
0908700		9	424250	425962	1712	-	1 [1]	1 [1]
0909300		9	438288	441078	2790	-	-	2 [1]
0909500		9	445081	446653	1572	-	1 [1]	2 [1]
0909600		9	447223	448757	1534	-	-	1
0909800		9	451760	459181	7421	-	4 [1]	4 [1]
0909900	COQ4	9	460565	461632	1067	-	1 [1]	1 [1]
0910300		9	469574	471541	1967	-	1	-
0910400		9	472748	476620	3872	-	-	1
0910500		9	477786	480098	2312	-	-	1
0910600		9	482675	500352	17677	4 [2]	2	16 [4]
0910700	PRL	9	500709	501467	758	-	-	1
0910800		9	506814	507347	533	-	-	1 [1]
0911100		9	514446	516515	2069	-	-	1 [1]
0911900		9	540036	541940	1904	-	5 [5]	8 [6]
0912000		9	542876	543985	1109	-	-	1
0912100		9	545084	546175	1091	-	6 [6]	6 [6]
0912200	CLK3	9	549651	553410	3759	1	-	5
0912300	G3PDH	9	553573	556169	2596	-	1	1
0912400		9	557526	564692	7166	-	1 [1]	6 [2]
0912900		9	580928	582415	1487	-	1	-
0913000		9	584702	586141	1439	-	1	-
0913100	ApiAP2	9	588467	589619	1152	-	-	1
0913700	RON4	9	640262	643136	2874	7 [6]	1 [1]	1
0913800		9	644431	648981	4550	11 [5]	-	2 [1]
0913900	PDX2	9	651532	653164	1632	1	-	2
0914000	CYP19C	9	654579	656142	1563	-	-	2
0914100	SEC12	9	656226	657575	1349	1 [1]	1 [1]	-
0914200	FT2	9	660335	661708	1373	-	1	2
0914300	STT3	9	662232	665010	2778	-	3 [1]	3 [1]
0914400	DPAP1	9	666230	668284	2054	3 [2]	3 [1]	6 [2]
0914500	HSP101	9	671943	675194	3251	-	2	3
0914600		9	679232	681264	2032	-	-	3
0914700		9	682890	686390	3500	-	2 [1]	8 [4]
0914800	UCH54	9	688354	689709	1355	-	-	1 [1]
0915500	MLH	9	710193	712790	2597	-	1	1
0915600		9	713784	718063	4279	-	3 [1]	2 [1]
0915900		9	723773	726361	2588	1	1	-
0916000		9	729362	733402	4040	1	1	-
0916100		9	734100	735011	911	-	-	1
0916200		9	737635	739296	1661	-	-	1
0916700		9	753641	754753	1112	1 [1]	-	-
0916900		9	758235	758831	596	-	1	1
0917100		9	763091	768586	5495	-	1 [1]	4 [3]
0917200		9	769799	771752	1953	-	2 [1]	4 [2]
0917300		9	772604	775462	2858	-	5 [3]	5 [3]
0917400		9	779748	782108	2360	-	-	1
0917600		9	785923	790209	4286	-	4 [2]	6 [4]
0917700		9	792485	796758	4273	-	1	2
0918000		9	803163	804692	1529	-	1	1
0918100		9	805566	806808	1242	1 [1]	1	2 [1]
0918200		9	809098	811655	2557	-	-	4 [2]
0918300		9	811882	820672	8790	-	4 [2]	8 [3]
0918600	HSBP	9	828070	828390	320	-	1	2 [1]
0918700	DHHC3	9	830053	831768	1715	-	-	2
0918800		9	832108	836499	4391	-	1	1
0919000	TKL2	9	839217	843804	4587	-	1 [1]	2 [1]
0920700		9	939668	955440	15772	-	1 [1]	1 [1]
0920800		9	956284	962637	6353	-	3 [2]	2 [1]
0920900	CDPK7	9	969079	974717	5638	-	4 [2]	4 [2]
0921000	LRR11	9	976615	979342	2727	-	-	2 [1]
0921300		9	987637	991425	3788	-	-	1
0921400		9	993854	996076	2222	-	1	3 [1]
0921600	SMC5	9	998757	1002299	3542	-	-	3 [1]
0921700		9	1003863	1004634	771	-	-	1
0921800	ERO1	9	1006969	1008913	1944	1 [1]	3 [1]	3
0921900		9	1009921	1014333	4412	-	-	3 [2]
0922000		9	1015483	1017148	1665	-	6 [2]	5 [2]

0922200	LSM1	9	1026152	1027020	868	-	1	1
0922700		9	1043596	1048383	4787	-	-	2 [2]
0923100		9	1057708	1059175	1467	-	1	1
0923200	mtRNAP	9	1061436	1065482	4046	-	2	-
0923500		9	1071775	1072263	488	-	1	-
0923600		9	1073427	1075845	2418	-	2 [1]	3 [1]
0923700		9	1077079	1079528	2449	-	1 [1]	2 [1]
0923900	ThrRS	9	1083113	1085779	2666	-	-	1
0924000	ATG7	9	1087050	1090664	3614	1	-	1
0924200		9	1095511	1098146	2635	-	-	1 [1]
0924300		9	1099135	1100450	1315	-	-	1
0924400		9	1101951	1105814	3863	-	2 [2]	4 [2]
0924500		9	1106661	1108493	1832	-	1 [1]	1 [1]
0924800		9	1135136	1137976	2840	-	-	2 [1]
0925000		9	1140925	1142768	1843	1	4 [2]	-
0925500		9	1152953	1156361	3408	-	-	3
0925600		9	1158272	1160648	2376	-	-	1
0925700		9	1161153	1162829	1676	-	-	1
0925800		9	1163773	1167858	4085	1 [1]	-	3 [2]
0925900		9	1170078	1171804	1726	1	-	-
0926000		9	1174296	1181583	7287	-	3 [1]	3 [1]
0926200		9	1186973	1188040	1067	-	1 [1]	-
0926400		9	1190783	1195388	4605	-	-	1
0926500	GGPPS	9	1196684	1199540	2856	-	-	1
0926900		9	1210116	1212582	2466	-	-	3
0927000		9	1214086	1215592	1506	-	1	-
0927100	PV1	9	1218278	1219564	1286	3 [3]	2 [2]	-
0927200	RPN7	9	1222023	1223819	1796	-	-	1
0927300		9	1224471	1229558	5087	-	-	1
0927600		9	1237912	1241022	3110	1	-	1 [1]
0927800		9	1246242	1247999	1757	4 [2]	-	5 [3]
0928100		9	1256005	1256949	944	1 [1]	-	1 [1]
0928500	RPT5	9	1260265	1261587	1322	-	1	1
0928600		9	1263016	1267073	4057	-	1 [1]	2 [1]
0928700		9	1267727	1271051	3324	-	-	1
0928800	SMC1	9	1271205	1276067	4862	-	7 [1]	7 [1]
0928900		9	1278197	1279919	1722	-	2	2
0929200		9	1286688	1288793	2105	-	2 [2]	2 [2]
0930200		9	1340312	1342750	2438	-	1 [1]	1 [1]
0930300		9	1343312	1346170	2858	-	2 [1]	6 [5]
0930400	TCP1	9	1348705	1350508	1803	-	-	2
0930600		9	1355323	1358916	3593	2 [2]	4 [3]	5 [4]
0930700		9	1360823	1365256	4433	8 [5]	-	10 [7]
0931200		9	1377312	1380862	3550	2 [1]	-	4 [2]
0931300		9	1381519	1385803	4284	5 [2]	1	3 [2]
0931400		9	1389092	1390189	1097	4 [1]	-	-
0931700		9	1398532	1400328	1796	1 [1]	-	1 [1]
0931800		9	1401288	1403633	2345	3 [2]	-	5 [1]
0931900		9	1405590	1408215	2625	1 [1]	-	1 [1]
0932300		9	1438989	1440263	1274	1	-	2
0932400		9	1443220	1448730	5510	-	2	2
0932600		9	1457234	1458550	1316	-	-	1
0932700		9	1460268	1465153	4885	-	-	1
0932800		9	1466863	1472685	5822	-	-	1 [1]
0932900	RPA2	9	1478201	1482964	4763	3 [1]	2 [1]	2 [1]
0933000		9	1484824	1486377	1553	2	1	-
0933100		9	1487457	1489457	2000	11 [6]	-	9 [5]
0933200	PPM8	9	1490463	1493360	2897	7 [4]	6 [4]	3 [2]
0933300		9	1495816	1498913	3097	1	-	4 [3]
0933400		9	1499957	1501186	1229	3 [2]	-	2 [1]
0933500		9	1503223	1504011	788	-	-	2 [1]
0933600		9	1504998	1505849	851	-	-	4 [2]
0933700		9	1507158	1509023	1865	3 [1]	-	2
0933800		9	1510110	1516089	5979	2	1	8 [2]
0933900		9	1516212	1516628	416	-	2	2
0934100		9	1519141	1521626	2485	-	-	5
0934200		9	1525562	1535060	9498	-	10 [4]	23 [12]
0934300		9	1536058	1537788	1730	-	1 [1]	2 [2]

0934400		9	1538104	1540979	2875	-	-	1
0934500	TSN	9	1544069	1547506	3437	3 [1]	2 [1]	2 [1]
0934600	SRP72	9	1547927	1550437	2510	1	4	4
0934700	CK1	9	1553624	1556012	2388	1	-	5
0934800		9	1563469	1566399	2930	-	6 [2]	8 [4]
0934900		9	1567417	1569647	2230	7	-	3
0935000		9	1571258	1573032	1774	5	-	2
0935100		9	1578402	1582649	4247	10 [6]	2 [1]	6 [1]
0935300		9	1585559	1586470	911	1	-	2 [1]
0935400		9	1587112	1588419	1307	-	1	1
0935500		9	1590105	1592054	1949	1	-	1
0935600		9	1593800	1595098	1298	-	-	1
0935700		9	1596818	1597478	660	2 [1]	-	-
0935800		9	1598619	1600123	1504	-	-	1 [1]
0935900		9	1600897	1603008	2111	-	-	1 [1]
0936000		9	1604214	1604864	650	-	-	1
0936200		9	1611246	1620080	8834	1 [1]	-	3 [3]
0936400		9	1622383	1623119	736	1	1 [1]	1 [1]
0936700	PPM2	9	1642625	1645207	2582	-	-	1 [1]
0936900		9	1650368	1656466	6098	-	-	2 [1]
0937000		9	1658439	1664688	6249	-	2 [1]	3 [1]
0937100		9	1673320	1678227	4907	-	4 [3]	12 [8]
0937200		9	1680393	1681866	1473	-	-	4
0937300	ApiAP2	9	1683321	1691821	8500	-	1	4 [2]
0937400		9	1702900	1705683	2783	-	-	1
0938300		9	1736385	1742371	5986	-	-	4 [1]
0938400		9	1742639	1749427	6788	-	8 [4]	23 [14]
0938500		9	1751513	1759030	7517	-	6 [4]	17 [6]
0938600		9	1764557	1767112	2555	-	-	2 [1]
0938700		9	1767804	1773894	6090	-	-	1 [1]
0938800		9	1776013	1785042	9029	-	-	3 [2]
0938900		9	1786447	1793916	7469	-	1 [1]	1 [1]
0939000		9	1794648	1795115	467	-	-	1
0939300		9	1802795	1805381	2586	1	-	2 [1]
0939500		9	1807632	1809406	1774	-	-	1
0939600		9	1810223	1812391	2168	-	-	1
0941000	AP2-O	9	1880715	1886036	5321	-	1 [1]	2 [2]
0941700		9	1909465	1911933	2468	-	-	1
0942200		9	1920308	1920771	463	-	-	1
0942500	TFG2	9	1932576	1933667	1091	-	1 [1]	-
0943000	SEC21	9	1957639	1960638	2999	-	1	1
0943100		9	1962987	1968740	5753	-	1	9 [4]
0943700		9	1990917	1993970	3053	-	-	3 [2]
0943800		9	1995294	1997871	2577	-	-	2
0943900	RSA4	9	1998229	2000148	1919	-	1	2 [1]
0944200		9	2006080	2007384	1304	-	-	1 [1]
0944600		9	2022344	2031229	8885	1 [1]	2 [1]	5 [2]
0944700		9	2033923	2035559	1636	1	-	1
0944800	SLARP	9	2036603	2046516	9913	2 [2]	2 [2]	13 [10]
0945000		9	2054676	2063225	8549	1 [1]	-	2 [2]
0945700	MAEBL	9	2089784	2096346	6562	2 [1]	-	3 [1]
0946000		9	2106458	2107390	932	4 [2]	2	4 [2]
0946100		9	2112725	2122206	9481	15 [12]	5 [4]	11 [9]
0946200		9	2133872	2137586	3714	15 [12]	5 [5]	1 [1]
0946300		9	2146753	2149951	3198	2	-	1
1000700		10	48470	51298	2828	1 [1]	1 [1]	1 [1]
1001800		10	100871	108514	7643	-	1 [1]	1 [1]
1002900		10	170876	172214	1338	-	1 [1]	1 [1]
1003000		10	173895	179363	5468	-	1	-
1004500	EIF3E	10	229100	230947	1847	-	1	1
1007500		10	346608	348023	1415	-	-	1
1007800		10	363468	366110	2642	2 [2]	6 [3]	12 [6]
1008800		10	400438	404010	3572	20 [8]	-	9 [4]
1008900		10	406597	409971	3374	6	1	4
1009000		10	415449	416469	1020	9 [4]	-	3
1009100		10	418288	420420	2132	7 [4]	3 [2]	-
1009300		10	430692	432060	1368	6	-	2
1009500		10	434984	436943	1959	-	1 [1]	2 [1]

1009600		10	439732	440453	721	-	-	1
1009800		10	445588	447467	1879	1	-	1
1009900	MDR1	10	448974	453368	4394	-	-	2 [2]
1010000		10	456866	462649	5783	-	2 [1]	11 [7]
1010300		10	470593	472044	1451	-	-	1
1010500		10	476464	503462	26998	4 [3]	6 [4]	13 [9]
1010700	TAF10	10	509334	510251	917	-	2	3
1010800		10	512109	514163	2054	-	1 [1]	-
1010900		10	514645	515950	1305	-	-	5
1011000		10	516948	522795	5847	-	-	2
1012900		10	585235	587340	2105	-	1 [1]	-
1013200		10	596846	598252	1406	-	1	1
1013600	CCR4	10	620833	628489	7656	-	3 [2]	3 [2]
1015300		10	714097	715917	1820	-	1 [1]	-
1015600	EIF3B	10	724456	726606	2150	-	-	1
1015700	CPbeta	10	729181	730095	914	-	1 [1]	1 [1]
1015800		10	731608	733890	2282	-	3	3
1015900	FACT-L	10	735369	738810	3441	-	4	4
1016100		10	746600	748411	1811	-	-	1 [1]
1016200		10	749369	750898	1529	-	4 [3]	4 [3]
1016300		10	751812	752874	1062	-	1	-
1016500	ApiAP2	10	762167	768982	6815	-	13 [9]	12 [8]
1016600		10	770289	774700	4411	-	2 [2]	2 [2]
1016900	PhLP3	10	786278	787279	1001	-	2 [1]	2 [1]
1017300	NIF2	10	799608	800591	983	-	1	-
1017500	GAP40	10	805608	806999	1391	-	1	1
1017600		10	808337	813390	5053	-	3	4
1017700		10	815915	818908	2993	-	2 [2]	2 [2]
1018800		10	870730	873666	2936	-	3 [2]	3 [3]
1018900		10	874545	876770	2225	-	1	1 [1]
1019000	UvrD	10	879983	882913	2930	-	3	3
1019100		10	883356	885507	2151	-	3 [2]	3 [2]
1019400	PSOP12	10	889571	891904	2333	-	-	1 [1]
1019900		10	904986	912072	7086	-	5 [3]	5 [3]
1020000		10	913669	914656	987	-	1	-
1022300		10	1026151	1030322	4171	-	1	1
1022600	TCTP	10	1037839	1038354	515	-	1 [1]	-
1023100		10	1058188	1060962	2774	-	1	2
1023400	CYP87	10	1066801	1068972	2171	-	1	2
1023500		10	1076261	1082719	6458	2 [2]	8 [5]	19 [10]
1023600		10	1083674	1086695	3021	-	2	2
1023700		10	1087384	1088457	1073	-	1 [1]	2 [1]
1023800	PI4K	10	1089268	1094412	5144	-	2 [1]	5 [2]
1023900		10	1096588	1098733	2145	-	-	1
1024000		10	1098867	1101050	2183	-	5 [3]	5 [4]
1024200		10	1104927	1113026	8099	-	13 [3]	10 [3]
1024400	LRR2	10	1115297	1116901	1604	-	1	1
1024500		10	1117410	1121867	4457	-	3 [1]	2 [1]
1024700		10	1127371	1135647	8276	-	5 [3]	6 [5]
1025100		10	1148004	1154651	6647	-	4 [3]	1 [1]
1025400		10	1163504	1165053	1549	-	-	1 [1]
1025500		10	1165580	1170118	4538	-	2 [1]	2 [1]
1025600	P38	10	1173002	1174156	1154	-	2 [1]	1
1025800		10	1175983	1179573	3590	-	1 [1]	3 [3]
1025900	NPL4	10	1183391	1184962	1571	-	-	1
1026000	CAF40	10	1185849	1188272	2423	-	2 [1]	3 [2]
1026100	SUB1	10	1191917	1193833	1916	-	3 [1]	5 [1]
1026300		10	1196486	1199407	2921	1	-	3
1026400	SUB3	10	1201127	1202917	1790	1 [1]	-	2 [2]
1026500	RPL4	10	1205241	1206476	1235	1 [1]	-	-
1026600		10	1207474	1208075	601	-	5 [1]	3 [1]
1026700	ROM4	10	1209628	1211622	1994	1 [1]	1	2 [1]
1026900		10	1216794	1218851	2057	-	-	1
1027400	TBP	10	1237574	1238326	752	-	-	1
1027800	SUMO	10	1245883	1246379	496	-	-	2
1027900		10	1248097	1249557	1460	-	1 [1]	1 [1]
1028000		10	1250405	1251298	893	-	1	-
1028100	MSH6	10	1253574	1257485	3911	-	1	1 [1]

1028200		10	1258960	1261104	2144	-	2	-
1028600		10	1278004	1285463	7459	1 [1]	1 [1]	-
1028900	CEP120	10	1304887	1310034	5147	-	-	1 [1]
1029300		10	1320978	1322080	1102	-	-	1
1029600	ATPase3	10	1327931	1334157	6226	-	3 [3]	2 [2]
1030300	SRSF12	10	1383454	1384437	983	-	-	2
1030400		10	1386557	1387276	719	1 [1]	3 [2]	5 [4]
1030700		10	1396379	1398970	2591	-	2 [1]	2 [1]
1031200		10	1409040	1410282	1242	-	1 [1]	1 [1]
1031400		10	1415028	1416533	1505	1 [1]	1 [1]	1
1031500	MSP8	10	1419725	1421155	1430	1 [1]	-	-
1031600		10	1423751	1424791	1040	-	-	1
1032000		10	1435858	1437307	1449	-	-	3 [1]
1032100		10	1437695	1440631	2936	1 [1]	1 [1]	2 [2]
1032200		10	1443730	1445991	2261	1	2 [1]	2 [1]
1032300	RAP2	10	1448208	1449410	1202	11 [9]	7 [6]	2 [1]
1032400	SBP1	10	1452572	1453357	785	3 [2]	1 [1]	2 [1]
1032500		10	1457121	1458187	1066	3 [2]	-	3 [2]
1032600		10	1461837	1467334	5497	7 [5]	-	-
1101700		11	72666	76165	3499	-	1	1
1102000		11	89227	95502	6275	-	3 [2]	4 [2]
1102200	GPAA1	11	99273	102239	2966	-	1	1
1102400	ERCC4	11	108470	113431	4961	-	1	-
1102900		11	124992	126883	1891	-	3 [3]	3 [3]
1103000	ABCE1	11	128971	130944	1973	-	5 [2]	4 [2]
1103400	PSOP2	11	138605	141595	2990	-	1 [1]	1 [1]
1103500	AlaRS	11	143381	146923	3542	-	1	2
1105000		11	214842	227894	13052	-	9 [5]	10 [6]
1105500		11	236966	238525	1559	-	1 [1]	1 [1]
1105900		11	250753	252363	1610	-	2 [1]	2 [1]
1106000		11	253891	256116	2225	-	8 [7]	9 [6]
1106600		11	266579	267685	1106	-	2 [1]	2 [1]
1106900		11	275037	284969	9932	-	1 [1]	1 [1]
1107100		11	292955	298426	5471	-	3 [2]	3 [2]
1107300		11	303477	305990	2513	-	1	1
1107800		11	322073	324668	2595	-	1	1
1108200		11	332988	336399	3411	-	1	1
1108400		11	340915	345485	4570	-	2 [1]	2 [1]
1108500		11	346440	352794	6354	-	2 [1]	2 [1]
1108600		11	354840	359027	4187	-	1 [1]	2 [2]
1110300	SEC24A	11	437138	440816	3678	-	1	1
1110400	PRMT5	11	444333	446390	2057	-	1 [1]	-
1110700	PIAS	11	461192	465895	4703	-	3 [2]	3 [2]
1110800		11	468170	479813	11643	-	5 [2]	5 [2]
1111400		11	492596	498337	5741	-	2	1
1111700		11	514190	520150	5960	-	3 [2]	3 [2]
1111900	CELF1	11	525852	527544	1692	-	1	1
1112300		11	542158	551040	8882	-	3 [2]	4 [3]
1112900		11	608113	610887	2774	-	6 [1]	8 [1]
1114000		11	648997	651624	2627	-	2 [2]	2 [2]
1114100		11	653022	655958	2936	-	1 [1]	-
1114700	ARK3	11	671071	682689	11618	-	2 [1]	2 [1]
1115100		11	694720	697815	3095	-	1 [1]	1 [1]
1115400		11	708194	710995	2801	-	1	2 [1]
1117600	IP5P	11	823875	826277	2402	-	1	1
1118200	TFB4	11	841109	842080	971	-	3 [1]	3 [1]
1118300		11	843270	846704	3434	-	1	1
1118500		11	854354	855199	845	-	-	1
1118700		11	860714	863442	2728	-	1 [1]	1 [1]
1118800		11	865052	866896	1844	-	-	1
1118900		11	868389	871584	3195	-	3 [2]	4 [3]
1119200		11	882478	891593	9115	-	4 [2]	1 [1]
1119500		11	898407	900727	2320	-	2	4
1119600		11	901203	902575	1372	-	2 [1]	3 [1]
1119700		11	905725	909193	3468	-	2 [2]	2 [2]
1119800		11	909970	930148	20178	-	2 [1]	1 [1]
1121500	CEPT	11	1035652	1038460	2808	-	-	2
1121700		11	1054830	1082351	27521	-	6 [5]	6 [5]

1122000	ACS	11	1088206	1091172	2966	-	1 [1]	1 [1]
1122500	RNF5	11	1109289	1110830	1541	-	1 [1]	1 [1]
1123000		11	1127570	1129899	2329	-	1	1
1123100		11	1133623	1134738	1115	-	-	2 [2]
1123300		11	1136876	1139620	2744	-	-	1
1123400		11	1141411	1144269	2858	-	1	1
1123500	FabB/FabF	11	1146913	1149097	2184	-	1	1
1123700		11	1151527	1154045	2518	-	-	5
1123800		11	1155819	1160414	4595	-	2 [2]	4 [3]
1123900		11	1161456	1162509	1053	-	-	2
1124000		11	1164105	1165940	1835	-	-	1
1124500		11	1178590	1182327	3737	-	-	1
1125300		11	1210254	1213181	2927	-	-	1
1125400		11	1215284	1219825	4541	-	2 [1]	7 [4]
1125600	ISWI	11	1223726	1232344	8618	-	2 [2]	5 [4]
1125800		11	1241023	1242780	1757	-	-	2
1126400	TKL4	11	1263196	1269243	6047	-	4 [2]	6 [2]
1126500		11	1271016	1273835	2819	-	1	2
1127100	NAB2	11	1291067	1295640	4573	-	6 [4]	8 [5]
1127300	ApiAP2	11	1309455	1317632	8177	-	4 [3]	8 [3]
1127400		11	1319589	1323476	3887	-	1	1
1127500		11	1325852	1327389	1537	-	1 [1]	1 [1]
1127900		11	1332771	1335662	2891	-	1 [1]	1 [1]
1128000		11	1337499	1339751	2252	-	1 [1]	1 [1]
1128100		11	1341330	1345487	4157	-	2 [2]	4 [3]
1128500		11	1354907	1360200	5293	-	2	1 [1]
1128800	ALV7	11	1364343	1366199	1856	-	4	4
1128900		11	1369194	1371272	2078	-	-	1
1129700		11	1388746	1390323	1577	-	-	2 [2]
1130500		11	1432477	1439337	6860	-	-	2 [2]
1130600		11	1440360	1442733	2373	-	5 [3]	6 [4]
1130700		11	1442897	1445143	2246	-	-	1 [1]
1130800		11	1445994	1450007	4013	-	-	5 [2]
1130900		11	1451470	1452471	1001	-	2 [1]	3 [2]
1131100		11	1459032	1468907	9875	-	-	1 [1]
1131400		11	1476486	1482818	6332	-	-	2
1131600		11	1487462	1490459	2997	-	1	-
1131800	ROM10	11	1493063	1494961	1898	-	2 [1]	2 [1]
1131900	MDH	11	1495470	1496411	941	-	1	1
1132100		11	1502077	1502655	578	-	-	1
1132300		11	1506986	1508920	1934	-	6 [4]	9 [5]
1132400		11	1509911	1512292	2381	-	5 [3]	5 [4]
1132800		11	1526986	1528775	1789	2	-	1
1132900		11	1532064	1536616	4552	-	1	-
1133000		11	1540162	1541346	1184	-	3 [1]	1
1133100		11	1542201	1546151	3950	-	3 [3]	4 [3]
1133200	MQO	11	1547818	1549392	1574	-	-	1
1133300		11	1552219	1553541	1322	-	-	1
1133400		11	1554369	1556234	1865	1 [1]	1 [1]	-
1133500	TLP	11	1556902	1560717	3815	6 [3]	1 [1]	1
1133600		11	1563433	1567257	3824	-	-	2 [1]
1133800	NDC80	11	1569045	1571812	2767	-	1	3
1134100		11	1577816	1581931	4115	-	-	2
1134200		11	1583003	1586408	3405	-	1	2 [1]
1134400		11	1589105	1597003	7898	-	2 [2]	5 [4]
1134500	CRK5	11	1597635	1599869	2234	-	2	1
1134600		11	1603204	1612548	9344	-	15 [13]	13 [11]
1134800		11	1615701	1617803	2102	-	-	2
1135100		11	1621863	1625417	3554	-	1 [1]	-
1135500		11	1635154	1636232	1078	-	-	2
1135700		11	1641418	1644265	2847	-	4 [1]	10 [4]
1135800	NAR1	11	1646105	1648507	2402	-	2 [1]	8 [4]
1135900		11	1649576	1652179	2603	-	7 [5]	12 [8]
1136000		11	1652607	1654328	1721	-	-	6 [4]
1136100		11	1655677	1661304	5627	-	1	11 [2]
1136300		11	1679667	1681607	1940	2	-	-
1136400		11	1682210	1685597	3387	2	1	1 [1]
1136700	ROP14	11	1695037	1698860	3823	-	5 [1]	4

1137200	P12p	11	1711587	1712819	1232	4 [1]	-	1 [1]
1137300	P12	11	1713941	1714984	1043	6 [3]	1 [1]	4 [3]
1137800	LRR6	11	1721014	1726726	5712	1	2	-
1137900	EIF3L	11	1728754	1730535	1781	-	3 [1]	3 [1]
1138300		11	1739179	1749072	9893	-	-	1 [1]
1139500	SPT5	11	1834629	1838378	3749	-	1	1
1140100	SLU7	11	1854468	1856123	1655	-	1	-
1140200		11	1856479	1857766	1287	-	1	1
1140900		11	1926261	1928785	2524	-	2 [2]	1 [1]
1141100		11	1933585	1935692	2107	3 [2]	-	-
1141200		11	1936314	1953317	17003	3	11 [2]	8 [4]
1141500	CCT6	11	1963732	1965487	1755	-	2	2
1142300	DPH7	11	1985705	1988314	2609	-	-	1
1142700	SAS6	11	2007522	2010418	2896	-	6 [1]	11 [1]
1142800	COQ2	11	2010662	2012074	1412	-	3 [2]	5 [2]
1142900		11	2013112	2017035	3923	-	2	1
1143000	UROD	11	2017542	2019012	1470	-	-	1
1143100		11	2020098	2021437	1339	-	3 [1]	2 [1]
1143400	GLP2	11	2031856	2032515	659	-	-	1
1143500		11	2033853	2038534	4681	-	4 [1]	5 [1]
1143600		11	2042552	2048612	6060	-	2	1 [1]
1144100		11	2061199	2063381	2182	-	1 [1]	1 [1]
1144200		11	2068060	2071488	3428	-	1	1
1144300		11	2072604	2074127	1523	-	2 [1]	2 [1]
1145400		11	2135657	2137927	2270	-	2 [1]	2 [1]
1146000		11	2156067	2164565	8498	-	4 [2]	4 [2]
1146400	SIP2	11	2178783	2184398	5615	-	-	1
1147100		11	2223665	2230418	6753	-	2 [2]	1 [1]
1147200	CAX	11	2231526	2232851	1325	-	-	1 [1]
1147300	TEX1	11	2236079	2238826	2747	-	1	-
1147400	DHODH	11	2240514	2242184	1670	-	1	1
1147600		11	2247418	2248836	1418	-	6 [4]	3 [3]
1147700		11	2249741	2251309	1568	-	1 [1]	1 [1]
1147900		11	2256170	2258044	1874	-	1 [1]	-
1148100		11	2262939	2267489	4550	-	1 [1]	-
1148400	PALM	11	2274265	2275191	926	1 [1]	-	1 [1]
1148500		11	2276350	2277418	1068	-	1	1
1148600		11	2279055	2283218	4163	1	4 [3]	3 [3]
1148700		11	2289680	2293516	3836	-	3 [2]	2 [1]
1148800		11	2297540	2299766	2226	4 [2]	7 [2]	3
1148900		11	2302414	2302833	419	4 [2]	-	3 [1]
1149000		11	2309796	2310815	1019	11 [7]	1 [1]	2 [1]
1149100		11	2313991	2314696	705	1 [1]	1 [1]	-
1149400		11	2328521	2329654	1133	12 [7]	2 [2]	1 [1]
1149500		11	2331945	2332833	888	1	-	-
1149600		11	2336944	2338109	1165	14 [10]	-	2 [1]
1201900		12	104434	106089	1655	-	1 [1]	-
1202200		12	131088	134047	2959	-	1 [1]	1 [1]
1202300		12	135489	137235	1746	-	-	2
1202500		12	139029	140606	1577	-	1	1
1202800		12	145916	148540	2624	-	3 [1]	3 [1]
1203000		12	161809	167646	5837	1 [1]	7 [3]	4 [2]
1203700		12	192328	193632	1304	-	1 [1]	1 [1]
1204000	DHFS-FPGS	12	199281	201182	1901	-	2 [1]	2 [1]
1204100		12	201903	202703	800	-	1	1
1204200		12	204080	207314	3234	-	3 [2]	3 [2]
1204300		12	208046	212178	4132	-	1	5 [1]
1204500		12	215468	228681	13213	-	5 [3]	7 [4]
1204700		12	232079	233641	1562	-	1 [1]	2 [2]
1204800		12	238220	241618	3398	-	3 [3]	3 [3]
1204900		12	241784	242992	1208	-	2 [1]	-
1205000	VPS52	12	243762	247052	3290	-	3 [1]	2
1205300	PMV	12	254366	255967	1601	-	-	1
1205600		12	262818	263585	767	-	1	1
1205900		12	268983	271483	2500	-	-	1
1206100		12	274699	275775	1076	-	-	1 [1]
1206600		12	291357	303210	11853	-	17 [14]	18 [13]
1206700	SETvs	12	305888	314936	9048	1 [1]	12 [9]	21 [13]

1207100	SF1	12	332586	335681	3095	-	1 [1]	1 [1]
1207700		12	354391	357788	3397	-	1 [1]	1 [1]
1207800		12	358890	359831	941	-	-	1
1207900		12	362734	364616	1882	-	-	1
1208300		12	371414	373831	2417	-	-	1 [1]
1208600		12	379476	383040	3564	-	1	-
1208800	HDA1	12	387674	392395	4721	-	1 [1]	1 [1]
1210300		12	446085	447833	1748	-	-	2
1210600	MPC2	12	457685	458722	1037	-	-	1
1210900		12	462108	468699	6591	-	1 [1]	-
1211300		12	473569	474213	644	-	1 [1]	1 [1]
1211400	ACC	12	475994	485019	9025	-	-	1 [1]
1211500		12	487808	490057	2249	-	2 [2]	6 [4]
1211600	NT3	12	490742	492094	1352	-	1 [1]	2 [1]
1211800	SHLP1	12	497079	498266	1187	-	1	1
1212300		12	520368	522782	2414	-	1	1
1212900	LRR14.1	12	553111	554217	1106	-	1 [1]	1 [1]
1213100		12	562759	571791	9032	-	11 [2]	20 [4]
1213300		12	581694	586739	5045	-	2 [1]	2 [1]
1214300		12	616303	618816	2513	-	1 [1]	2 [2]
1215100	ApiAP2	12	650618	653902	3284	-	9 [7]	10 [7]
1215200	RPN2	12	655714	659917	4203	-	1 [1]	1 [1]
1215300		12	664292	671002	6710	-	1 [1]	6 [4]
1215400	PPKL	12	672619	675440	2821	-	3 [1]	3 [2]
1215500		12	677627	680230	2603	-	-	2
1215600		12	681279	682681	1402	-	-	3
1215700		12	684122	704235	20113	-	5 [1]	5 [2]
1215800		12	706081	708861	2780	-	1	1
1215900		12	709278	711960	2682	-	1	1
1216100		12	721580	722178	598	-	-	2
1216200		12	722715	727136	4421	-	-	1
1216300		12	728675	729802	1127	-	1	1
1216700		12	741446	742503	1057	-	1	-
1216800		12	744915	746063	1148	-	1	1
1216900		12	747756	751904	4148	-	3 [2]	3 [2]
1217000		12	754019	763391	9372	-	-	2 [1]
1217200		12	766240	769067	2827	-	-	1
1217400		12	772119	774584	2465	-	-	2 [1]
1217500		12	775806	777797	1991	-	-	1 [1]
1217600		12	780210	786018	5808	-	-	5 [1]
1217800		12	788087	789067	980	-	1	2 [1]
1217900		12	790246	790669	423	-	-	2
1218000		12	791388	792822	1434	-	-	1
1218400		12	804412	823447	19035	-	-	10 [7]
1219100		12	854107	866349	12242	-	-	2 [1]
1220100	SF3B2	12	937575	939949	2374	-	-	1
1220300		12	943545	945133	1588	-	-	1
1220600		12	949726	952070	2344	-	2 [2]	3 [3]
1220700		12	952814	954908	2094	-	1 [1]	-
1220800		12	955587	957072	1485	-	-	1
1221200		12	966346	970734	4388	-	3 [2]	5 [4]
1221600		12	980884	982818	1934	-	-	4 [1]
1222200		12	996931	999714	2783	-	1 [1]	9 [3]
1222300		12	1001417	1004275	2858	-	-	1 [1]
1222400		12	1006198	1007220	1022	2	4	7 [1]
1222600		12	1010464	1012800	2336	-	3	4
1222700		12	1013184	1015658	2474	-	1 [1]	1 [1]
1222800		12	1016865	1019390	2525	-	2 [2]	2 [2]
1223000		12	1023982	1026234	2252	-	1 [1]	-
1223300	SAS4	12	1034916	1039804	4888	-	-	9 [6]
1223500		12	1043351	1048174	4823	-	6 [3]	13 [7]
1223600		12	1048375	1048910	535	-	-	1
1224000		12	1067102	1068529	1427	-	1 [1]	2 [2]
1224100		12	1069066	1073565	4499	-	4 [1]	9 [4]
1224200		12	1075857	1077644	1787	-	2 [2]	2 [2]
1224500		12	1093286	1095115	1829	-	3 [1]	10 [5]
1224700		12	1099186	1101696	2510	-	-	3 [1]
1224800	SPP	12	1103599	1106399	2800	-	-	1

1225100		12	1118096	1120228	2132	-	-	1
1225300		12	1123261	1130418	7157	-	-	2
1225400		12	1132739	1135797	3058	-	3 [2]	2 [2]
1225500		12	1136792	1138051	1259	-	1	1
1225600		12	1138288	1140909	2621	-	1 [1]	2 [2]
1225800	ApiAP2	12	1144464	1146790	2326	-	-	3
1225900		12	1150433	1152446	2013	-	-	1
1226000	CCp2	12	1153149	1158113	4964	-	2 [1]	4 [2]
1226100		12	1160753	1164565	3812	-	4 [3]	8 [5]
1226200		12	1167108	1172753	5645	-	-	8
1226300		12	1173709	1177135	3426	-	1 [1]	-
1226400		12	1181399	1183038	1639	-	6 [2]	10 [2]
1226600	HlyIII	12	1206179	1207179	1000	-	3	3
1226700		12	1208560	1210383	1823	-	-	9 [3]
1226800		12	1213434	1215032	1598	-	-	3
1226900	PTP1	12	1218252	1219170	918	-	2	4
1227000		12	1220631	1222675	2044	-	1	5 [1]
1227700	APP	12	1239487	1241901	2414	-	1	2
1227800	KIN	12	1244316	1246820	2504	-	4 [1]	10 [5]
1228000		12	1254073	1254716	643	-	-	1
1228200		12	1258763	1261342	2579	-	1 [1]	1 [1]
1228300	GluPho	12	1262887	1265631	2744	-	8 [5]	2
1228400	P23	12	1266855	1268340	1485	-	1 [1]	1 [1]
1228500		12	1271688	1276765	5077	-	1 [1]	4 [3]
1228900		12	1289787	1297286	7499	-	1	2
1229000		12	1298154	1300139	1985	-	5 [3]	6 [4]
1229100		12	1301292	1303862	2570	-	1	1
1229400		12	1307515	1309910	2395	-	3 [1]	4 [1]
1229700		12	1325775	1328780	3005	-	3 [2]	6 [2]
1229800		12	1331458	1332525	1067	-	-	1
1229900		12	1332787	1334382	1595	-	-	1
1230100	RON2	12	1337456	1343887	6431	28 [20]	3 [2]	16 [6]
1230200	TSR1	12	1347341	1350316	2975	3 [1]	-	4 [1]
1230300		12	1351998	1355273	3275	-	1	7
1230400	CNB	12	1358605	1360045	1440	3	-	5
1230500	LAP5	12	1361368	1364091	2723	-	1	3 [1]
1230600		12	1365388	1365852	464	-	-	2 [1]
1230700	Sir2b	12	1366485	1370198	3713	-	-	5 [2]
1230800	NSE2	12	1372057	1374592	2535	-	4	12
1230900		12	1378988	1383850	4862	-	-	5 [4]
1231000	eEF2	12	1385314	1387812	2498	-	-	1
1231100		12	1392093	1394363	2270	-	1 [1]	3 [3]
1231200		12	1394718	1396231	1513	-	-	2
1231300		12	1397303	1398631	1328	-	1	2 [1]
1231400		12	1399326	1401845	2519	-	5 [4]	8 [7]
1231600		12	1406272	1412350	6078	-	4 [2]	13 [6]
1231700		12	1414465	1419855	5390	-	7 [2]	10 [4]
1231900		12	1423453	1424147	694	-	-	1
1232000	SRP54	12	1426296	1427798	1502	-	-	4
1232100		12	1429671	1432326	2655	-	6	7
1232300		12	1437400	1437888	488	-	1	1
1232400	RRP6	12	1439159	1442533	3374	-	12 [6]	21 [10]
1232500		12	1443877	1445343	1466	-	1	6 [1]
1232600	ApiAP2	12	1447105	1449751	2646	-	14 [11]	23 [11]
1232900		12	1470495	1471363	868	-	-	1
1233300		12	1484724	1485750	1026	-	-	1
1233400		12	1486382	1487314	932	-	-	2 [1]
1233700		12	1501353	1504028	2675	-	1	4 [1]
1233800		12	1507421	1511872	4451	-	-	8 [4]
1233900		12	1513089	1518893	5804	-	1	5
1234100	UTP12	12	1520922	1524800	3878	-	1	4 [1]
1234200	MDR2	12	1526862	1531049	4187	-	-	4 [2]
1234300	ApiAP2	12	1533462	1535516	2054	1	2 [1]	2 [1]
1234600		12	1543308	1544224	916	-	-	1
1234800		12	1547188	1548737	1549	3	-	-
1235000		12	1550965	1552789	1824	-	-	2 [1]
1235200		12	1555577	1557172	1595	-	1	-
1235300		12	1560585	1561831	1246	8 [4]	1 [1]	1 [1]

1235400	HDP	12	1562588	1563592	1004	6 [1]	-	3 [1]
1235500		12	1564959	1567053	2094	6 [1]	-	-
1235900		12	1586110	1589520	3410	1 [1]	7 [4]	13 [6]
1236000		12	1592332	1594161	1829	1	1	-
1236100		12	1595479	1597905	2426	2 [1]	-	3 [1]
1236400		12	1608657	1612465	3808	1 [1]	3 [2]	10 [7]
1236600		12	1617308	1620334	3026	5 [1]	1	2 [1]
1236900		12	1632359	1635019	2660	1	-	-
1237000		12	1636094	1638077	1983	1	-	-
1237200		12	1643334	1646504	3170	-	-	2 [1]
1237300		12	1647460	1648656	1196	9 [1]	2	-
1237400		12	1649334	1649900	566	-	1 [1]	1 [1]
1237500		12	1653137	1654622	1485	2	-	2
1237600		12	1655956	1657167	1211	2 [1]	-	1
1237800		12	1660764	1665480	4716	1 [1]	1	5 [1]
1237900		12	1666843	1668636	1793	-	1 [1]	1
1238000	LPAAT	12	1669839	1671161	1322	1 [1]	5 [4]	4 [3]
1238100		12	1672859	1673984	1125	-	2	5
1238200		12	1674668	1697299	22631	4 [2]	4 [3]	7 [3]
1238400		12	1699432	1702314	2882	3 [1]	-	1 [1]
1238500		12	1703740	1704942	1202	-	-	1
1238600	DPCK	12	1706985	1708165	1180	1	-	-
1238700		12	1709215	1714734	5519	20 [9]	1	6 [4]
1238800	PARN	12	1716020	1718593	2573	7 [3]	-	2 [1]
1238900		12	1720071	1722879	2808	-	6	3
1239100		12	1724706	1726720	2014	2 [2]	3 [1]	6 [3]
1239200		12	1728752	1730503	1751	-	-	1 [1]
1239300	SRPK2	12	1732356	1736252	3896	1 [1]	-	8 [4]
1239400	SEC7	12	1736966	1745851	8885	2 [1]	4	17 [5]
1239500		12	1747727	1748935	1208	-	1	5 [3]
1239600		12	1749291	1756513	7222	17 [10]	17 [10]	37 [24]
1239700		12	1758094	1764469	6375	44 [22]	13 [8]	4 [3]
1239800		12	1767719	1771166	3447	30 [8]	-	4 [1]
1239900		12	1772121	1780250	8129	37 [22]	3 [2]	5 [4]
1240000		12	1783788	1785383	1595	5	-	1
1240100		12	1786459	1789356	2897	11 [7]	1	2
1240300		12	1792626	1793426	800	4	2	1
1240500		12	1794923	1796051	1128	3	-	1
1240600		12	1796953	1798422	1469	2	-	-
1240700		12	1802269	1804056	1787	9 [5]	1 [1]	1
1240800		12	1805020	1807049	2029	6 [2]	1 [1]	-
1240900	FACT-S	12	1807929	1809446	1517	3	-	1
1241000		12	1811883	1819956	8073	5 [5]	2 [2]	1 [1]
1241100		12	1822900	1824166	1266	2	-	3
1241200		12	1829827	1830540	713	3	-	-
1241300	NCS2	12	1832492	1834243	1751	8 [3]	2 [1]	2
1241400		12	1834506	1836225	1719	5 [2]	-	4 [1]
1241600		12	1843031	1845220	2189	2 [2]	-	3 [1]
1241700		12	1845618	1849676	4058	-	-	5
1241800		12	1851085	1852611	1526	-	1	1
1241900		12	1855949	1859754	3805	2 [1]	-	1
1242000		12	1860386	1868363	7977	2	1	8 [1]
1242200		12	1871558	1874746	3188	-	-	6 [2]
1242300		12	1877063	1883556	6493	2 [1]	5 [2]	8 [6]
1242400	TIM	12	1884353	1885279	926	-	1	2
1242500		12	1888930	1889896	966	-	-	1
1242600		12	1893526	1895569	2043	-	6 [1]	7 [1]
1242800		12	1898599	1902180	3581	10 [7]	10 [4]	4 [1]
1242900		12	1908846	1909919	1073	-	2	3
1243000		12	1911232	1916541	5309	-	-	2 [1]
1243500		12	1937783	1941964	4181	2	-	7
1243700		12	1957562	1961777	4215	-	2	-
1243900		12	1965507	1968323	2816	-	-	1
1244300		12	1984967	1986787	1820	-	1	4
1244400		12	1987871	1988465	594	-	3 [1]	4 [1]
1244600		12	2004410	2007044	2634	1	-	3 [1]
1244700		12	2007826	2018667	10841	4 [1]	1	8 [2]
1244800	SF3A1	12	2019079	2020959	1880	-	-	2 [1]

1244900		12	2023427	2025445	2018	3 [1]	1 [1]	-
1245000		12	2025971	2028543	2572	6	1	1
1245100		12	2030353	2031603	1250	-	-	1
1245400		12	2033159	2037358	4199	7 [3]	1	1 [1]
1245500		12	2038251	2041037	2786	1	-	1 [1]
1245700	CRMP4	12	2043472	2060038	16566	28 [16]	13 [10]	11 [5]
1245800	CCp1	12	2063214	2068139	4925	-	-	1
1245900		12	2069582	2071846	2264	10 [5]	-	2 [1]
1246000		12	2074239	2075735	1496	3	1 [1]	-
1246200		12	2083200	2084966	1766	10 [5]	-	2 [1]
1246300		12	2087277	2087730	453	4 [2]	1 [1]	3 [1]
1246400	ETRAMP	12	2091475	2092002	527	3 [1]	-	1 [1]
1246500		12	2094098	2096799	2701	49 [41]	9 [9]	4 [3]
1246600		12	2099000	2099912	912	3 [2]	-	2 [1]
1246800		12	2107943	2110430	2487	8 [6]	1 [1]	2 [2]
1246900		12	2113188	2114536	1348	5 [3]	2 [2]	-
1247600		12	2154922	2156031	1109	5 [2]	-	2 [1]
1247700		12	2158065	2158957	892	8 [1]	-	3
1247800		12	2160989	2162051	1062	5 [4]	-	2 [2]
1247900		12	2166892	2167525	633	2	-	1
1248000		12	2170881	2172879	1998	11 [4]	3 [3]	4 [2]
1248100		12	2175930	2178034	2104	5 [3]	2 [2]	-
1248200		12	2179191	2182262	3071	10 [4]	1 [1]	1 [1]
1248300		12	2183259	2190227	6968	26 [12]	2 [1]	1
1248400		12	2192343	2195810	3467	10 [1]	2	3 [1]
1248500		12	2197391	2198390	999	1 [1]	-	-
1248600		12	2200435	2204919	4484	11 [4]	5 [2]	5 [2]
1248700		12	2206653	2208458	1805	4 [3]	-	2 [1]
1248800	UTP15	12	2209099	2211837	2738	10 [1]	1	-
1248900	ABCB6	12	2213480	2216428	2948	6 [2]	2	-
1249000		12	2217180	2222783	5603	15 [8]	4 [1]	3 [2]
1249100		12	2223488	2224714	1226	5 [4]	-	1 [1]
1249200		12	2225331	2226732	1401	2	-	-
1249300	IMC1f	12	2228198	2232481	4283	23 [13]	1	1 [1]
1249400		12	2235453	2236958	1505	2	-	-
1249500		12	2238894	2240507	1613	5	-	1
1249600		12	2244445	2245875	1430	6 [3]	1	-
1249800		12	2252401	2253629	1228	14	-	-
1250100		12	2276993	2277808	815	1 [1]	-	-
1250200		12	2278098	2279300	1202	3 [2]	-	-
1250300		12	2280569	2281517	948	1	-	-
1250400		12	2284730	2288191	3461	34 [19]	8 [5]	4 [3]
1250500		12	2288939	2290744	1805	4 [4]	4 [2]	2 [1]
1250700	SNRPE	12	2293564	2294273	709	-	-	1
1250800		12	2295464	2297438	1974	1 [1]	2 [2]	3 [2]
1250900		12	2298062	2299874	1812	-	-	2
1251000		12	2300575	2301123	548	1 [1]	-	3 [2]
1251100		12	2301942	2303195	1253	1	-	-
1251200		12	2304410	2305433	1023	1 [1]	-	-
1251400		12	2308159	2316308	8149	9 [3]	2	8 [4]
1251500		12	2316845	2318226	1381	-	1	1
1251600	TKL3	12	2319174	2324075	4901	11 [8]	2 [2]	2 [1]
1251700		12	2327800	2330208	2408	-	-	3 [2]
1252100		12	2337794	2343624	5830	-	4 [2]	4 [3]
1252200	WDR16	12	2344979	2348252	3273	-	1 [1]	3 [2]
1252300		12	2348642	2350201	1559	-	-	1 [1]
1252400		12	2351826	2353290	1464	-	1	2
1252500		12	2356425	2367065	10640	-	-	6 [3]
1252600		12	2369031	2373602	4571	-	-	2 [1]
1253100	LRR4.1	12	2391365	2392998	1633	-	-	1
1253300		12	2400701	2403608	2907	-	-	9 [2]
1253600		12	2412825	2414683	1858	-	-	2
1253700	NT1	12	2416888	2418138	1250	-	-	1
1253900	WDR92	12	2422659	2424876	2217	-	-	4 [2]
1254500		12	2467368	2468030	662	3 [3]	-	-
1254600		12	2468444	2470150	1706	1	-	1
1254700		12	2471193	2487281	16088	4 [3]	-	8 [4]
1254800	ALBA2	12	2489816	2490808	992	1	-	1

1255200	SEC61	12	2499615	2501420	1805	-	-	2
1255500		12	2510631	2514024	3393	1	-	1
1255600	IDH	12	2516663	2518072	1409	1	1	2
1255700		12	2518909	2520238	1329	1	-	2 [1]
1255800		12	2523102	2523890	788	-	-	1
1256200	TRX2	12	2538932	2540087	1155	5 [2]	-	3 [1]
1256300		12	2540235	2543229	2994	2	-	7
1256400		12	2544807	2549450	4643	8 [3]	-	4 [3]
1256500		12	2553117	2554752	1635	1 [1]	-	1 [1]
1256600		12	2556095	2559565	3470	2 [1]	1 [1]	5 [3]
1256800	USB1	12	2562024	2563304	1280	-	-	4
1256900		12	2565011	2566366	1355	-	-	1 [1]
1257400		12	2593722	2596211	2489	-	-	1 [1]
1257900		12	2632962	2634533	1571	-	-	1
1258000	RAD5	12	2635616	2640402	4786	-	1	2 [1]
1258100		12	2643826	2646567	2741	1 [1]	3 [1]	-
1258200		12	2648920	2650383	1463	-	-	2
1258300		12	2655296	2656264	968	1	-	2
1258500	ApiAP2	12	2663436	2674892	11456	3 [1]	2	7 [4]
1258600	PEPCK	12	2675924	2677723	1799	2	-	1
1258800	MyoA	12	2681043	2683871	2828	8 [1]	-	2 [1]
1259000	CK2beta2	12	2690956	2692344	1388	1	-	1
1259200		12	2695131	2697719	2588	1	1	-
1259300		12	2698334	2701060	2726	3	1	-
1259400		12	2705164	2706138	974	6 [1]	-	2 [1]
1259500		12	2709074	2710384	1310	1	-	1
1259600	IMC1k	12	2712334	2713680	1346	1	-	-
1259900	ISC1	12	2719669	2721189	1520	-	2	1
1260500	PiT	12	2732551	2734639	2088	7 [2]	3 [1]	1
1260900		12	2748520	2752017	3497	-	-	1
1261300	RRP42	12	2760549	2761394	845	-	1	-
1261500	ABCB5	12	2768313	2770787	2474	1 [1]	2	1
1261700		12	2773840	2778618	4778	4 [1]	3 [2]	1 [1]
1262400		12	2801201	2803840	2639	1	4 [2]	5 [4]
1262600		12	2809987	2811963	1976	-	-	1
1262800		12	2817043	2819232	2189	-	1	3 [1]
1263000		12	2823689	2825292	1603	-	-	1
1263300		12	2833964	2838676	4712	-	2	2
1263500	CDPK5	12	2844000	2845691	1691	-	-	4 [1]
1263600		12	2846246	2848618	2372	1 [1]	-	1 [1]
1263900		12	2861618	2863929	2311	-	-	2 [2]
1264000		12	2864891	2867683	2792	1	1	3 [1]
1264100	DXS	12	2869187	2872555	3368	-	2 [1]	7 [1]
1264200	PK6	12	2873053	2875117	2064	-	-	1
1264300		12	2876059	2879142	3083	-	-	1
1264400		12	2880226	2882507	2281	-	-	1
1264800		12	2891501	2893785	2284	-	-	3
1265100		12	2895975	2897788	1813	-	-	1
1265400	TRAP	12	2906844	2908583	1739	11 [8]	6 [5]	7 [4]
1265800		12	2919721	2921528	1807	-	1 [1]	1 [1]
1265900		12	2924552	2925379	827	-	1 [1]	1 [1]
1266200		12	2931549	2932019	470	1 [1]	1 [1]	-
1266500		12	2940575	2943391	2816	-	1	5
1266700		12	2947600	2951677	4077	-	1	5 [3]
1266800		12	2953794	2954723	929	-	-	1
1266900	CenH3	12	2956666	2957184	518	-	1	1
1267000	UTP4	12	2958622	2962134	3512	-	5 [3]	7 [2]
1267100		12	2962677	2963855	1178	-	1	1
1300400		13	25467	26437	970	7 [3]	-	1 [1]
1300500		13	30496	32796	2300	12 [11]	-	-
1300600		13	35167	36212	1045	8 [5]	-	1
1300900		13	49289	50554	1265	6 [1]	-	1
1301000		13	54290	55452	1162	2 [1]	-	1 [1]
1301100		13	58531	59682	1151	6 [6]	1 [1]	2 [2]
1301200	LSAP1	13	64101	64415	314	1 [1]	-	1 [1]
1301300		13	66478	67889	1411	3 [1]	-	3 [2]
1301400		13	70046	71533	1487	2	-	-
1301500	NEK3	13	73128	74018	890	1 [1]	-	-

1301600		13	75986	77095	1109	5 [4]	-	3 [2]
1301800		13	80855	82042	1187	1	-	-
1301900		13	83002	86832	3830	18 [2]	-	2 [1]
1302000		13	87457	89689	2232	7 [1]	-	-
1302200		13	93852	116534	22682	12 [8]	1 [1]	14 [6]
1302300	CYP26	13	116650	118471	1821	2	-	2
1302400		13	119498	122461	2963	-	-	3 [2]
1302700		13	137374	138045	671	2 [1]	-	3 [1]
1302900	ORC1	13	145816	149265	3449	1	-	4 [3]
1303200		13	152600	155035	2435	-	-	2
1303300	SUA5	13	157367	159004	1637	-	1	1
1303800		13	174058	176721	2663	-	1 [1]	-
1304000	EIF5A	13	180328	180813	485	1	1	2
1304100		13	183773	186635	2862	-	-	1
1305000		13	211910	212884	974	-	1	1
1305200		13	225139	230313	5174	-	-	5 [1]
1305300		13	232913	235050	2137	-	2	2
1305500	HMGB3	13	241489	249544	8055	-	1 [1]	3 [1]
1305600		13	250652	254155	3503	-	1 [1]	6 [5]
1306800		13	318536	320629	2093	-	-	2 [1]
1306900		13	321472	324241	2769	-	2 [2]	4 [4]
1307000		13	325243	333125	7882	-	-	3 [2]
1308000		13	361235	374342	13107	-	-	1 [1]
1308100	CRMP3	13	375444	386199	10755	-	1	2
1308200		13	387477	388134	657	1 [1]	-	1 [1]
1308300		13	390483	395111	4628	-	2 [1]	3 [3]
1308400		13	395626	399969	4343	-	-	1 [1]
1308600		13	402139	403546	1407	-	-	4
1308700		13	404458	407295	2837	-	-	6 [4]
1308800		13	415402	419646	4244	-	-	1
1309000		13	423947	426844	2897	-	3 [2]	7 [4]
1309200	KROX1	13	430324	434811	4487	-	6 [6]	10 [9]
1309300	CIA1	13	439395	441626	2231	-	1 [1]	1 [1]
1309400		13	443587	446988	3401	-	-	4 [1]
1309600		13	450233	451925	1692	-	-	1
1310200		13	473158	476349	3191	-	1	1
1310500		13	484995	488136	3141	-	-	2
1311100		13	506411	511667	5256	-	1 [1]	2 [1]
1311700	LSD1	13	533530	540307	6777	-	-	1 [1]
1312000	ATP4	13	551027	554908	3881	-	1	5 [2]
1312800		13	610171	614693	4522	-	4 [2]	5 [3]
1313200	PLP4	13	624480	626537	2057	-	-	1 [1]
1313400		13	632004	633271	1267	-	1 [1]	-
1313800	SRCAP	13	647743	653463	5720	-	1	1
1314600		13	683331	685039	1708	-	1 [1]	1 [1]
1314700		13	686843	690130	3287	-	3	6 [1]
1314800		13	690883	693114	2231	-	1	1
1315100		13	707663	710299	2636	-	1 [1]	3 [2]
1315700		13	728576	729354	778	-	1 [1]	1 [1]
1315800		13	731268	731959	691	-	1	1
1316000		13	734125	736027	1902	-	1	-
1316200		13	738172	742788	4616	-	3 [3]	3 [3]
1316400		13	745505	747876	2371	-	1 [1]	-
1316600	SEC23	13	752232	754695	2463	-	1	1
1316700		13	755308	757527	2219	-	9 [5]	8 [5]
1316800		13	759579	760580	1001	-	1 [1]	-
1317000	VPS15	13	766482	770393	3911	-	1 [1]	2 [2]
1317800		13	797408	799489	2081	-	4 [3]	5 [3]
1318300		13	813895	816159	2264	-	1 [1]	1 [1]
1320300		13	888924	913865	24941	-	1 [1]	1 [1]
1320700	UBE4B	13	928726	932421	3695	-	5 [1]	6 [2]
1321100	DBP10	13	944415	947900	3485	-	4 [1]	6
1321200	IF2c	13	949231	952860	3629	1	1	-
1321300		13	953747	957364	3617	-	2 [1]	2 [1]
1321800		13	972062	974332	2270	-	3 [3]	3 [3]
1321900		13	976969	982779	5810	-	10 [5]	10 [5]
1322000		13	983921	985592	1671	-	-	1
1322600		13	1003684	1005546	1862	-	1	2

1322700	FT1	13	1007395	1009360	1965	-	1	1
1322900	GAMA	13	1015374	1017542	2168	-	-	1 [1]
1323100		13	1024059	1029124	5065	-	2 [2]	2 [2]
1323700		13	1045889	1053508	7619	-	1	2 [1]
1324100	SIAP2	13	1080087	1081322	1235	8 [7]	2 [2]	3 [3]
1324200		13	1087416	1088334	918	1 [1]	-	1 [1]
1324300		13	1090531	1092064	1533	11 [6]	-	-
1324400		13	1097610	1098996	1386	3 [3]	3 [3]	-
1324600		13	1105011	1107023	2012	3 [2]	2 [2]	6 [3]
1325500		13	1198688	1200354	1666	7 [5]	3 [2]	-
1325600		13	1204426	1205612	1186	9 [8]	-	3 [3]
1325800		13	1215366	1218431	3065	18 [12]	2 [2]	1 [1]
1325900		13	1222513	1223133	620	5 [2]	-	1 [1]
1326000		13	1224984	1226733	1749	12 [5]	-	1 [1]
1326100		13	1230291	1231411	1120	8 [8]	2 [2]	-
1326200		13	1235122	1236191	1069	9 [5]	8 [6]	-
1326400		13	1245114	1245885	771	4 [2]	-	1 [1]
1326700		13	1274323	1276119	1796	10 [1]	-	2 [1]
1326800		13	1277858	1279246	1388	4 [1]	1	-
1326900	ISC3	13	1280014	1283603	3589	7 [1]	-	6 [3]
1327400		13	1298572	1301168	2596	-	2	1
1327500		13	1303536	1306310	2774	1 [1]	10 [6]	12 [4]
1327600		13	1307721	1308770	1049	1	1	-
1327700		13	1311778	1312766	988	1	-	1
1327800		13	1314166	1314684	518	1 [1]	1	-
1328000		13	1317331	1319324	1993	2	2	1
1328200		13	1323956	1325143	1187	1	3 [2]	-
1328300	ATG5	13	1325664	1327499	1835	-	1	2
1328400		13	1328662	1335261	6599	5 [1]	6 [3]	6 [4]
1328600	PTPA	13	1340168	1341808	1640	-	1	1
1328800	WRN	13	1345756	1349514	3758	4	-	1
1328900		13	1350661	1354917	4256	2 [1]	1	1 [1]
1329000		13	1356852	1357919	1067	-	-	1
1329300		13	1362993	1369736	6743	3 [2]	-	6 [1]
1329400		13	1371049	1374464	3415	11	-	4
1329700		13	1383458	1384138	680	1	-	2 [1]
1329800		13	1385055	1388477	3422	14 [7]	2 [2]	6 [3]
1329900		13	1388648	1389885	1237	2	-	1
1330000		13	1391602	1392480	878	2 [2]	-	1 [1]
1330100		13	1393024	1395178	2154	4	-	2
1330400		13	1430623	1431901	1278	1 [1]	-	-
1330500		13	1432979	1435606	2627	1	-	1
1331100		13	1450552	1452000	1448	-	1	1
1331400		13	1470183	1475380	5197	-	1 [1]	2 [1]
1331600		13	1479780	1484378	4598	6 [5]	3 [3]	7 [3]
1331700		13	1486303	1489707	3404	1 [1]	1 [1]	5 [3]
1331900		13	1491875	1497779	5904	1 [1]	-	12 [2]
1332000	PEPC	13	1501952	1505440	3488	4 [1]	2 [1]	-
1332200	ABCG2	13	1511581	1513635	2054	1 [1]	-	1 [1]
1332300		13	1514401	1515457	1056	1	-	-
1332400		13	1515550	1517406	1856	1	-	-
1332600		13	1522964	1523488	524	1	1	1
1332800		13	1527675	1529029	1354	1 [1]	-	-
1332900		13	1529407	1531815	2408	-	2 [1]	1
1333000		13	1532721	1534469	1748	3 [1]	-	1
1333100		13	1536387	1537093	706	-	3 [2]	2 [1]
1333200		13	1537936	1540908	2972	1	4 [1]	4 [1]
1333400		13	1545108	1546553	1445	-	-	1
1334200	VPS3	13	1559565	1563988	4423	1	1	1
1334300		13	1567127	1571380	4253	21 [11]	16 [10]	7 [5]
1334400		13	1572295	1574359	2064	-	-	4
1334500		13	1577861	1581598	3737	4 [3]	1 [1]	10 [8]
1334600		13	1581949	1586742	4793	-	-	3 [1]
1334700	PP7	13	1587162	1592313	5151	5 [1]	-	5
1334800	CYP52	13	1593126	1595482	2356	-	-	3
1334900		13	1597204	1599477	2273	-	3 [1]	7 [3]
1335000	NOG2	13	1603560	1605251	1691	-	1 [1]	3 [1]
1335100		13	1605968	1607734	1766	2 [2]	-	3 [3]

1335300		13	1612479	1615571	3092	2 [2]	4 [3]	9 [5]
1335400		13	1616865	1618409	1544	-	-	1 [1]
1335500	HRD1	13	1619880	1622260	2380	-	1	1
1335600		13	1624004	1628617	4613	5 [3]	2	7 [3]
1335900		13	1635302	1636637	1335	2 [1]	1	1 [1]
1336000		13	1637224	1637791	567	-	-	1
1336400	TIM8	13	1644260	1644937	677	-	-	2
1336600		13	1648116	1651813	3697	-	1	4 [2]
1336900		13	1654903	1657533	2630	1	-	3 [2]
1337300	P113	13	1665639	1668619	2980	1 [1]	1 [1]	3 [2]
1337600	GlyRS	13	1677007	1679499	2492	-	-	2
1337700		13	1680874	1681677	803	-	1 [1]	-
1337800		13	1682661	1686134	3473	-	-	1
1337900		13	1688801	1693378	4577	6 [6]	2 [1]	12 [11]
1338000	SF3B4	13	1695204	1696812	1608	2	-	1
1338100		13	1698150	1699988	1838	1	3 [1]	-
1338400		13	1707525	1711117	3592	16 [4]	-	4 [3]
1339000		13	1735931	1739221	3290	4 [2]	-	2 [1]
1339100	DBP4	13	1740567	1743353	2786	1	-	1
1339700		13	1750513	1750923	410	1 [1]	-	1 [1]
1339800		13	1751377	1752342	965	6	4	-
1339900	LISP1	13	1754647	1765017	10370	11 [3]	-	4 [1]
1340000	UFD1	13	1766470	1768709	2239	-	-	1
1340400		13	1782338	1795564	13226	7 [3]	-	13 [5]
1340600	EPAC	13	1800825	1812024	11199	1	-	1 [1]
1340900		13	1832868	1846298	13430	3 [2]	1 [1]	7 [5]
1341000		13	1849640	1850593	953	-	1 [1]	1 [1]
1341200		13	1855989	1856943	954	1	-	1
1341300		13	1857424	1859430	2006	1 [1]	-	3 [2]
1341400		13	1859932	1861063	1131	1 [1]	-	2 [1]
1341500		13	1863658	1872393	8735	1 [1]	-	9 [3]
1341600		13	1873628	1875040	1412	-	-	2
1341700		13	1877242	1882112	4870	6	2	2
1341800		13	1882351	1883651	1300	-	-	1 [1]
1341900	MCA3	13	1885692	1891586	5894	16 [9]	7 [1]	6 [4]
1342000	SEY1	13	1894145	1896796	2651	4 [2]	2 [1]	3
1342100		13	1897672	1898916	1244	2	-	3
1342200		13	1900636	1901461	825	4 [1]	1	1
1342400		13	1908266	1909981	1715	6	4	-
1342500		13	1912125	1915280	3155	19 [9]	-	4 [1]
1342600		13	1916504	1920334	3830	17 [10]	4 [1]	3 [1]
1342800		13	1930637	1931641	1004	2	-	1
1343200		13	1943886	1946981	3095	1 [1]	-	1 [1]
1343300	SMB1	13	1948350	1949322	972	-	-	1
1343500		13	1957545	1959038	1493	-	-	1 [1]
1343600		13	1961893	1969479	7586	-	-	10 [6]
1343900		13	1984789	1985775	986	-	-	1 [1]
1344000		13	1987415	1989751	2336	-	-	1 [1]
1344600		13	2030623	2036358	5735	-	-	2 [2]
1344700		13	2036608	2038676	2068	-	-	1 [1]
1344900		13	2042386	2044167	1781	-	-	1
1345100		13	2047934	2050292	2358	-	1 [1]	3 [2]
1345200		13	2051006	2052960	1954	-	-	3
1345300		13	2055491	2056837	1346	-	1	2
1345400		13	2058680	2060817	2137	-	-	1
1345500	NMT	13	2063386	2065247	1861	-	1	1
1345700	DHS	13	2070307	2071650	1343	-	1	-
1345800		13	2073018	2074260	1242	-	-	1
1345900		13	2077827	2086949	9122	-	2 [1]	12 [5]
1346000	NTF2	13	2089492	2090238	746	-	-	1
1346100		13	2091818	2094626	2808	-	-	1
1346500		13	2105509	2106876	1367	11 [6]	-	-
1346600		13	2107152	2108804	1652	11 [7]	-	-
1346700		13	2109300	2111027	1727	11 [9]	2 [2]	-
1346800		13	2111966	2114915	2949	-	-	4 [2]
1346900	PREX	13	2116681	2122266	5585	2	-	-
1347000		13	2122849	2125043	2194	1 [1]	-	3 [2]
1347100	ROM8	13	2129103	2131945	2842	2 [1]	-	3

1347200		13	2134762	2137057	2295	-	-	1
1347300		13	2137294	2144983	7689	4 [2]	-	7 [2]
1347400		13	2145810	2148280	2470	-	-	1 [1]
1347500		13	2150365	2151864	1499	1	-	1
1347600		13	2155019	2156180	1161	1	-	1
1347700		13	2157565	2158962	1397	-	-	1
1347800		13	2162181	2162804	623	-	-	2 [1]
1347900	RAP1	13	2164214	2166124	1910	9 [7]	9 [6]	14 [9]
1348000		13	2170109	2182363	12254	8 [4]	2 [1]	26 [13]
1348100		13	2183877	2186429	2552	2	-	3
1348200		13	2189293	2191398	2105	-	-	1
1348300	EMC2	13	2193872	2194753	881	1 [1]	-	2 [2]
1348400	CDS	13	2196730	2198652	1922	15 [8]	9 [7]	3
1348600		13	2209469	2212011	2542	13 [4]	-	1 [1]
1348700		13	2212734	2217071	4337	21 [15]	-	4 [2]
1348800		13	2219721	2221424	1703	6 [5]	-	4 [3]
1348900		13	2223940	2227986	4046	17 [11]	2 [1]	6 [4]
1349000		13	2232800	2234311	1511	2	-	2
1349100	DDI1	13	2235646	2236827	1181	1 [1]	1 [1]	2 [2]
1349300		13	2240726	2244136	3410	1 [1]	-	7 [7]
1349400		13	2245268	2247019	1751	2 [1]	-	3 [1]
1349700		13	2254315	2273955	19640	9 [2]	1	22 [9]
1349900		13	2278862	2279395	533	-	-	1 [1]
1350000		13	2281575	2284895	3320	2 [2]	1 [1]	5 [1]
1350100		13	2285970	2287161	1191	2 [1]	-	4 [3]
1350200	AP2-G2	13	2296199	2300851	4652	25 [15]	-	3 [2]
1350300		13	2302686	2304038	1352	3 [2]	1 [1]	-
1350500		13	2312140	2318064	5924	6 [6]	-	10 [7]
1350600		13	2318085	2319356	1271	-	1	2
1351100	CCp3	13	2333521	2337462	3941	1 [1]	-	2 [1]
1351200		13	2338737	2340436	1699	-	-	2
1351300	GAPM3	13	2344723	2346130	1407	4	-	1
1351500		13	2349186	2352917	3731	5 [3]	1	3 [1]
1351600	WDR65	13	2354779	2359448	4669	1 [1]	-	-
1351700		13	2359528	2361429	1901	2	1 [1]	-
1351800	GDPD	13	2362358	2363911	1553	1 [1]	1 [1]	4 [3]
1352100		13	2380529	2381251	722	-	-	1
1352200		13	2384515	2387726	3211	-	-	2
1352300	BOP1	13	2389071	2392649	3578	3	-	1
1352400		13	2395215	2397320	2105	1 [1]	-	2 [1]
1352600	CBWD1	13	2401554	2403230	1676	3 [2]	1	2 [2]
1353000		13	2413914	2417679	3765	-	1 [1]	-
1353100		13	2418471	2421572	3101	2	3	1 [1]
1353200		13	2423615	2424586	971	1 [1]	-	1 [1]
1353300		13	2427504	2430062	2558	19 [13]	4 [3]	4 [3]
1353400		13	2432223	2433311	1088	11 [8]	7 [5]	1 [1]
1353600	RRP5	13	2441128	2442507	1379	2	-	-
1353700		13	2443940	2444479	539	-	1	-
1353900		13	2448049	2448652	603	2	-	-
1354000		13	2451376	2451936	560	2	-	-
1354100		13	2453025	2453790	765	3 [1]	-	-
1354300	MISFIT	13	2457042	2461978	4936	15 [8]	1	7 [4]
1354400	TRAM	13	2464067	2465459	1392	-	-	1
1354500	Sel1	13	2466340	2467053	713	1 [1]	-	-
1354600		13	2467641	2469816	2175	10 [2]	-	1
1354700		13	2470037	2474273	4236	9	1	6
1354800		13	2474870	2478670	3800	10 [7]	-	4 [4]
1354900		13	2479669	2480745	1076	6 [4]	-	1
1355200		13	2492551	2495019	2468	4 [3]	-	5 [4]
1355400	SR140	13	2503405	2506868	3463	4	-	14 [2]
1355500		13	2508678	2510260	1582	-	-	1
1355700		13	2511687	2513351	1664	1	-	4 [3]
1356000		13	2519765	2522131	2366	1	-	3 [2]
1356100		13	2523508	2527367	3859	2	-	6 [1]
1356400		13	2540275	2541909	1634	9 [1]	-	1 [1]
1356500		13	2543211	2544843	1632	19 [15]	5 [4]	1 [1]
1356600		13	2547560	2550011	2451	2 [2]	2 [1]	-
1356700		13	2551274	2553733	2459	2 [1]	-	1 [1]

1356900		13	2559448	2563258	3810	18 [10]	10 [8]	5 [3]
1400800		14	36140	40197	4057	14 [9]	-	1 [1]
1401000		14	47416	48675	1259	14 [11]	2 [2]	-
1401100		14	50671	51495	824	1 [1]	-	-
1401200		14	56384	58762	2378	7 [4]	1 [1]	-
1401300		14	60904	66415	5511	28 [7]	2 [1]	4
1401400		14	70091	70853	762	7 [5]	3 [3]	-
1401500		14	74370	75650	1280	4 [4]	-	4 [3]
1401600		14	82065	83158	1093	10 [9]	1 [1]	-
1401700		14	86521	87926	1405	26 [18]	7 [5]	4 [3]
1401800		14	91998	93107	1109	12 [7]	-	1
1401900		14	94406	95082	676	1 [1]	1 [1]	-
1402000		14	96627	97420	793	2	-	-
1402100		14	99980	101136	1156	11 [7]	3 [3]	1
1402200		14	103978	104953	975	2	-	-
1402300	GAP	14	108371	109105	734	7 [3]	1	-
1402400	ETRAMP	14	112475	113116	641	2 [1]	-	-
1402500		14	115433	116151	718	-	-	1 [1]
1402600		14	118641	120534	1893	7	-	-
1402700		14	122317	130750	8433	7 [2]	4 [2]	4 [2]
1402900		14	136092	141236	5144	-	-	1 [1]
1403000		14	142075	142858	783	1	-	1
1403100		14	146209	148893	2684	-	-	1
1403200		14	150042	150868	826	2	-	-
1403300		14	151183	152081	898	6	-	-
1403400		14	153119	154488	1369	1	1	-
1403600		14	158161	161091	2930	23 [9]	-	2 [1]
1404000		14	180806	206764	25958	-	3 [2]	10 [7]
1404200		14	212094	215433	3339	-	-	1
1405300		14	258636	259892	1256	-	1	-
1405500		14	262809	273275	10466	-	1 [1]	2 [1]
1405600		14	275521	277670	2149	-	-	1 [1]
1405900		14	284723	285313	590	-	1	1
1406700		14	311846	323980	12134	-	-	1
1408000		14	371487	375134	3647	1	1	-
1408100		14	376040	377756	1716	-	-	1 [1]
1408200		14	377891	380245	2354	-	-	3 [1]
1408600		14	391990	398761	6771	-	-	1 [1]
1408800		14	403848	419160	15312	-	9 [7]	12 [8]
1409000		14	424851	428936	4085	-	2 [1]	2 [1]
1409100		14	429752	431002	1250	-	-	1 [1]
1409200	INT	14	431593	434819	3226	-	-	1
1409300	DCP2	14	438648	442883	4235	-	-	2 [1]
1409400		14	447360	448442	1082	-	1	2
1409600	PPM6	14	458117	460664	2547	-	2 [1]	3 [1]
1409700	PRPF3	14	461337	463547	2210	-	-	3 [1]
1409800		14	466702	470079	3377	-	1 [1]	3 [3]
1410100	VPS18	14	474648	479306	4658	-	1	1
1411100		14	503777	504247	470	-	-	1 [1]
1411500		14	515412	516899	1487	-	-	1
1412000		14	531672	533285	1613	-	2 [2]	2 [2]
1413000		14	596992	597780	788	-	-	1 [1]
1413100	IF2a	14	598614	601835	3221	-	-	2
1413500	EIF4G	14	623905	627675	3770	11 [7]	-	2 [1]
1413700		14	633009	641425	8416	6 [1]	-	-
1413800		14	642387	644396	2009	1 [1]	-	1 [1]
1414200		14	655920	662000	6080	-	1 [1]	5 [3]
1414400		14	665469	675288	9819	1 [1]	-	3 [3]
1414600		14	679537	683103	3566	-	-	2 [1]
1414700		14	684901	687090	2189	-	2 [1]	2 [1]
1414800	TERT	14	688636	695490	6854	-	5 [3]	5 [3]
1415300		14	703864	706012	2148	-	1	-
1416100		14	732589	735147	2558	-	-	1
1416200		14	738783	739870	1087	2 [1]	-	-
1416300	PIS	14	741318	743421	2103	2	-	2
1416600		14	750535	754730	4195	8 [2]	-	2 [1]
1416800	IPK2	14	761921	765868	3947	-	2 [2]	3 [3]
1417000		14	768863	769653	790	2	-	2

1417200	PRP40	14	774541	777129	2588	-	5 [2]	7 [3]
1417300	CCT	14	780403	783720	3317	1	-	2
1417400		14	785110	787384	2274	4	-	9 [1]
1417500	SEC20	14	787709	789691	1982	1	-	6 [1]
1417600		14	790965	793439	2474	-	-	2 [1]
1417700	USP39	14	797070	799118	2048	-	-	3 [2]
1417800	MCM4	14	800699	803614	2915	2 [1]	-	-
1417900	ApiAP2	14	813121	819372	6251	48 [35]	10 [8]	2 [2]
1418000		14	820145	821777	1632	7 [2]	4 [1]	-
1418100		14	823546	824160	614	1 [1]	-	-
1418200		14	825251	825520	269	-	-	1
1418300		14	826505	829045	2540	6 [1]	-	1 [1]
1418500	RPS19	14	831982	832898	916	-	1	-
1419500		14	866832	869255	2423	2 [2]	-	-
1419600	SEC63	14	872333	874417	2084	1 [1]	-	-
1419800		14	881799	883925	2126	2 [2]	-	-
1420200		14	900284	905284	5000	1	-	2
1420300		14	906319	906546	227	-	-	1
1420400		14	909529	915150	5621	-	-	3 [1]
1420600	PRP2	14	922984	926865	3881	-	2	5
1420700		14	927966	929346	1380	-	1	1
1420800		14	930670	932421	1751	-	1	1
1420900		14	933170	935485	2315	-	2	3 [1]
1421000		14	936604	937554	950	3 [2]	-	1 [1]
1421500		14	953387	954285	898	-	1	-
1421700		14	958327	967452	9125	1 [1]	1 [1]	11 [8]
1421800		14	970229	970839	610	-	1	1
1421900		14	971212	980058	8846	-	-	1
1422000	DHHC8	14	981118	983685	2567	-	-	1
1422100		14	984493	989640	5147	-	-	3 [1]
1422200	PDEgamma	14	992097	995967	3870	1	-	1
1422500		14	1003488	1006247	2759	-	1	2 [1]
1422600		14	1006961	1008990	2029	-	1 [1]	3 [1]
1422700	NELFA	14	1010086	1011231	1145	-	-	2
1422800		14	1013271	1023611	10340	-	4 [3]	10 [7]
1423000		14	1033404	1035311	1907	-	1 [1]	-
1423100		14	1037312	1041298	3986	-	-	5 [2]
1423500		14	1053884	1055587	1703	-	1 [1]	1 [1]
1423900		14	1060939	1062312	1373	-	-	1 [1]
1424600		14	1079250	1081568	2318	-	-	2
1424800		14	1083898	1085262	1364	-	1	2
1428700		14	1249548	1251279	1731	-	2 [2]	2 [2]
1428900		14	1256437	1258062	1625	-	-	2 [1]
1429100		14	1265768	1270208	4440	-	2 [2]	6 [3]
1429400	CAF1	14	1282240	1288155	5915	-	4 [1]	21 [10]
1429500	EMC1	14	1293370	1296874	3504	-	2	3 [1]
1429600		14	1298091	1299917	1826	-	-	1
1430000		14	1311770	1312886	1116	-	-	1
1430100		14	1314705	1317431	2726	-	3 [1]	4 [1]
1430200	PPM7	14	1322146	1324781	2635	-	-	2
1430500		14	1336466	1339303	2837	-	-	1
1430600		14	1340429	1341014	585	-	1	3
1430800	JmjC1	14	1347509	1353535	6026	-	-	7 [3]
1430900		14	1355155	1359227	4072	-	-	2 [1]
1431100		14	1364969	1380646	15677	-	2	1 [1]
1432100	EIF3A	14	1434184	1438035	3851	-	3 [2]	4 [3]
1432300	BDP2	14	1445493	1448744	3251	-	1 [1]	1 [1]
1433600	PIGO	14	1494238	1498588	4350	-	5 [3]	6 [4]
1433800		14	1500830	1502587	1757	-	-	1
1433900	EMC6	14	1502756	1503301	545	-	2	2
1434900		14	1525360	1527282	1922	-	1 [1]	1 [1]
1435300		14	1535792	1536895	1103	-	-	1 [1]
1435400	SR10	14	1538844	1540631	1787	-	-	1
1436300	PLP2	14	1565875	1569183	3308	-	4 [1]	5 [1]
1436500		14	1573938	1577162	3224	-	-	1
1436800		14	1583404	1586553	3149	-	-	1
1436900		14	1587957	1590452	2495	-	-	2 [1]
1437000		14	1591014	1593785	2771	-	1 [1]	1 [1]

1437200	APC10	14	1601014	1602012	998	-	-	1 [1]
1437400		14	1604879	1605133	254	-	-	1
1437600	TRAMP	14	1610784	1611806	1022	-	-	1
1437700		14	1613752	1615846	2094	-	2 [1]	7 [5]
1437800		14	1616586	1623359	6773	-	-	1 [1]
1438000		14	1629491	1632232	2741	-	-	5 [2]
1438200		14	1641556	1644501	2945	-	-	2 [1]
1438600		14	1658973	1667243	8270	-	2	5
1438700		14	1670095	1675851	5756	1	1	4
1438800		14	1676976	1682972	5996	-	4 [1]	8 [3]
1439200		14	1691744	1693240	1496	-	2 [2]	2 [2]
1439400		14	1698502	1705272	6770	2 [1]	1 [1]	9 [5]
1439500		14	1706387	1715298	8911	-	3 [1]	4 [2]
1439600	DRN1	14	1715893	1717440	1547	-	-	1 [1]
1439900		14	1723292	1727825	4533	-	19 [1]	24 [3]
1440100	SET10	14	1738997	1744708	5711	-	4 [4]	3 [3]
1440200		14	1748363	1750925	2562	-	-	1
1440400		14	1752972	1756999	4027	-	1 [1]	2 [1]
1440600		14	1764215	1766671	2456	-	2 [2]	3 [3]
1440700		14	1767653	1768993	1340	-	-	3 [1]
1440800		14	1769915	1772579	2664	-	-	1
1440900		14	1773240	1775444	2204	-	-	2 [2]
1441000		14	1776103	1778532	2429	-	-	1 [1]
1441600	DPH6	14	1806342	1809347	3005	-	1	4
1441700	AP2-G	14	1822635	1830449	7814	5 [5]	-	16 [13]
1441900		14	1836630	1837706	1076	1	-	1
1442000		14	1838651	1840512	1861	1	-	3
1442100		14	1841619	1843206	1587	1	-	2
1442300		14	1846063	1848202	2139	-	-	1
1442700		14	1865821	1880179	14358	-	13 [11]	13 [11]
1443000		14	1890515	1891441	926	-	1 [1]	-
1443100		14	1891993	1892682	689	-	1	2 [1]
1443600		14	1916710	1919334	2624	-	2 [1]	1 [1]
1443700		14	1921209	1923112	1903	-	1 [1]	1 [1]
1444000		14	1929696	1933397	3701	-	4 [2]	3 [1]
1445000		14	1965115	1968453	3338	2 [1]	2 [2]	3 [3]
1445100		14	1969790	1973662	3872	2 [1]	1 [1]	10 [3]
1445200		14	1975552	1979201	3649	1	1	1
1445300		14	1979662	1981440	1778	3 [1]	-	-
1445400		14	1982710	1985476	2766	2	2 [1]	-
1445500	HAD3	14	1988369	1989118	749	5	-	-
1445600		14	1990105	1991898	1793	3 [2]	-	1 [1]
1445700	HAD2	14	1996888	1997769	881	-	4 [1]	2 [1]
1445800		14	1998654	2001671	3017	2	1	1
1445900		14	2002367	2006188	3821	-	3 [1]	5 [1]
1446100	RRP9	14	2010922	2012781	1859	-	-	1
1446200	ATX3	14	2014546	2016591	2045	-	-	1
1446300		14	2017640	2019184	1544	-	1	1
1446500		14	2023206	2025347	2141	-	2	5
1446600		14	2027722	2033501	5779	-	11 [6]	31 [10]
1446800		14	2037554	2040166	2612	3	4	4 [1]
1447000		14	2051583	2054273	2690	-	6 [3]	6 [3]
1447100		14	2054813	2057809	2996	-	2	2
1447200		14	2059987	2063337	3350	-	3 [1]	4 [2]
1447300		14	2064409	2065818	1409	-	2 [2]	3 [2]
1447700	NEK1	14	2073634	2076631	2997	-	-	1 [1]
1447800		14	2078987	2082513	3526	-	5 [4]	5 [4]
1447900		14	2083116	2084789	1673	-	-	1
1448000	MSP9	14	2086105	2088228	2123	1 [1]	2 [2]	4 [3]
1448100		14	2091789	2094904	3115	1	2	8 [3]
1448200		14	2095141	2103702	8561	4 [3]	14 [7]	24 [15]
1448300		14	2104939	2110197	5258	-	-	3 [1]
1448400		14	2111112	2115758	4646	9 [2]	3 [2]	4 [1]
1448500	MRP2	14	2116423	2122473	6050	47 [24]	3 [2]	16 [7]
1448600		14	2127080	2129872	2792	1 [1]	-	1 [1]
1448700	MIF	14	2131030	2131603	573	-	-	2
1448800	CCT3	14	2133701	2135456	1755	-	4 [1]	-
1448900		14	2135749	2138604	2855	-	1 [1]	8 [4]

1449100		14	2140227	2142341	2114	-	-	6 [2]
1449200	MyoD	14	2142506	2148633	6127	1	3 [1]	6 [2]
1449300		14	2150093	2156454	6361	-	3	-
1449500	TLAP1	14	2164468	2165592	1124	-	1 [1]	1 [1]
1450000		14	2178872	2180172	1300	-	-	1
1450400		14	2192781	2196526	3745	-	-	3 [3]
1450500	RAB2	14	2198073	2199498	1425	1	-	1
1450600		14	2200400	2201548	1148	-	-	1
1450900		14	2223647	2224075	428	2	-	-
1451300		14	2263160	2268886	5726	6 [4]	-	1
1451400		14	2270743	2273826	3083	-	2	-
1451600	CPN60	14	2276730	2279359	2629	-	1	-
1451700	LPD1	14	2281716	2283407	1691	1	-	1
1451900		14	2285804	2286853	1049	-	-	1 [1]
1452100		14	2292223	2293245	1022	-	-	3 [2]
1452500	EF-G	14	2303972	2306338	2366	-	-	1
1452800		14	2322640	2326143	3503	-	7 [1]	7 [1]
1452900		14	2326706	2329076	2370	-	3	5 [1]
1453000		14	2330955	2344950	13995	-	14 [4]	10 [4]
1453200		14	2347014	2348155	1141	-	2 [1]	2 [1]
1453700		14	2374740	2376745	2005	-	-	1 [1]
1454000	TOC75	14	2382813	2385785	2972	-	1 [1]	2 [1]
1454100		14	2386406	2389724	3318	-	3	3
1454200	SF3B3	14	2392061	2396530	4469	-	1	1
1454900	VP2	14	2444889	2448162	3273	-	1 [1]	1 [1]
1455000	NOT4	14	2451286	2455836	4550	-	6 [3]	6 [3]
1455100		14	2456493	2459543	3050	-	2 [2]	4 [2]
1455300	SHMT	14	2464276	2465906	1630	-	1	-
1455500		14	2469674	2477367	7693	-	1	-
1455600	XAB2	14	2478308	2481712	3404	-	-	1
1455700		14	2482938	2483996	1058	-	1	1
1455800		14	2486075	2487499	1424	-	1	1
1456100		14	2495830	2497979	2149	-	4 [3]	4 [2]
1456600		14	2508475	2509892	1417	-	2	2
1456700	UBA2	14	2512023	2514087	2064	-	1	1
1456800		14	2514591	2520518	5927	-	2	2 [1]
1456900		14	2521835	2526928	5093	-	1 [1]	-
1457200		14	2532952	2538339	5387	-	-	1
1458100	CWC22	14	2566479	2569637	3158	-	2	1
1458600		14	2589443	2591752	2309	3 [1]	-	-
1458700		14	2594912	2596426	1514	-	1	2
1459000	ApiAP2	14	2612617	2619675	7058	2 [2]	2 [2]	9 [8]
1462000		14	2796970	2797728	758	-	1	1
1462100		14	2798628	2800657	2029	-	1	-
1463000		14	2822964	2824319	1355	-	3 [2]	2 [1]
1463400		14	2840766	2845330	4564	-	-	1 [1]
1463500	TFB2	14	2845988	2848819	2831	-	-	2
1463600		14	2853534	2857391	3857	-	1	9 [7]
1463700		14	2857736	2860084	2348	2	-	3
1464300	ARFGAP	14	2905150	2906184	1034	4 [2]	-	1
1464400		14	2908832	2909807	975	2 [1]	-	6 [1]
1464500		14	2910090	2912741	2651	-	2 [1]	3 [1]
1464600		14	2914013	2917172	3159	-	-	2
1464800		14	2920767	2924167	3400	-	2 [1]	4 [2]
1464900		14	2924674	2928096	3422	-	-	1
1465000	UBC12	14	2928652	2929867	1215	-	-	1
1465500		14	2948239	2949162	923	-	3 [1]	3 [1]
1465700	ALAS	14	2952995	2955103	2108	-	1 [1]	1 [1]
1465900		14	2960912	2966152	5240	-	3 [2]	1 [1]
1466000		14	2967681	2972135	4454	-	3	6 [1]
1466100	MTIP	14	2974920	2975537	617	-	2 [1]	2 [1]
1466200		14	2976700	2978247	1547	-	-	1
1466400		14	2981682	2982896	1214	-	-	1 [1]
1467200		14	3004196	3006373	2177	-	1	-
1467700		14	3017605	3018165	560	-	-	1
1467900		14	3021335	3022507	1172	-	1	1
1468100		14	3025622	3026473	851	-	-	2 [2]
1468200		14	3027788	3028660	872	2	-	2

1468400		14	3040341	3046055	5714	3 [2]	10 [7]	12 [7]
1468600	RPT6	14	3048500	3049774	1274	2	1	-
1468700		14	3051408	3053354	1946	3 [1]	1 [1]	-
1468800		14	3054790	3056403	1613	1	-	-
1469000	PPM4	14	3060465	3063863	3398	-	-	4 [2]
1469100		14	3064532	3064954	422	-	1 [1]	-
1469200		14	3065622	3067057	1435	-	-	1
1469300	LRR12	14	3067600	3070140	2540	-	-	2
1469400		14	3072220	3074439	2219	2 [2]	2	4 [3]
1469500	THO2	14	3078660	3087316	8656	-	3	1 [1]
1470100	G377	14	3106730	3114445	7715	4 [1]	10 [6]	9 [4]
1470300	VPS26	14	3123854	3124888	1034	-	-	1
1470400		14	3126094	3128043	1949	1	4 [3]	5 [3]
1470600		14	3131401	3132831	1430	1	-	-
1471000		14	3146569	3148329	1760	-	-	1
1471400		14	3158757	3160124	1367	1	1	-
1471500		14	3161750	3164092	2342	-	-	2 [1]
1471600		14	3166268	3168289	2021	8 [7]	4 [3]	5 [4]
1471700		14	3169186	3171326	2140	1	-	-
1471800		14	3172449	3174569	2120	7 [3]	-	1
1472100	RON3	14	3179903	3188164	8261	43 [14]	4 [3]	1 [1]
1472200		14	3192634	3193032	398	-	-	1
1472300	NBPXa	14	3199216	3207768	8552	57 [40]	12 [10]	-
1472400		14	3208812	3211154	2342	15 [9]	-	2 [2]
1472500		14	3213887	3215143	1256	6 [4]	7 [5]	2 [1]
1472600		14	3217875	3219342	1467	12 [7]	-	1
1472700		14	3222770	3223442	672	4 [1]	-	-
1472800		14	3226448	3227384	936	2 [1]	-	1 [1]

S4 Table. *P. knowlesi* gene ontology (GO) analysis presenting the top 20% of GO terms occurring in genes with at least one fixed SNP (Fst = 1) among comparisons

GO group	GO ID	GO subgroup	Total no. genes	Pen-Pk vs Mf-Pk	Pen-Pk vs Mn-Pk	Mf-Pk vs Mn-Pk
BP	GO:0000413	protein peptidyl-prolyl isomerization	6	0.167	0.833	0.333
	GO:0002098	tRNA wobble uridine modification	3	0.333	0.333	1
	GO:0006289	nucleotide-excision repair	3	0.333	1	0
	GO:0006355	regulation of transcription, DNA-templated	78	0.410	0.808	0.244
	GO:0006396	RNA processing	13	0.231	0.846	0.308
	GO:0006414	translational elongation	3	0	1	0.333
	GO:0006464	cellular protein modification process	6	0.500	0.833	0.333
	GO:0006470	protein dephosphorylation	6	0.500	0.833	0
	GO:0006506	GPI anchor biosynthetic process	3	0.667	1	0
	GO:0006529	asparagine biosynthetic process	3	0.667	1	0
	GO:0006597	spermine biosynthetic process	4	0	0.500	1
	GO:0006614	SRP-dependent cotranslational protein targeting to membrane	3	0	1	0
	GO:0006750	glutathione biosynthetic process	4	0.500	1	0
	GO:0006817	phosphate ion transport	3	0.333	0.333	1
	GO:0006888	endoplasmic reticulum to Golgi vesicle-mediated transport	6	1	1	0
	GO:0006950	response to stress	6	0.167	0.500	0.833
	GO:0007018	microtubule-based movement	43	0.535	0.814	0.186
	GO:0007165	signal transduction	3	0	1	0.333
	GO:0008295	spermidine biosynthetic process	4	0	0.500	1
	GO:0008299	isoprenoid biosynthetic process	4	0.250	1	0
	GO:0008610	lipid biosynthetic process	3	0.333	1	0
	GO:0009058	biosynthetic process	11	0.364	0.818	0.545
	GO:0009103	lipopolysaccharide biosynthetic process	4	0.250	1	0
	GO:0009396	folic acid-containing compound biosynthetic process	3	0	1	0
	GO:0015031	protein transport	8	0.500	0.875	0.125
	GO:0015693	magnesium ion transport	4	0.500	1	0
	GO:0016114	terpenoid biosynthetic process	6	0.333	1	0
	GO:0016567	protein ubiquitination	5	0.600	1	0
	GO:0018342	protein prenylation	4	0	0.500	1
	GO:0019538	protein metabolic process	8	0.500	1	0
	GO:0034227	tRNA thio-modification	3	0.333	0.333	1
	GO:0042147	retrograde transport, endosome to Golgi	4	1	0.500	0
	GO:0043039	tRNA aminoacylation	7	0.429	0.857	0.143
CC	GO:0000439	transcription factor TFIIH core complex	3	0.333	1	0
	GO:0005869	dynactin complex	4	0.500	1	0
	GO:0030127	COPII vesicle coat	6	1	1	0
MF	GO:0000287	magnesium ion binding	7	0.857	0.571	0.286
	GO:0001671	ATPase activator activity	4	0.500	1	0
	GO:0003746	translation elongation factor activity	4	0.250	1	0.250
	GO:0003755	peptidyl-prolyl cis-trans isomerase activity	6	0.167	0.833	0.333
	GO:0003777	microtubule motor activity	43	0.535	0.814	0.186
	GO:0003824	catalytic activity	65	0.308	0.831	0.246
	GO:0003872	6-phosphofructokinase activity	4	0.500	1	0
	GO:0004014	adenosylmethionine decarboxylase activity	4	0	0.500	1
	GO:0004066	asparagine synthase (glutamine-hydrolyzing) activity	3	0.667	1	0
	GO:0004114	3',5'-cyclic-nucleotide phosphodiesterase activity	3	0	1	0.333
	GO:0004222	metalloendopeptidase activity	7	0.429	0.857	0.143
	GO:0004357	glutamate-cysteine ligase activity	4	0.500	1	0
	GO:0004484	mRNA guanylyltransferase activity	3	0	1	0
	GO:0004488	methylenetetrahydrofolate dehydrogenase (NADP+) activity	3	0	1	0
	GO:0004527	exonuclease activity	6	0.833	0.333	0.333
	GO:0004743	pyruvate kinase activity	3	1	0.333	0.667
	GO:0004842	ubiquitin-protein transferase activity	8	0.750	1	0
	GO:0005044	scavenger receptor activity	4	0	1	0.500
	GO:0005198	structural molecule activity	10	0.500	0.900	0.200
	GO:0005315	inorganic phosphate transmembrane transporter activity	3	0.333	0.333	1

GO:0005509	calcium ion binding	79	0.152	0.899	0.215
GO:0008017	microtubule binding	16	0.625	0.875	0
GO:0008080	N-acetyltransferase activity	3	1	1	0
GO:0008081	phosphoric diester hydrolase activity	3	0.333	1	0.333
GO:0008134	transcription factor binding	8	0.375	0.875	0.250
GO:0008138	protein tyrosine/serine/threonine phosphatase activity	6	0.500	0.833	0
GO:0008318	protein prenyltransferase activity	4	0	0.500	1
GO:0008661	1-deoxy-D-xylulose-5-phosphate synthase activity	4	0.500	1	0
GO:0015035	protein disulfide oxidoreductase activity	3	0.333	1	0
GO:0015078	proton transmembrane transporter activity	3	0.333	1	0
GO:0015095	magnesium ion transmembrane transporter activity	4	0.500	1	0
GO:0015930	glutamate synthase activity	4	0.500	1	0
GO:0016616	oxidoreductase activity, acting on the CH-OH group of donors, NAD or NADP as acceptor	6	0.500	0.833	0.333
GO:0016747	transferase activity, transferring acyl groups other than amino-acyl groups	5	0.400	1	0
GO:0016868	intramolecular transferase activity, phosphotransferases	4	0	1	0
GO:0019843	rRNA binding	3	0.667	1	0
GO:0030955	potassium ion binding	3	1	0.333	0.667
GO:0042578	phosphoric ester hydrolase activity	3	0.667	1	0
GO:0046872	metal ion binding	44	0.500	0.864	0.068
GO:0051082	unfolded protein binding	6	0.167	0.500	1
GO:0051087	chaperone binding	4	0.500	1	0
GO:0051536	iron-sulfur cluster binding	8	0.500	0.875	0

BP - Biological processes; CC - Cellular Component; MF - Molecular function.

Supplementary figures

S1 Figure. Multiplicity of infection Fws values for *P. knowlesi* isolates grouped by known clusters (Mf-Pk, Mn-Pk and Pen-Pk).

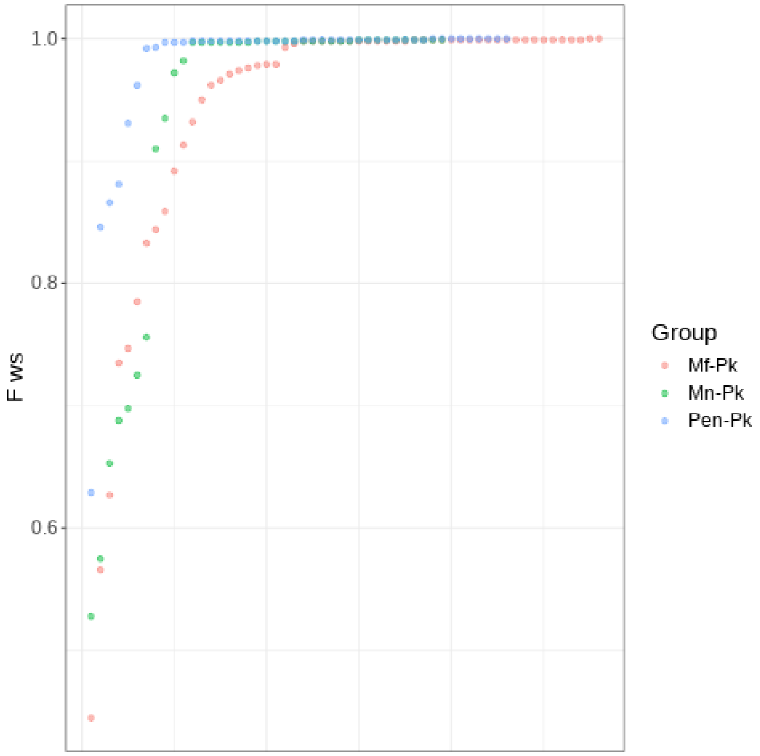

S2 Figure. Pairwise identity-by-descent (IBD)-fractions across sub-populations of *P. knowlesi* (Mf-Pk, Mn-Pk and Pen-Pk)

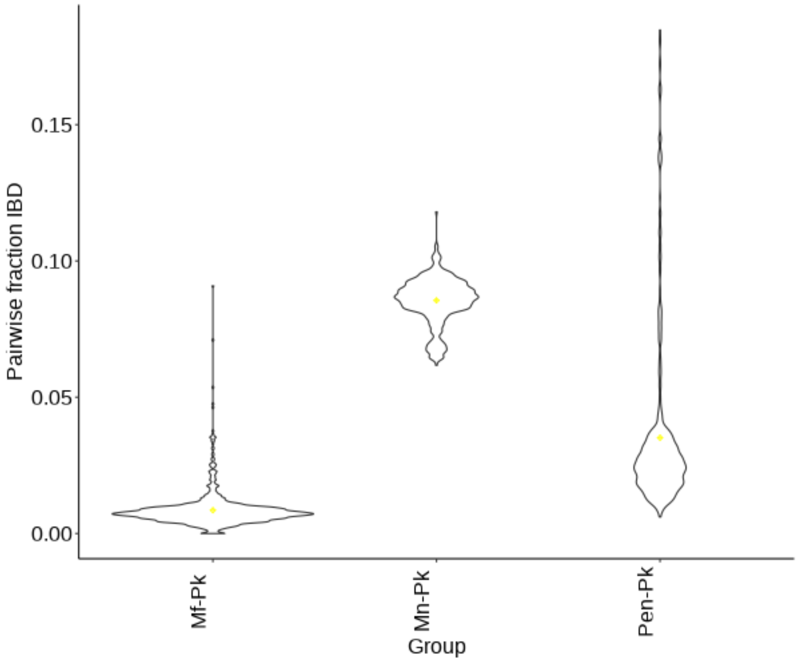

S3 Figure. The neighbour-joining trees for regions with evidence of exchange events among Mn-Pk and Mf-Pk groups (A) Chromosome 5, 600kbp-700kbp; (B) Chromosome 8, 600kbp-800kbp; (C) Chromosome 8, 850kbp-1400kbp; (D) Chromosome 8, 1500kbp-1700kbp; (E) Chromosome 11, 100kbp-300kbp; (F) Chromosome 11, 500kbp-700kbp; (G) Chromosome 11, 1800kbp-1900kbp; (H) Chromosome 11, 2000kbp-2200kbp

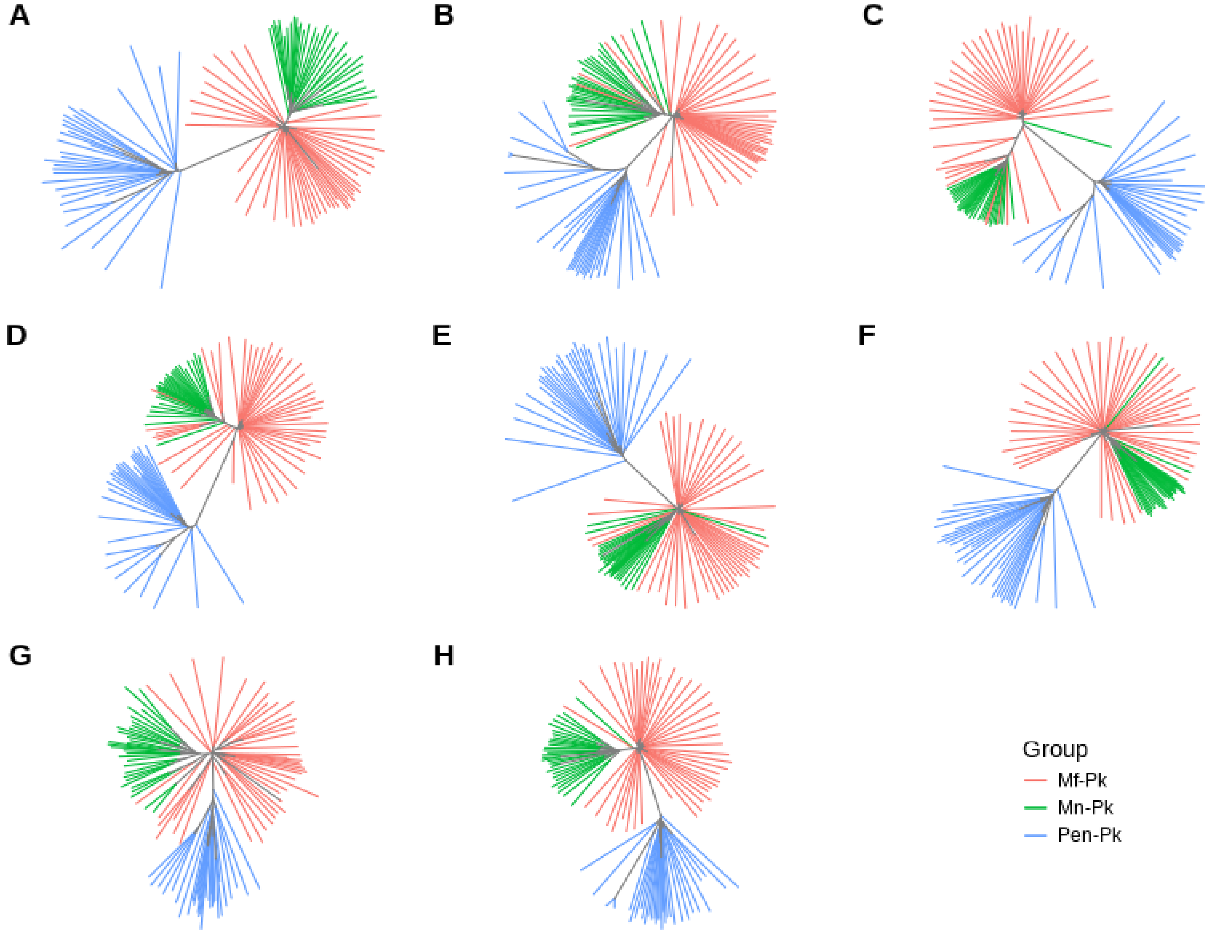

S4 Figure. Neighbour-joining trees and UMAP clustering for loci with signatures of genetic exchange. (A) Chromosome 7, 1400kbp - 1500kbp (same as Figure 5 B,C from main publication); (B) Chromosome 12, 2000kbp - 2300kbp; (C) Chromosome 13, 1100kbp - 1300kbp.

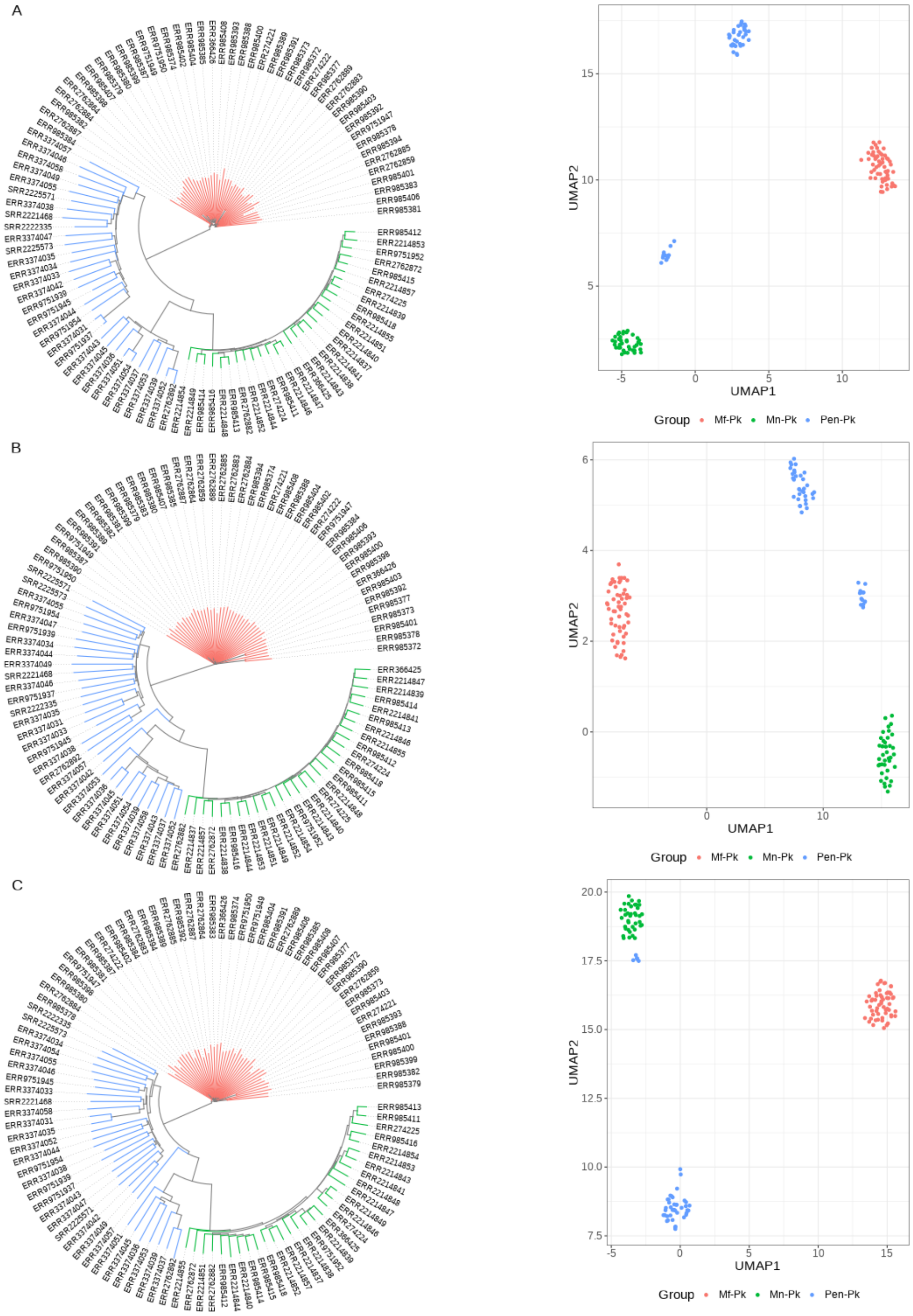

S5 Figure. Neighbour-joining trees for invasion genes. Duffy Binding Proteins ((A) DBPα, (B) DBPβ, (C) DBPγ) and Normocyte Binding Proteins ((D) NBPXα, (E) NBPXβ).

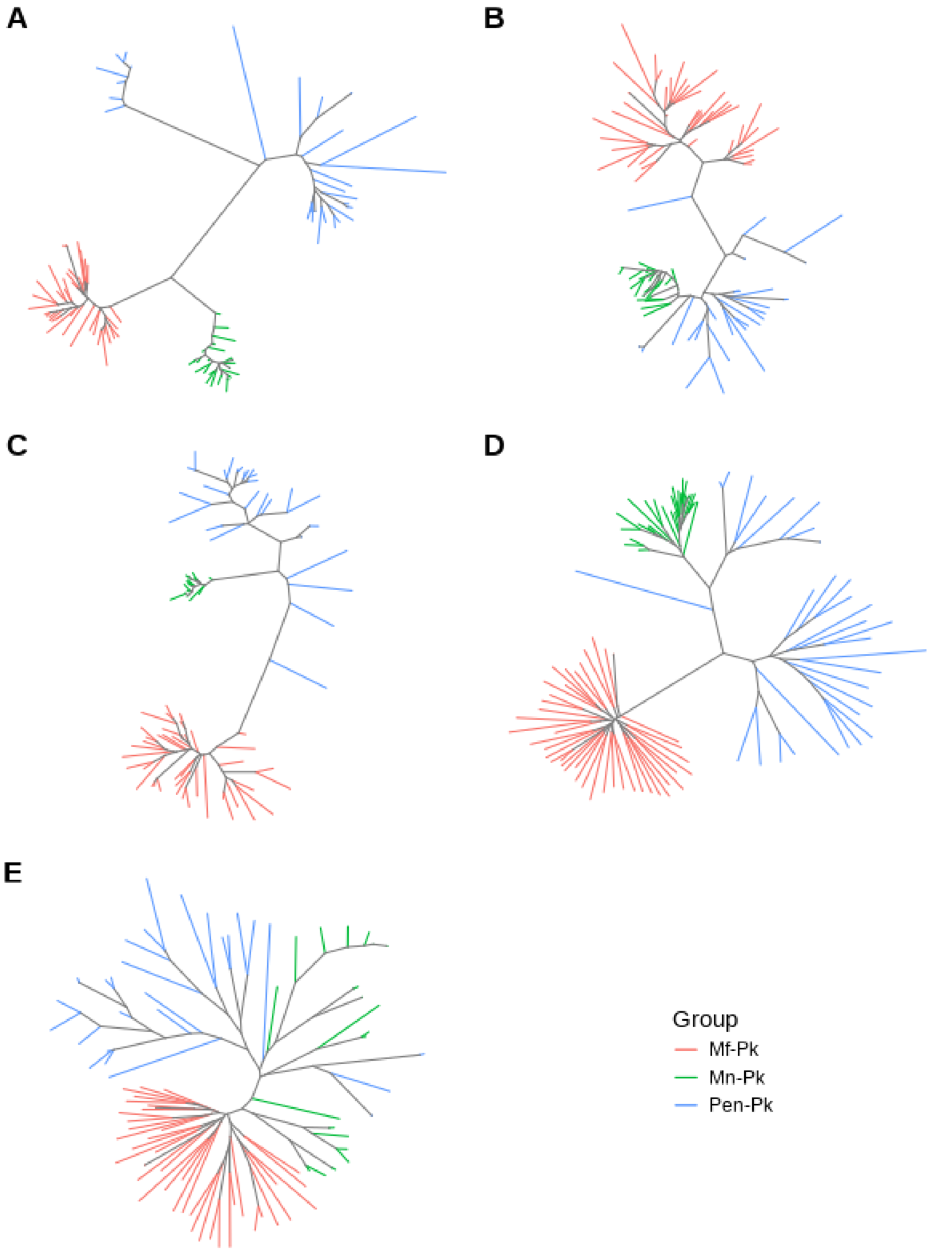

S6 Figure. Evidence of selective sweeps Manhattan plots showing the genome-wide results of the iHS analysis on *P. knowlesi* from all studies groups (A, B, C); Rsb analysis for *P. knowlesi* for pairwise comparison between all clusters (D, E, F).

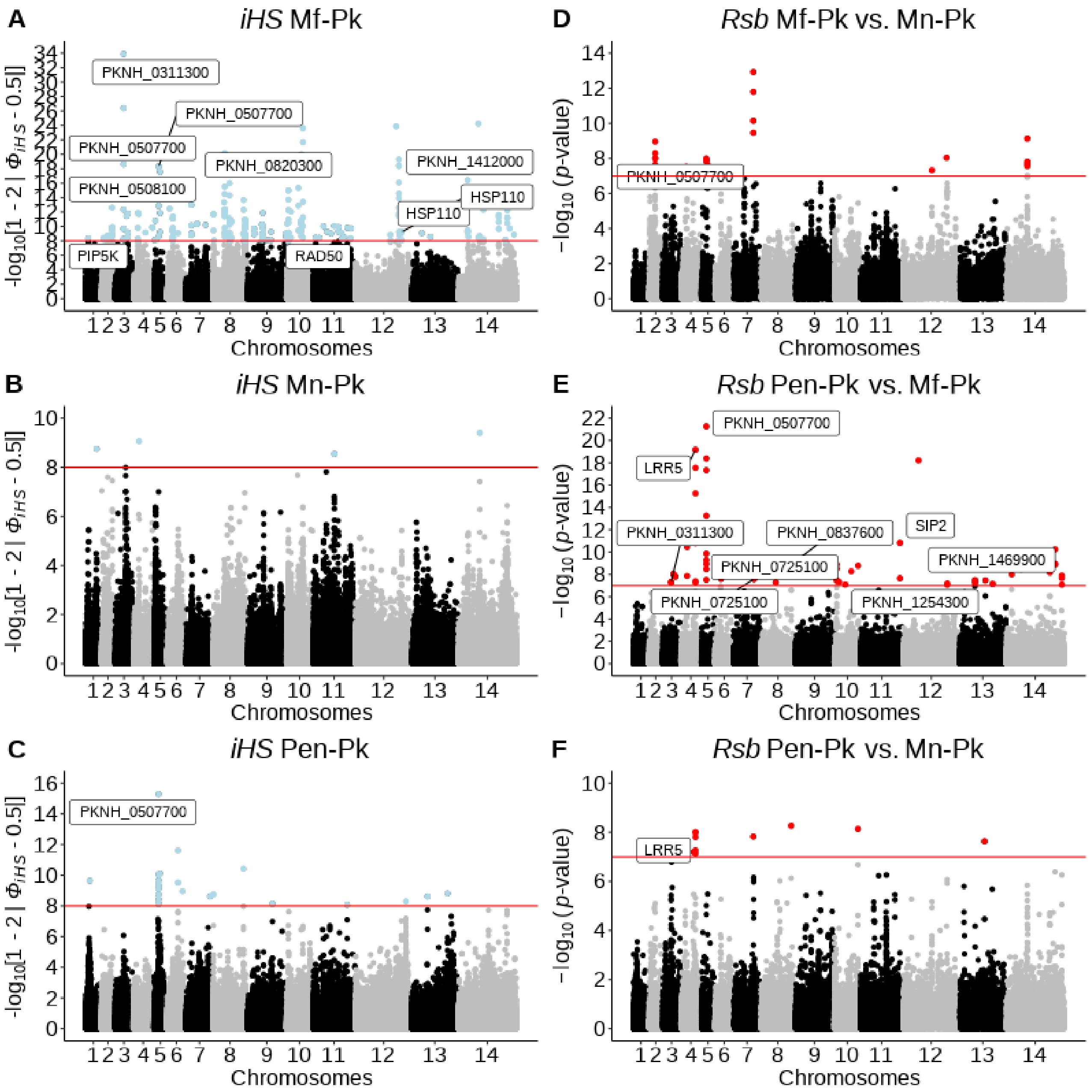

References

[1] Divis, P. C., Duffy, C. W., Kadir, K. A., Singh, B. & Conway, D. J. Genome-wide mosaicism in divergence between zoonotic malaria parasite subpopulations with separate sympatric transmission cycles. Mol. Ecol. 27, 860–870, DOI: 10.1111/mec.14477 (2018).

[2] Pinheiro, M. M. et al. Plasmodium knowlesi genome sequences from clinical isolates reveal extensive genomic dimorphism. PLoS ONE 10, 1–16, DOI: 10.1371/journal.pone.0121303 (2015).

[3] Hocking, S. E., Divis, P. C., Kadir, K. A., Singh, B. & Conway, D. J. Population genomic structure and recent evolution of Plasmodium knowlesi, Peninsular Malaysia. Emerg. Infect. Dis. 26, 1749–1758, DOI: 10.3201/eid2608.190864 (2020).

[4] Assefa, S. et al. Population genomic structure and adaptation in the zoonotic malaria parasite Plasmodium knowlesi. Proc. Natl. Acad. Sci. United States Am. 112, 13027–13032, DOI: 10.1073/pnas.1509534112 (2015).

[5] Benavente, E. D. et al. Whole genome sequencing of amplified Plasmodium knowlesi DNA from unprocessed blood reveals genetic exchange events between Malaysian Peninsular and Borneo subpopulations. Sci. Reports 9, 1–11, DOI:10.1038/s41598-019-46398-z (2019).
